# Uncovering genomic regions controlling plant architectural traits in hexaploid wheat using different GWAS models

**DOI:** 10.1038/s41598-021-86127-z

**Published:** 2021-03-24

**Authors:** Ali Muhammad, Jianguo Li, Weichen Hu, Jinsheng Yu, Shahid Ullah Khan, Muhammad Hafeez Ullah Khan, Guosheng Xie, Jibin Wang, Lingqiang Wang

**Affiliations:** 1 State Key Laboratory for Conservation and Utilization of Subtropical Agro-Bioresources, College of Agriculture, Guangxi University, 100 Daxue Rd., Nanning, Guangxi China; 2 College of Plant Science and Technology & Biomass and Bioenergy Research Center, Huazhong Agricultural University, Wuhan, 430070 China; 3 College of Agriculture and Food Science, Zhejiang A&F University, Lin’an, 311300 China; 4 Department of Agriculture, Abdul Wali Khan University Mardan, Mardan, Pakistan; 5 National Key Laboratory of Crop Genetic Improvement, Huazhong Agricultural University, Wuhan, 430070 China

**Keywords:** Molecular biology, Plant sciences

## Abstract

Wheat is a major food crop worldwide. The plant architecture is a complex trait mostly influenced by plant height, tiller number, and leaf morphology. Plant height plays a crucial role in lodging and thus affects yield and grain quality. In this study, a wheat population was genotyped by using Illumina iSelect 90K single nucleotide polymorphism (SNP) assay and finally 22,905 high-quality SNPs were used to perform a genome-wide association study (GWAS) for plant architectural traits employing four multi-locus GWAS (ML-GWAS) and three single-locus GWAS (SL-GWAS) models. As a result, 174 and 97 significant SNPs controlling plant architectural traits were detected by ML-GWAS and SL-GWAS methods, respectively. Among these SNP makers, 43 SNPs were consistently detected, including seven across multiple environments and 36 across multiple methods. Interestingly, five SNPs (Kukri_c34553_89, RAC875_c8121_1490, wsnp_Ex_rep_c66315_64480362, Ku_c5191_340, and tplb0049a09_1302) consistently detected across multiple environments and methods, played a role in modulating both plant height and flag leaf length. Furthermore, candidate SNPs (BS00068592_51, Kukri_c4750_452 and BS00022127_51) constantly repeated in different years and methods associated with flag leaf width and number of tillers. We also detected several SNPs (Jagger_c6772_80, RAC875_c8121_1490, BS00089954_51, Excalibur_01167_1207, and Ku_c5191_340) having common associations with more than one trait across multiple environments. By further appraising these GWAS methods, the pLARmEB and FarmCPU models outperformed in SNP detection compared to the other ML-GWAS and SL-GWAS methods, respectively. Totally, 152 candidate genes were found to be likely involved in plant growth and development. These finding will be helpful for better understanding of the genetic mechanism of architectural traits in wheat.

## Introduction

Wheat (*Triticum aestivum* L.) is a staple crop worldwide, providing 20% of total food needs of the world population^1,2^. Plant architecture is a complex trait mainly depends on the three dimensional structure of the plant stature including branching pattern, morphology of leaves and flower organs^3^. Plant height directly indicates the ability of plant to compete for light, influencing plant growth and development^4^. It is of prime importance, strongly influencing plant defense against environmental stress, potential grain yield, and plant adaptability for better cultivation and harvesting^5,6^. Leaf morphology can regulate many important aspects related to plant growth and development^7^. In cereals, flag leaves have prominent role in photosynthesis and contribute about 43% of the total carbohydrates required for gain filling^8^. To date, several loci have been identified associated with flag leaf related traits in cereals^9,10^. Productive tillers in wheat are of great importance which may directly affect spike number and thus influence the final yield. The plant stature and the number of tillers influence many factors, including the process of photosynthesis, the flowering and grain set in plant^11^. It is thus understandable that the genetic elucidation of tillers at various plant growth stages is an important component in wheat breeding research programs^11^.

Considerable work has been done to dissect the genetic background of plant height in wheat. To date, 25 height reducing genes have been identified across different chromosomes in wheat^12,13^. One of the great achievements of the Green Revolution, was mainly based on modifying plant architecture by selecting the cultivars with reduced height that can carry more yield with enhanced resistance against lodging^14^. Achieving optimal plant height is of prime importance for the stability, productivity and yield potential of the cultivars^15,16^. Improvement in wheat yield during the Green Revolution was achieved through the introduction of Reduced height (Rht) dwarfing genes. Among them, the *Rht-B1* and *Rht-D1* loci ensured short stature by limiting the response to the growth-promoting hormone gibberellin (GA). In addition, a newly discovered gene for reduced height *Rht24* belongs to GA-sensitive type and was predicated to be commercial importance in worldwide wheat breeding^17^. Till now, more than 50 loci have been detected for plant height^16,17^. For instance, Wei et al.^18^, detected several stable SNPs on chromosomes 2A, 2B, 2D, 3B, 4B, 5A, 5D, 7B, and 7D associated with plant height. Griffiths et al.^16^, identified several height related genes on all chromosomes except for 3D, 4A, and 5D. Further studies are required to discover, to fine map and to clone more new semi-dwarf genes thus expanding the portfolio of Rht genes for the breeding of the favorable plant architecture.

Currently research has been accomplished through GWAS in wheat^9^, maize^19^, rice^20^, and cotton^21^. Comparatively hexaploid wheat has larger genome (≈ 17.9 Gb) than rice (≈ 400 Mb) and maize (≈ 2.32 Gb)^9^. Over the past decade, the absence of fully sequenced reference genome has limited the gene discovery of wheat. Recent advances in functional genomics have provided breeders with a new impetus to achieve their goals^22^. However, substantially more work is required because of the experimental bottleneck emerging from the absence of inconsistency among studies, and the utilization of low-density marker platforms in gene mapping studies. Despite the current research on plant height in wheat, the effects of important loci and several other candidate loci responsible for fine-tuning of plant height in hexaploid wheat is still an intrinsic part in wheat breeding^17^. The availability of high quality reference genome allows for previously impossible follow-up analysis. The application of SNPs as molecular markers provides better understanding of variation in an organism or individual part and further provide high-throughput maps for detecting candidate loci and genes for target traits. Molecular markers are mostly used in segregation analysis, forensic examination, genetic mapping and diagnosis, and numerous biological applications^23–25^. In the present study we were interested to gain a comprehensive picture about the candidate loci responsible for modulating plant height and related traits in wheat through a series of different GWAS models.

Thus the present study was designed to conduct GWAS in a set of 319 wheat accessions employing high-density wheat iSelect 90K SNP array. The objectives of this study included: (1) the investigation of marker-trait associations (MTAs) for plant architectural traits (2) appraising the correlation among these traits and further highlighting SNPs common to more than one trait (3) detecting candidate genes responsible for corresponding morphological traits. Overall, this study will provide insights by integrating three single-locus and four newly developed multi-locus GWAS methods, and will be helpful to establish a regulatory network in the genetic improvement of wheat architectural traits.

## Results

### Statistical analysis of phenotypic traits

**Figure 1:**
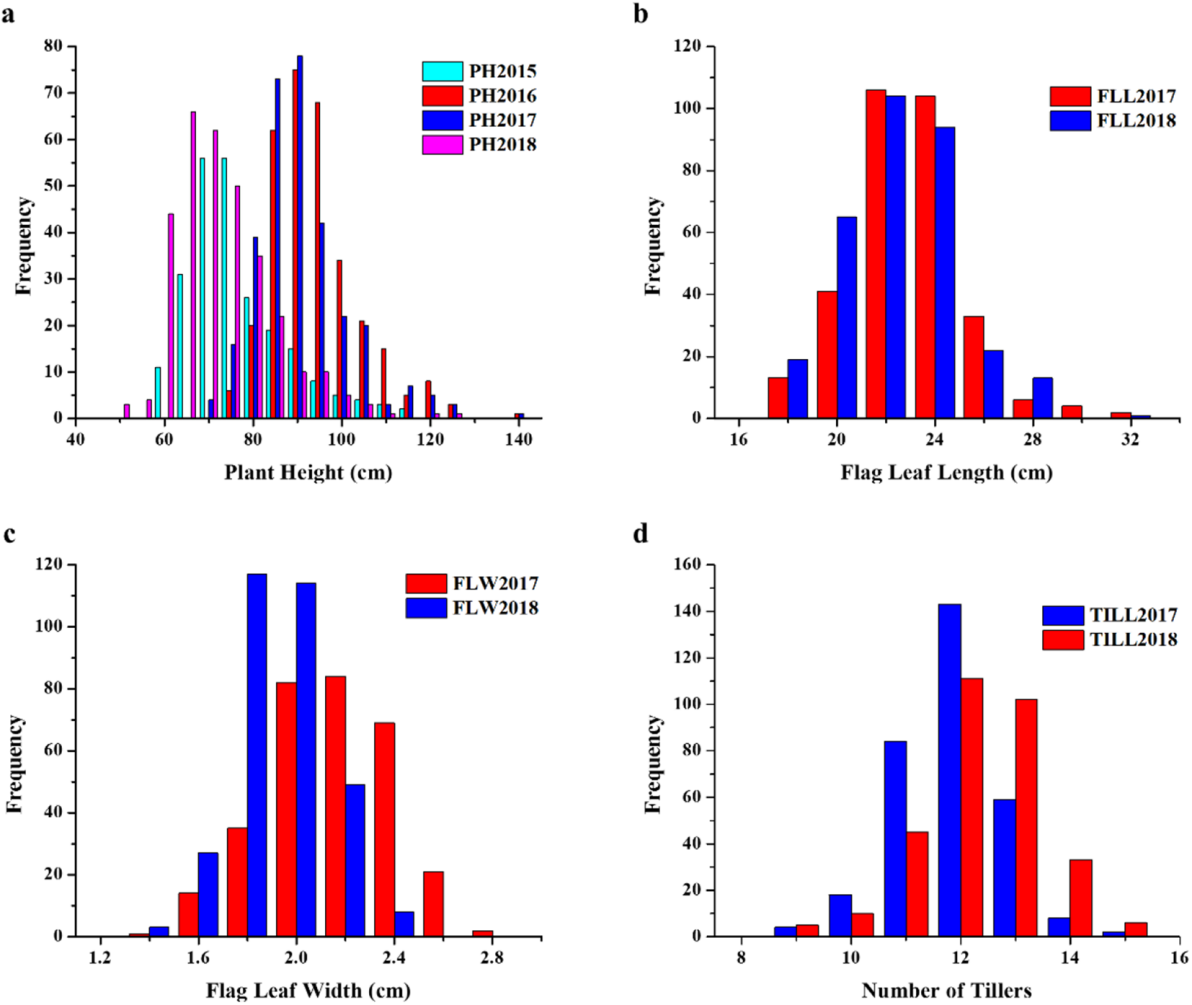
Phenotype distributions for plant architectural traits (**a**) Plant height across four environments (2015, 2016, 2017, and 2018); (**b**) Flag leaf length (2017 and 2018); (**c**) Flag leaf width (2017 and 2018); (**d**) Number of tillers per plant (2017 and 2018).

In the present study, we evaluated wheat germplasm collections for plant architectural traits, including plant height (PH), flag leaf length (FLL), flag leaf width (FLW) and the number of tillers per plant. The phenotypic characteristics for plant height across four environments and flag leaf length, flag leaf width and number of tillers per plant across two environments as shown in Supplementary Table S1 and Supplementary Fig. S1. All the traits exhibited the normal distribution pattern each year, indicating the quantitative nature of these traits (Fig. 1). Descriptive statistics revealed large phenotypic variations for all the traits as given in Supplementary Table S1. PH ranged from 48.40 to 124.82 cm with coefficient of variations (CVs) ranged from 11.09 to 16.11%. The FLL varied from 16.06 to 31.57 cm, FLW varied from 1.22 to 2.75 cm and for tillers, the number of tillers per plant ranged from 8.10 to 14.67. Analysis of variance indicated highly significant differences (*P* < 0.001) for all the studied traits (Supplementary Table S2).

**Figure 2:**
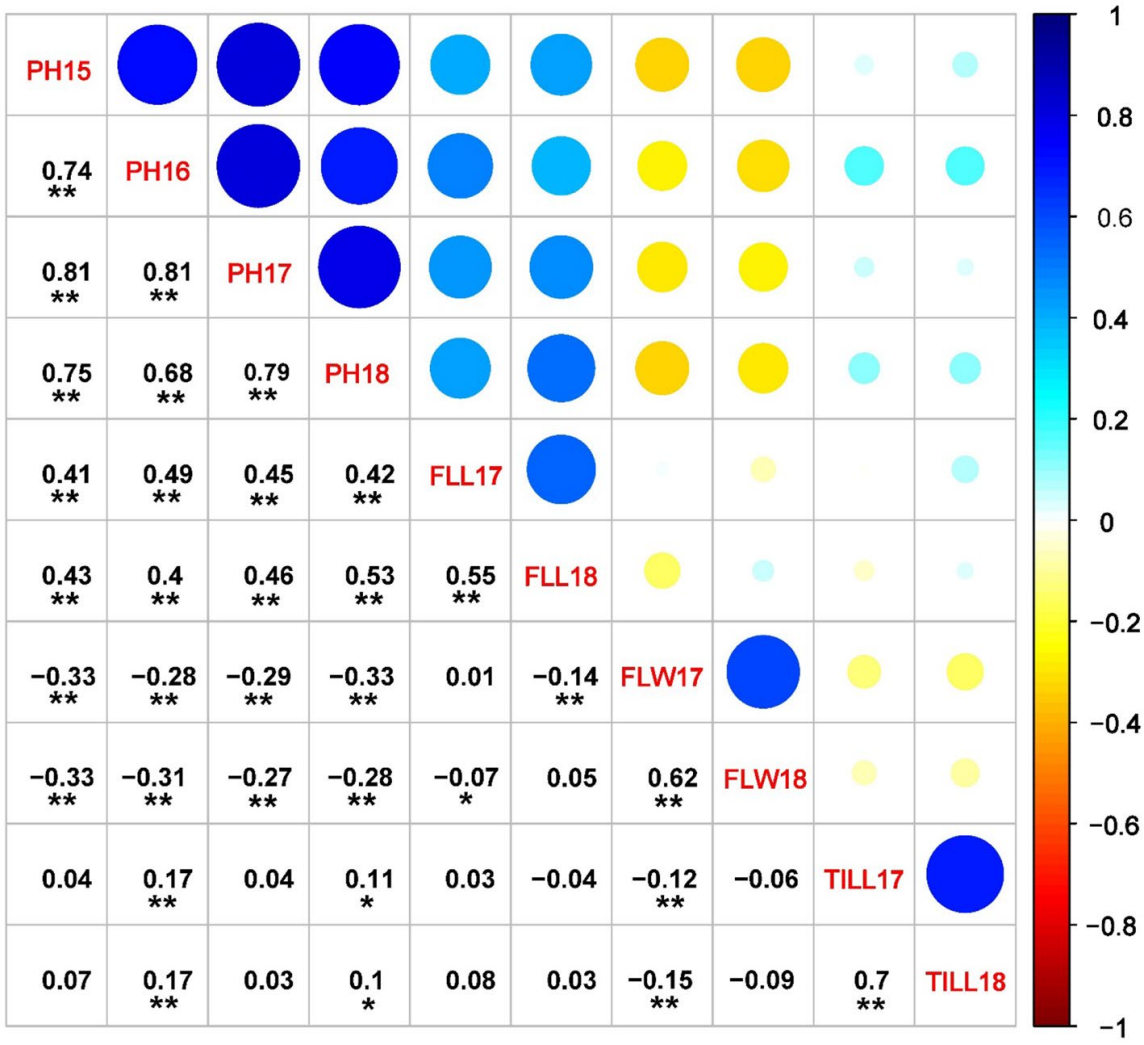
Correlation among plant architectural traits across multiple environments. PH-Plant height (2015–2018), FLL-Flag leaf length (2017–2018), FLW-Flag leaf width (2017–2018) and TILL-Number of tillers per plant (2017–2018). * and ** indicate significant correlation at *P* < 0.05 and 0.01, respectively.

Broad sense heritability was also estimated for PH, FLL, FLW and number of tillers with values ranged from 0.79 (FLL) to 0.91 (PH), suggesting the stability of these traits. The correlation analysis revealed significant correlation between different environments for each of the four traits, indicating the consistency of these traits across various environments (Fig. 2). Furthermore, PH was significantly and positively correlated with FLL in almost all the environments. These results are further confirmed by GWAS results, which revealed several SNPs have common association to both PH and FLL. However, both PH and FLL significantly but negatively correlated with FLW in most of the environments indicating competition of these traits to assimilate at the plant growth stage. Finally, the relatively weak correlation of tillers with other traits suggested the independency of this trait.

### Population structure analysis

Population structure is important due to the large number of diverse genotypes used in the study may produce false associations between the phenotypic values and unlinked markers. Therefore, a comprehensive analysis of population structure is prerequisite for evaluating successful association mapping. The number of subpopulations were calculated by the rate of change in the log probability of data between successive *K*-values. Δ*K* was calculated for increasing the number of *K*-value determined by STRUCTURE analysis according to the procedure of Evanno^26^. At *K* = 2, a break in the slope was observed followed by flattening of the curve (Supplementary Fig. S1a). Hence, the most likely number of subpopulations was two (*K* = 2) (Supplementary Fig. S1b). Moreover, this result was confirmed by PCA based on standardized covariance of genetic distances of SNP markers (Supplementary Fig. S1c). Linkage disequilibrium (LD) analysis indicating the mapping resolution and robustness was done using TASSEL v.5.0. software. LD for whole genome presented in (Supplementary Fig. S2a). The *r*^2^ value for the A, B, and D sub-genomes decreased gradually with increasing the genetic distance (Supplementary Fig. S2b). The LD analysis for the A, B, and D sub-genomes indicated the highest marker density on B (58%) followed by A (34.6%) and then D (7.4%). Among the chromosomes, 2B has the highest marker density, while 4D has the lowest. More details about the description of LD and population structure analysis have been reported in our previous study^27^.

### GWAS using four multi-locus models

**Figure 3:**
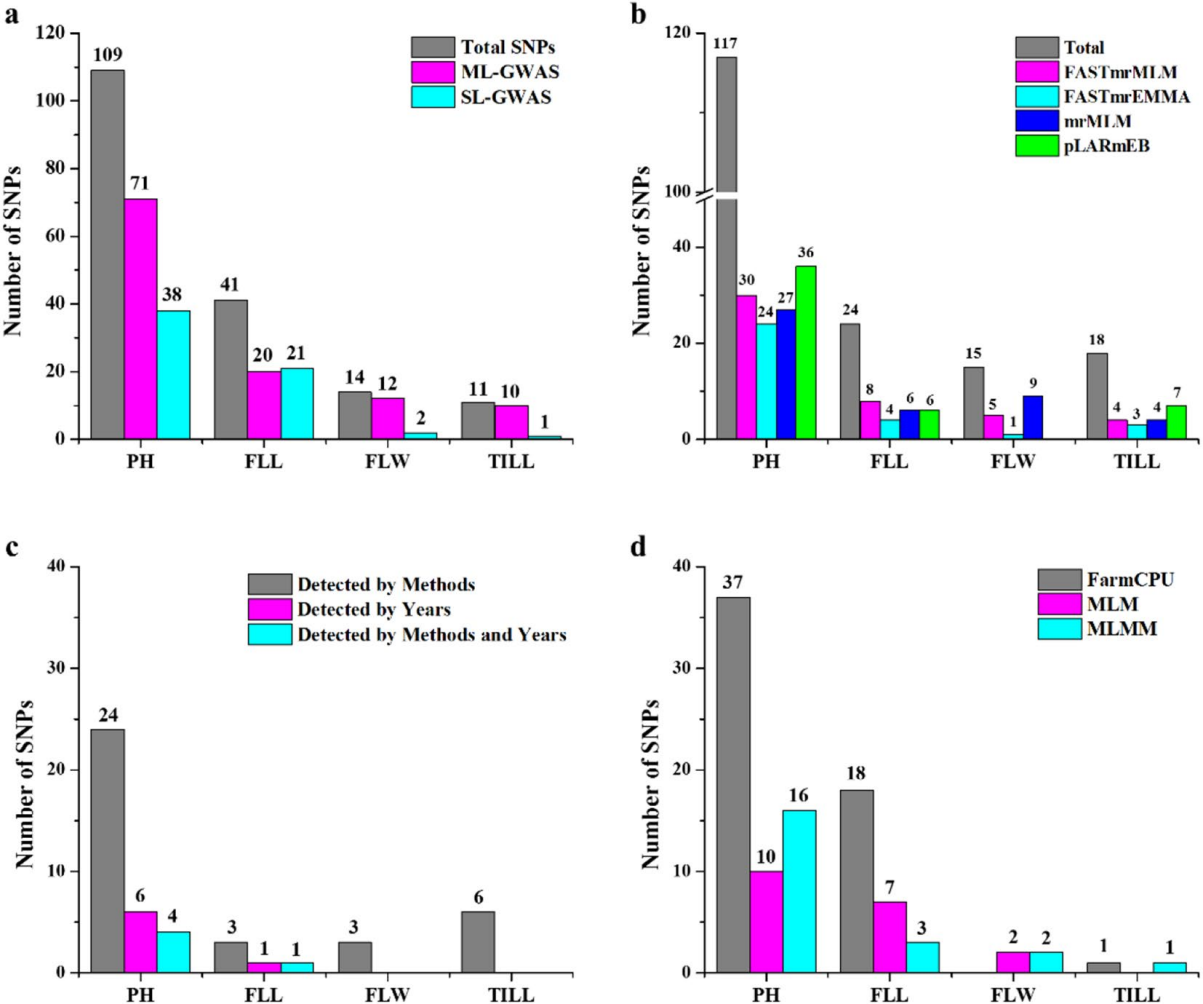
Significant SNPs detected through different GWAS methods in multiple environments. The traits include PH (Plant height); FLL (Flag leaf length); FLW (Flag leaf width) and TILL (Number of tillers per plant). (**a**) Significant SNPs detected via ML-GWAS and SL-GWAS methods, (**b**) Significant SNPs detected through four ML-GWAS methods i.e. FASTmrMLM, FASTmrEMMA, mrMLM, and pLARmEB, (**c**) Significant SNPs detected across multiple years and different ML-GWAS methods, (**d**) Significant SNPs detected through three SL-GWAS methods i.e. FarmCPU, MLM, and MLMM.

To obtain more reliable results, the SNPs that were simultaneously detected in at least two years or by at least two methods were considered as most stable SNPs. After removing the repeated SNPs, a total of 113 and 62 significant SNPs were identified by ML-GWAS and SL-GWAS methods, respectively (Fig. 3a).

**Figure 4:**
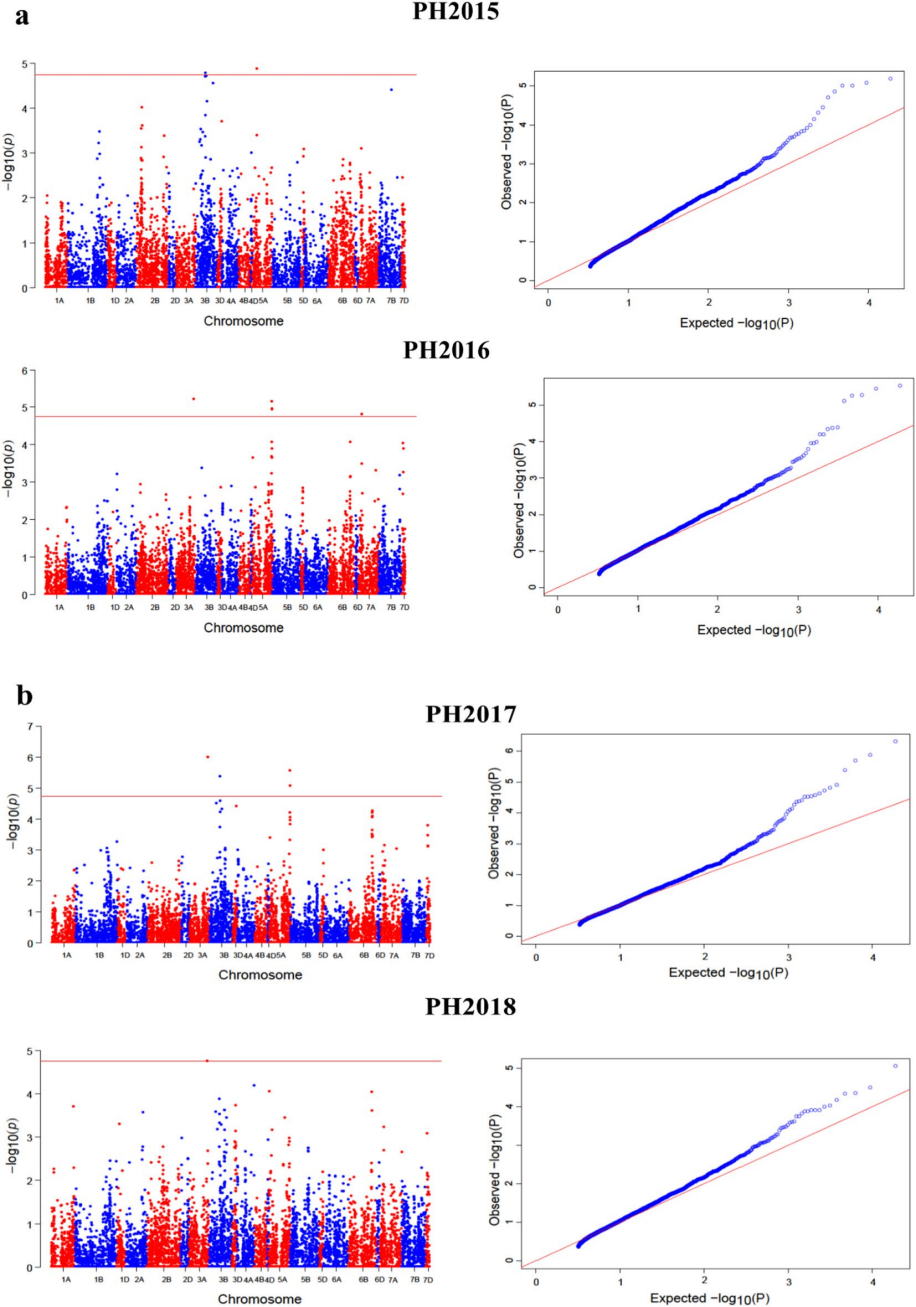

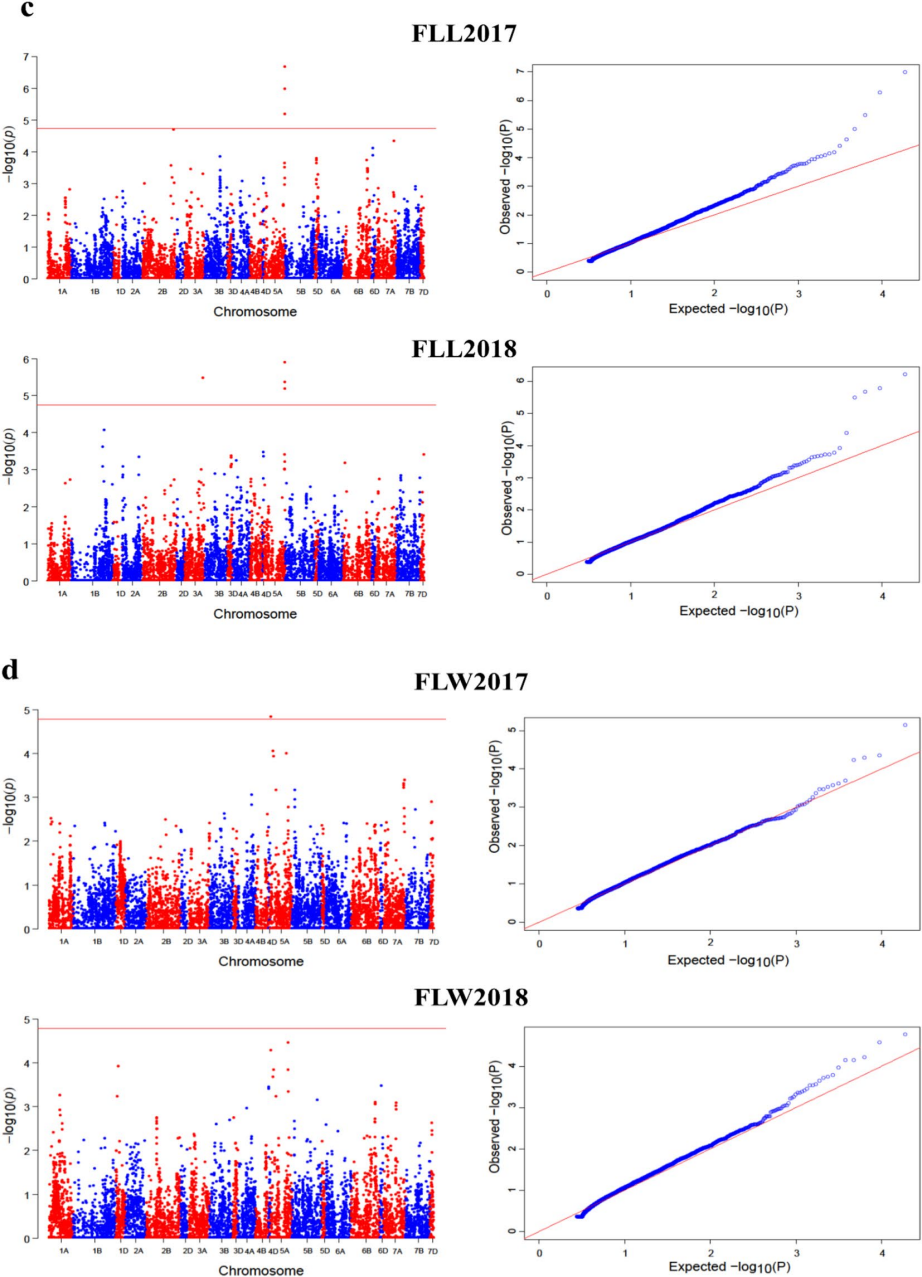

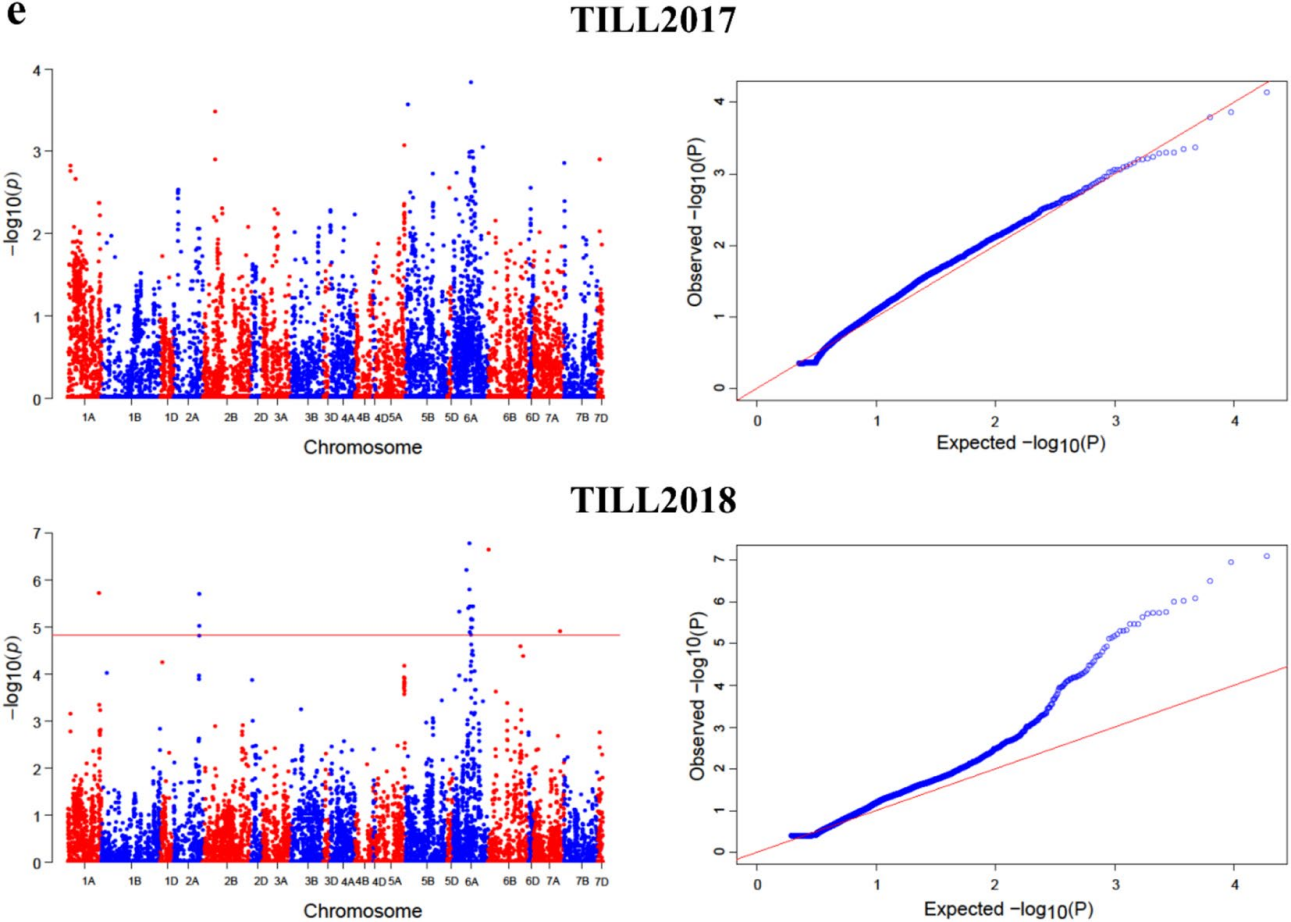
Manhattan and quantile–quantile (Q-Q) plots for plant architectural traits across different environments; (**a**,**b**) Manhattan and Q-Q plots for Plant height (2015–2018); (**c**) Manhattan and Q-Q plots for flag leaf length (2017–2018); (**d**) Manhattan and Q-Q plots for flag leaf width (2017–2018); (**e**) Manhattan and Q-Q plots for number of tillers (2017–2018). Note: Number of detected SNPs by four multi-locus GWAS methods. For brevity, we presented here the Manhattan and Q–Q plots of mrMLM model. The rest of the Manhattan and Q–Q plots are given in Supplementary Fig. S3.

ML-GWAS models i.e. FASTmrMLM, FASTmrEMMA, mrMLM, and pLARmEB screened 47, 32, 46, and 49, significant SNPs, respectively for PH (117 SNPs) across four environments and for FLL (24), FLW (15) and Tillers (18) across two environments (Fig. 4, Tables 1, 2, 3). Among these SNPs, 30, 8, 5, and 4 were detected by FASTmrMLM for PH, FLL, FLW, and Tillers, respectively (Fig. 3b, Table 1). By using FASTmrEMMA the number of significant SNPs identified for the above-mentioned traits were 24, 4, 1, and 3, respectively. Also 27, 6, 9, and 4 significant SNPs were detected by mrMLM approach for the said traits, respectively. Finally, the pLARmEB model identified 36, 6, and 7 significant association signals with PH, FLL, and Tillers, respectively (Fig. 3b, Table 1).

**Table 1:** Common SNPs detected through different ML-GWAS methods associated with plant architectural traits.

Trait	Year	Methods (1–4)	SNP	Chr	Position (bp)	LOD > 3	r^2^ (%)
PH	2015	2, 4	D_contig10675_778	2D	958016	3.1–3.58	1.31–2.35
	2015	1, 2, 4	BobWhite_24364_73	3B	1551850	3.84–5.19	2.41–5.93
	2015	1, 2	wsnp_Ex_rep_c67296_65839761	4D	2143869	3.38–7.15	5.21–6.17
	2015	1, 2	wsnp_Ex_c39592_46849607	5A	2274396	3.20–3.94	1.35–3.70
	2015	2, 4	BS00089076_51	5A	2414392	3.47–4.94	1.49–3.53
	2015	1, 3	RAC875_c34971_137	7A	3550660	5.21–9.59	4.03–9.99
	2016	2, 4	Kukri_04598_614	2A	87562	4.26–6.13	2.25–2.38
	2016	1, 4	Kukri_c34553_89	2B	887912	3.22–6.43	2.32–3.42
	2016	1, 2, 3, 4	RAC875_c8121_1490	3A	1272572	4.98–9.25	5.67–9.34
	2016	1, 4	wsnp_Ex_rep_c66315_64480362	6B	3350805	3.12–6.78	3.10–5.73
	2016	1, 2, 4	Ku_c5191_340	6B	3397322	4.54–7.23	4.23–8.44
	2016	2, 3, 4	Excalibur_03094_523	7D	4302086	4.27–7.19	2.22–5.60
	2017	1, 2, 4	RAC875_c33937_211	1B	1022	3.46–4.25	1.26–3.56
	2017	1, 2	Kukri_c34553_89	2B	887912	3.40–4.60	1.95–3.04
	2017	1, 2, 3	RAC875_c8121_1490	3A	1272572	7.07–7.81	7.44–13.88
	2017	1, 2	RAC875_232_1895	5A	2256162	3.88–4.22	1.53–2.29
	2017	1, 4	Ku_c5191_340	6B	3397322	7.92–9.30	4.10–12.99
	2017	2, 3, 4	GENE_4469_430	7A	3542226	3.69–5.51	2.01–3.09
	2018	3, 4	Kukri_2596_146	3A	1287688	3.03–3.14	0.43–1.53
	2018	1, 2, 3, 4	Ku_27771_508	3B	1402891	4.21–8.20	1.42–8.38
	2018	3, 4	wsnp_Ex_25573_34834321	3B	1536039	3.98–5.66	0.97–2.04
	2018	1, 2, 4	Excalibur_01167_1207	5A	2153585	3.00–4.62	0.54–3.24
	2018	1, 2, 3, 4	Tdurum_contig42962_2138	5A	2426777	4.96–6.13	1.51–7.82
	2018	1, 2, 4	RAC875_c6911_412	7A	3921772	4.29–6.33	0.82–4.45
FLL	2017	1, 2	BobWhite_c5694_1201	4B	2087004	3.73–5.13	4.29–5.65
	2017	1, 2	tplb0049a09_1302	5A	2453837	4.96–7.17	6.61–13.14
	2017	2, 4	BS00021881_51	6D	3496747	4.384.59	1.98–2.97
FLW	2017	1, 2	IAAV8465	3D	1660873	3.70–4.19	5.48–5.77
	2017	1, 2	RAC875_01969_384	7A	3547319	3.05–6.53	5.13–7.53
	2018	1, 3	BS00068592_51	5B	2485486	3.79–5.65	4.95–5.37
TILL	2017	1, 4	TA001732_0977	2B	580007	3.10–3.96	1.15–5.85
	2017	2, 3	wsnp_BE499835B_Ta_2_5	5B	2481742	3.81–4.51	3.64–3.99
	2017	1, 4	Kukri_c4750_452	6A	2998535	3.23–3.46	1.59–6.30
	2017	1, 2, 3, 4	BS00022127_51	7B	3924027	3.11–5.15	2.60–5.56
	2017	2, 4	D_contig07330_330	7D	4292747	3.20–3.75	1.16–2.06
	2018	1, 3	BobWhite_07783_174	2A	359541	4.11–4.27	4.18–5.93

Methods (1–4): corresponding four multi-locus GWAS methods i.e. mrMLM, FASTmrMLM, FASTmrEMMA, and pLARmEB, respectively. r^2^ (%) represents proportion of total phenotypic variation explained by each SNP. PH (plant height); FLL (flag leaf length); FLW (flag leaf width); TILL (number of tillers per plant).

To validate the findings, we further compared the results across multiple environments and found six and one SNPs were co-identified in at least two of the environments for PH and FLL, respectively (Fig. 3c, Table 2). These environment-stable SNPs were located on different chromosomes. For PH there was one SNP on chromosome 2B, one on 3A, one located on 3B, one on 6A and two SNPs located on chromosome 6B. The LOD scores ranged from 3.05 to 7.92. One stable SNP across two environments located on chromosome 5A associated with FLL with LOD value ranging from 3.88 to 6.55 (Table 2). Comparing the results across different methods, we found 36 common SNPs were co-detected simultaneously by at least two approaches (Fig. 3c, Table 1). Among these, four significant SNPs (RAC875_c8121_1490, Ku_27771_508, Tdurum_contig42962_2138, BS00022127_51) were detected by all four methods (Table 1).

**Table 2:** Stable SNPs co-detected in multiple environments associated with plant height and flag leaf length.

Trait	Years	SNP	Chr	Position (bp)	LOD > 3	r^2^ (%)
PH	2016, 2017	Kukri_c34553_89	2B	887912	4.60–6.43	1.95–2.32
	2016, 2017	RAC875_c8121_1490	3A	1272572	5.05–7.07	5.76–13.88
	2015, 2017, 2018	IACX3190	3B	1429597	5.05–6.35	4.29–8.51
	2017, 2018	GENE_3659_104	6A	2991495	3.44–3.86	1.50–2.54
	2016, 2017	wsnp_Ex_rep_c66315_64480362	6B	3350804	3.67–6.78	3.10–4.63
	2016, 2017, 2018	Ku_c5191_340	6B	3397322	3.05–7.92	0.72–4.39
FLL	2017, 2018	tplb0049a09_1302	5A	2453837	3.88–7.17	5.04–13.14

PH (plant height); FLL (flag leaf length). r^2^ (%) represents proportion of total phenotypic variation explained by each SNP.

We further checked the co-detected common SNPs simultaneously in multiple environments and different methods and screened five most stable SNPs (Kukri_c34553_89, RAC875_c8121_1490, wsnp_Ex_rep_c66315_64480362, Ku_c5191_340, and tplb0049a09_1302). Among these, one SNP was associated with FLL and the rest of four were identified for PH across multiple environments and methods (Fig. 3c, Table 3). Finally, we extended our screening criteria for significant QTLs and detected several major QTLs with phenotypic variation explained ranged from 5.5 to 13.8% associated with all the studied traits (Supplementary Table S3). Comparatively, the four ML-GWAS models (FASTmrMLM, FASTmrEMMA, mrMLM, and pLARmEB) to uncover genomic regions associated with plant height and related traits, the pLARmEB model detected the most SNPs (49), most of which associated with PH (36 SNPs), while, FASTmrEMMA identified the least SNPs (32; Fig. 4b).

**Table 3:** Stable SNPs identified simultaneously in different environments and different methods associated with plant height and flag leaf length.

Trait	Years	Methods (1–4)	SNP	Chr	Position (bp)	LOD > 3	r^2^ (%)
PH	2016, 2017	2016 (1, 4), 2017 (1, 2)	Kukri_c34553_89	2B	887912	3.22–6.43	1.95–3.42
	2016, 2017	2016 (1, 2, 3, 4), 2017 (1, 2, 3)	RAC875_c8121_1490	3A	1272572	4.98–9.25	5.67–13.88
	2016, 2017	2016 (1, 4)	wsnp_Ex_rep_c66315_64480362	6B	3350804	3.12–6.78	3.10–5.73
	2016, 2017, 2018	2016 (1, 2, 4), 2017 (1, 4)	Ku_c5191_340	6B	3397322	3.05–9.30	0.72–12.99
FLL	2017, 2018	2017 (1, 2)	tplb0049a09_1302	5A	2453837	3.88–7.17	5.04–13.14

Methods (1–4): Corresponding four multi-locus GWAS methods i.e. mrMLM, FASTmrMLM, FASTmrEMMA, and pLARmEB, respectively. r^2^ (%) represents proportion of total phenotypic variation explained by each SNP. PH (plant height); FLL (flag leaf length).

### GWAS using three single-locus models

Three single-locus GWAS (SL-GWAS) methods i.e. FarmCPU, MLM, and MLMM were used to further analyzed the results of the same plant architectural traits. A total of 97 significant SNPs were detected by the three SL-GWAS methods for the above mentioned traits across multiple environments (Fig. 3d, Supplementary Table S4). Among these SNPs, chromosome 5A harbored most of the SNPs (28) followed by 3B (14) and 3A (11). In the three SL-GWAS methods, FarmCPU detected 56 significant SNPs, MLM detected 19, and MLMM detected 22 significant SNPs associated with different traits in multiple environments (Fig. 3d, Supplementary Table S4). We further checked the common SNPs detected by all three SL-GWAS methods across multiple environments and methods and found three most stable SNPs (RAC875_c8121_1490, BS00049008_51, and tplb0049a09_1302) were repeated consistently by all methods in most of the environments (Supplementary Table S4). We further extended the screening criteria for significant SNPs and detected a total of 19 SNPs co-detected by using ML-GWAS and SL-GWAS methods together (Supplementary Table S5). This practice adds an extra screening to the GWAS approaches and thus makes us more confident about the results. By comparing the results of all three SL-GWAS methods, the FarmCPU model identified the most SNPs (56), while MLM detected the least SNPs (19). Manhattan and Q-Q plots of the above three single-locus GWAS models for plant architectural traits are presented in Supplementary Fig. S4.

### Traits having common associations

SNPs associated with more than one trait are very useful for marker-assisted selection. A total of five SNPs (Jagger_c6772_80, RAC875_c8121_1490, BS00089954_51, Excalibur_01167_1207, and Ku_c5191_340) were detected associated with more than one trait across multiple environments (Supplementary Table S6). Among these, one SNP (Excalibur_01167_1207) on chromosome 5A associated with PH and FLW. The rest of four pleiotropic SNPs were associated with PH and FLL across multiple environments. The presence of pleiotropic effects of these SNPs controlling plant height and flag leaf length were confirmed by the correlation analysis (Fig. 2). These pleiotropic SNPs (Jagger_c6772_80, RAC875_c8121_1490, BS00089954_51, and Ku_c5191_340) were located on chromosome 1A, 3A, 3B, and 6B, respectively. Moreover, these pleiotropic associations suggest that the aforementioned SNPs have multifaceted role in plant architectural traits and highlight the significance of flag leaf length and width to plant height.

### Candidate genes identification

**Figure 5:**
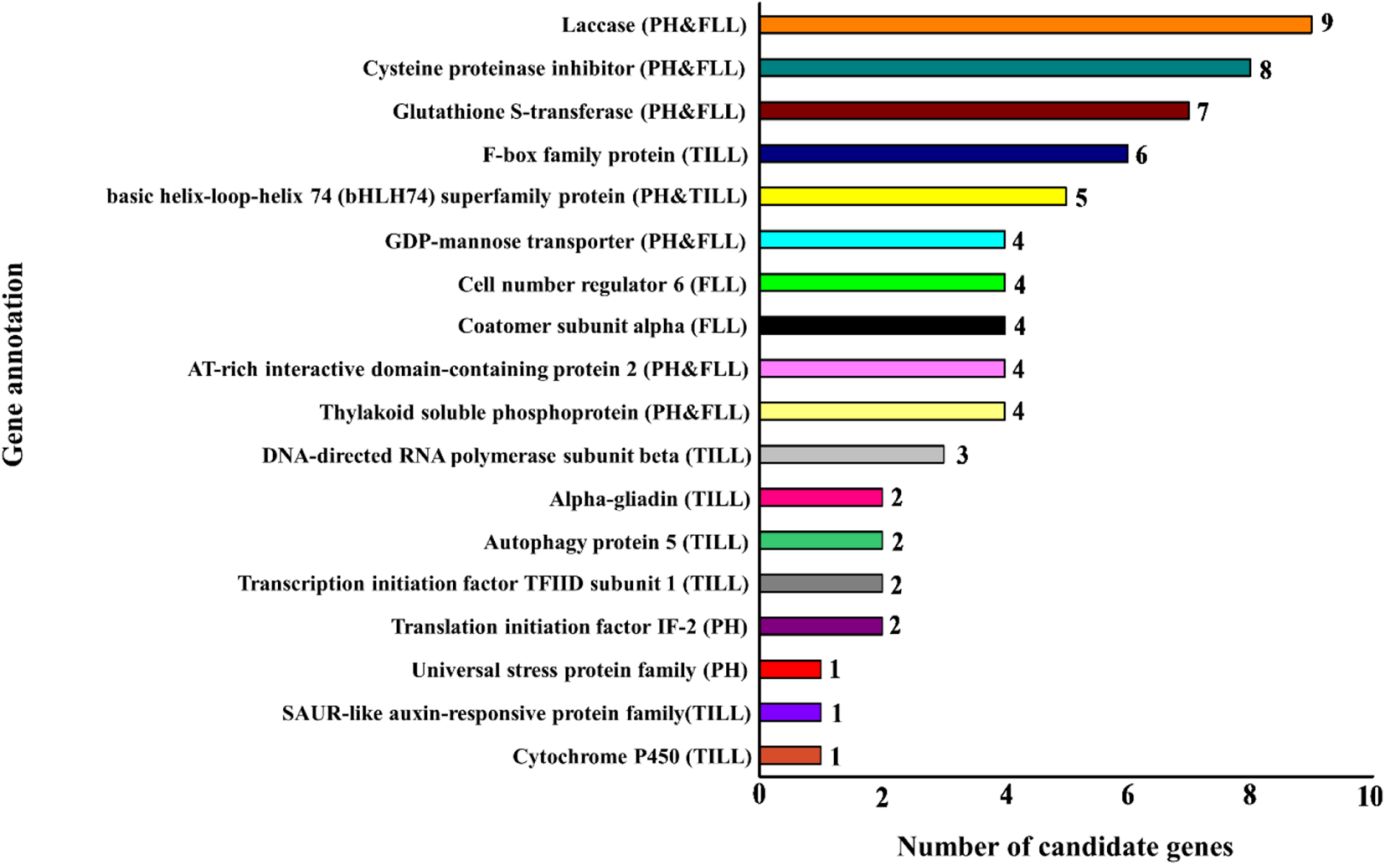
Putative candidate genes responsible for important functions associated with plant architectural traits. PH (Plant height); FLL (flag leaf length); TILL (number of tillers per plant).

To further understand the genetic basis of plant architectural traits, we predicted a total of 152 candidate genes that were surrounding the peak SNPs. Interestingly, several major candidate genes that were directly associated with the consensus SNPs had exact same annotations (Fig. 5, Supplementary Table S7). For instance, several putative candidate genes for PH and FLL, annotated as Laccase which is used for lignin polymerization to help in a variety of functions in plant development^28^. Similarly, the putative genes responsible for PH and FLL annotated as Cysteine proteinase inhibitor, has a function in plant growth and defense^29^. We also found a significant hit for auxin related gene which regulates cell and organ growth in rice^30^, and plays a prominent role in shoot apical meristem growth^31^. Three putative candidate genes, *TraesCS6A01G142000*, *TraesCS5A01G533200*, and *TraesCS5A01G533300* were revealed homology to the transcription factor basic helix-loop-helix 74 (*bHLH74*) which was reported to be involved in cell elongation and plant development^32–34^. Another gene (*TraesCS6A01G174700*) corresponds to Cytochrome P450, which is a part of ent-kaurenoic acid oxidase, an enzyme of the gibberellin acid (GA) metabolism^35^. Additionally, six putative candidate genes surrounding significant SNPs associated with number of tillers have annotations as F-box family protein, involved in plant vegetative and reproductive growth^36^. Further examples are given in Fig. 5 and Supplementary Table S7. Despite these results, further research is required to validate the possibility of these candidate genes with the architectural traits, these results will provide useful information for designing functional markers and for future work.

## Discussion

In this study, we employed four multi-locus GWAS models and three single-locus models to identify SNPs that significantly associated with plant height, flag leaf length, flag leaf width, and number of tillers across environments in hexaploid wheat. Plant height is a key factor in crop breading as it plays a crucial role in reshaping plant architecture and affects lodging and grain traits^16,37^. The real success of green revolution was the use of semi dwarf wheat cultivars. We identified some significant SNPs across multiple years by different ML-GWAS approaches (Tables 1, 2, 3, Fig. 4a–e). A total of 24 SNPs was consistently detected by most of the ML-GWAS methods for plant height (Table 1 and Fig. 3c). Most of them located on chromosomes 5A and 7A, consistent with some SNPs reported in previous studies^38–40^. Chromosome 5A was revealed to harbor the highest number of significant SNPs for plant architectural traits as showed in Table 1 in this study and has been confirmed of having the most useful and reproducible regions in wheat genome^41–44^. Sukumaran et al.^45^ reported the most numbers of significant SNPs for yield traits on chromosomes 5A and 6A in a spring wheat population. Similarly, the SNPs detected on chromosomes 5A and 6A are most likely the MTAs reported previously^42,46^.

Five SNPs were detected across both multiple environments and methods for plant height and flag leaf length, of which two were located on chromosome 6B (Table 3). These results revealed the significance of chromosome 6B on plant height, consistent with the findings of some previous studies^47–49^. Among the five stable SNPs, one (Kukri_c34553_89) was located on chromosome 2B with LOD ranging from 3.2–6.4, which was also detected as one of the environment-stable SNPs and was revealed the positive effects on harvest index^50^. Two consensus SNPs (RAC875_c8121_1490 on chromosome 3A and Ku_c5191_340 on chromosome 6B), were also reported for plant height across different wheat populations^51^. The stable SNP (tplb0049a09_1302) located on chromosome 5A, was also reported by Ain et al.^38^, they used 90K array to identify several genomic regions associated with yield related traits in historical wheat genotypes of Pakistan. Another SNP (BobWhite_c5694_1201) located on chromosome 4B is likely same to the QTL identified in a spring wheat population by Zou et al.^52^.

In addition to the stable SNPs, if the other SNPs in this study were considered, a wider co-localization was found between the SNPs in this study and in previous studies. For example, the SNP, D_contig10675_778 (located in chromosome 2D at 12.3 cM) was found to be co-localized with the QPht/Sl.cau-2D.1 (BobWhite_rep_c63957_1472 located in chromosome 2D at 12.24 cM) reported in a previous study^53^, both at the physical location 20.8 MB with a dwarf gene (Rht8-19.6 Mb). The cultivars that harbors the reduced height gene Rht8, short stature tended to get more spike number in unit area^9^. In addition, the Rht8 has the ability to improve the early vigor of semi-dwarf wheat^53^. Thus, the SNP D_contig10675_778 identified in this study is of interest for further genetic studies and molecular breeding.

The SNPs BS00023152_51 and tplb0049a09_1302 (located on chromosome 5A) detected in two environments in this study falls in the region of the SNP (AX_110446653, 671.2 Mb) reported by a previous study^1^. It should be mentioned, except the Rht8, the SNPs in this study were not well co-mapped with some semi-dwarf genes such as the Rht1, 2, 14, 16 and 18. Similarly, in a previous study, where a total of 14 SNPs for plant height was mapped on chromosomes 1A, 1B, 2A, 3A, 3B, 4D, 5A, 5B, 6B and 7A, however, only the AX_108916749 on chromosome 4D is at the same position as Rht-D1^54^. The reasons might be (i) that a relatively small population from a limited region was used in studies, (ii) that the 90K array in which the lower density of makers is not reliable for detected the SNPs with small effects and for the comparison of the SNPs across different studies and (iii) the inconsistency of SNPs with previous studies possibly indicated the identification of potentially new genes.

Flag leaves are the primary source of carbohydrate production to sustain proper crop growth and development, thus the importance of flag leaf morphology on increasing grain yield has widely been studied^7,8^. In present study, we detected several consensus SNPs associated with flag leaf length and width. For flag leaf length (FLL), three consensus SNPs were detected on chromosomes 4B, 5A, and 6D (Table 1). Bilgrami et al. ^11^ reported a total of 47 significant SNPs associated with number of tillers in breed wheat. The SNP (BS00021881_51) associated with FLL simultaneously detected via two ML-GWAS approaches i.e. FASTmrMLM and pLARmEB was reported earlier in QTL mapping^55^. Number of tillers have been considered the primary trait for increasing cereal yield no matter in favorable or unfavorable environments^11^. In the present study, we highlighted several prominent SNPs controlling number of tillers. Two stable SNPs (BS00022127_51 and wsnp_BE499835B_Ta_2_5) associated with the number of tillers per plant corresponded to the previously reported SNPs in wheat^56,57^. Among the stably detected SNPs for number of tillers, BS00022127_51 located on chromosome 7B, consistently detected by all four ML-GWAS methods (Table 1). Bilgrami et al. ^11^ reported a total of 47 significant SNPs associated with number of tillers in breed wheat. These SNPs might be the best target for improving the ability of light harvesting and the tiller number of plants. The comprehensive understanding of leaf morphology will provide new insights to the genetic mechanism of crop growth and development.

By further reviewing the significant SNPs results, 174 and 97 SNPs were detected by ML-GWAS and SL-GWAS models, respectively, these results signify the importance of ML-GWAS over SL-GWAS approaches. In earlier studies, mostly SL-GWAS methods were adopted, but only few SNPs for each trait have been identified due to its procedural limitations^58^. According to our results of SL-GWAS models, MLM model detected the least SNPs (19), which reveal the setting of very high threshold, due to which many small-effect loci are missed^59^. To make up for the limitations of these methods, some multi-locus approaches such as FASTmrMLM^60^, FASTmr EMMA^61^, mrMLM^59^, and pLARmEB^62^ have been used in this study. These models can improve the accuracy of SNPs with high detection power and less stringent criteria, and no Bonferroni multiple test correction is needed^59,61^. In our results, the number of significant SNPs by ML-GWAS were comparatively higher than SL-GWAS models, which suggest the significance of ML-GWAS models. Jaiswal et al.^63^ verified that ML-GWAS has more detection power than SL-GWAS by revealing ten MTAs through SL-GWAS while, 22 MTAs through multi locus mixed model (MLMM) and 58 MTAs through multi-trait mixed model (MTMM). Furthermore, we detected a total of 19 SNPs co-detected by using ML-GWAS and SL-GWAS methods together (Supplementary Table S5), which reveal the credibility of these SNPs as highlighted by several approaches. Zhu et al.^64^ suggested the combination of both SL-GWAS and ML-GWAS methods, which contributes efficiently to the detection of significant loci associated with pre-harvest sprouting tolerance in wheat. According to Li et al.^65^, the power of QTN detection in association analysis can be improved by combining single locus and multi-locus GWASs. Through integrating the results of ML-GWAS and SL-GAWS methods led to the verification of the significance of ML-GWAS models. However, some recent findings revealed the reliability of association studies can be improved by combining single-locus and multi-locus GWAS approaches^65–68^.

Taken together, four multi-locus and three single-locus GWAS models were used for parsing the genetic background of plant architectural traits (PH, FLL, FLW, and TILL) in hexaploid wheat. A total of 271 significant SNP was detected across multiple environments and in different methods. Comparatively, 174 and 97 significant association signals were detected by ML-GWAS and SL-GWAS models, respectively which signifies the importance of ML-GWAS over SL-GWAS approaches. By further appraising these GWAS methods, the pLARmEB and FarmCPU models outperformed in SNP detection compared to the other ML-GWAS and SL-GWAS methods, respectively. Taken together, the results of ML-GWAS, revealed five most stable SNPs i.e. Kukri_c34553_89, RAC875_c8121_1490, wsnp_Ex_rep_c66315_64480362, Ku_c5191_340, and tplb0049a09_1302 which were consistently detected across multiple environments and methods.

Our study will provide new insights to the genetic basis of plant architectural traits and can serve as a basis for further functional investigation. The loci and significant SNP markers identified in this study can be used for pyramiding favorable alleles in developing varieties with desirable plant architecture and potentiality in the genetic improvement of grain yield. Among them, the stable SNPs identified across years in this study are of great importance. Secondly, based on the correlation between traits and the direction of SNP effects, we can design the combinations or find the accessions with a high percentage of favorable alleles. For example, the plant height and flag leaf length generally positive correlated with each other, but both have less correlation with flag leaf width and tiller numbers. These information, together with the SNPs identified, will be beneficial for breeding design. However, to achieve these goal, the larger populations and higher density genetic maps are required. It is necessary (i) to narrow down the SNP confidence interval thus that the markers tightly linked to the genes of interest should be much reliable for marker-assisted selection and for fine mapping and subsequent cloning of the candidate genes, (ii) to estimate the effects of number of alleles for desirable phenotypic values for each traits as reported by some previous studies^1,69^ and to convert the SNPs of interest into kompetitive allele-specific PCR (KASP) markers, and to further verify in bi-parental populations and (iii) to jointly investigate the SNPs for the other agronomic important traits including grain yield and grain qualities across multiple genetic mapping populations thus to gain a comprehensive picture for breeding design.

## Materials and methods

### Plant materials and phenotyping

A total of 319 wheat germplasm accessions from the collection at the Hubei Academy of Agricultural Science in Hubei Province, China, which represent a wheat gene pool adapted to central China and the Yangzi River regions. The plant materials were grown in randomized complete blocks with three replicates at the experimental farm of Huazhong Agricultural University, Wuhan, China for four consecutive winter seasons (2015–2018). Twenty individuals from each variety (line) were grown in two rows with a distance of 15 cm between plants in each row and 20 cm between rows. Field management essentially followed normal local wheat cropping practices. The lines were harvested individually at maturity to prevent seed contamination among lines. Four phenotypic traits were evaluated, including plant height across four environments (2015–2018) and the rest of three traits i.e. flag leaf length, flag leaf width, and the number of tillers per plant across two environments (2017–2018). The measurements of these traits were performed by selecting five random individual plants in the middle of the row for each accession. Plant height was measured after physiological maturity by measuring the distance between the stem base and the top of the spike excluding awns. Flag leaf length was measured as the distance from the base to the tip of the leaf. Flag leaf width as the width of the widest section of the leaf. Number of tillers were recorded by counting the total number of fertile tillers per plant.

### Genotyping

A total of 319 wheat accessions were genotyped using the Illumina iSelect 90K SNP array ^70^ in the genotyping Laboratory of North Dakota State University in Fargo as described in our previously published study^27^. A quality preprocessing of genotyping data was done for sample call rate, SNP call rate, minor allele frequency (MAF) and Hardy–Weinberg equilibrium (HWE). This preprocessing was implemented in PLINK software (https://zzz.bwh.harvard.edu/plink/)^71^.

### Statistical analysis

Descriptive analysis, ANOVA, correlation analysis and heritability estimates were conducted in the R statistical package^72^. The broad sense heritability for the traits was estimated by the formula H^2^ = VG/(VG + VE) where VG and VE represent estimates of genetic and environmental variance, respectively^73^. Variance components for the studied traits were analyzed according to our previous study^27^, using general linear model to detect the effect of genotypes, environment, replication and genotype × environment interaction. All sources of variation were considered as random effects.

### Population structure and kinship analysis

The SNP markers and estimated methods for population structure and linkage disequilibrium (LD) were the same as in Muhammad et al.^27^. Population structure using a Bayesian cluster analysis was estimated by STRUCTURE 2.3.4 software^74^, and the obtained results were visualized with the STRUCTURE HARVESTER software^75^. A putative number of subpopulations ranging from *K* = 1 to 7 was assessed using 100,000 burn-in iterations followed by 500,000 recorded Markov-Chain iterations. To estimate the sampling variance (robustness) of inferred population structure, 10 independent runs were carried out for each *K*. *K* was estimated using an *ad-hoc* statistic ∆*K* based on the rate of change in log probability of data between successive values^26^. Principle component analysis (PCA) was calculated by R software for evaluating the population structure and compared to the result of STRUCTURE^9^. LD among markers was calculated using observed vs. expected allele frequencies of the markers in TASSEL v.5.0^38^.

### Genome-wide association studies

In this study, we used mrMLM software for four ML-GWAS (FASTmrMLM, FASTmrEMMA, mrMLM, and pLARmEB) and three SL-GWAS (FarmCPU, MLM, and MLMM) implemented by Genomic Association and Prediction Integrated Tool (GAPIT) in R^76^. Previously, SL-GWAS methods were mostly applied such as GLM and MLM. However, single-locus approaches have some limitations such as GLM leads to high false-positive rates (FPRs), while MLM utilizes Bonferroni corrections for loci detection to reduce the FPRs^77^. Though, this procedure is so stringent that results in missing significant SNPs^59^. Therefore, multi-locus GWAS approaches are the best alternatives. The stringent Bonferroni multiple test correction in the SL-GWAS analysis is substituted by a flexible selection criterion in multi-locus GWAS analysis, that reduces the possibility of missing out significant loci^59,61^. The four ML-GWAS methods were performed with default parameters, and the screening criteria for significance were set with LOD scores 3 or > 3^59,61,62^. However, for SL-GWAS models, the threshold for P-value was calculated based on the number of the markers (P = *1/n*, *n* = total SNP used) according to the method of^78^. Significant markers were visualized with a Manhattan plot using Haploview 4.2 software^79^. Important p-value distributions (expected vs. observed *p*-values on a – log^10^ scale) were shown with a quantile–quantile plot.

### Candidate gene analysis

Candidate gene sites were aligned and downloaded from the ViroBLAST database (https://urgi.versailles.inra.fr/blast/docs/aboutviroblast.html). The R Package Pathway Association Study Tool (PAST) version 1.0.1 was used to identify genes around the peak SNPs with a window size of 200 kb. To find candidate genes or putative related proteins of SNP flanking-regions, BLASTx search was conducted for significant marker-trait associations (MTAs) against recently released genome sequence IWGSC RefSeq v1.0^80^.

## Supplementary Information

Supplementary Information 1.

Supplementary Information 2.

Supplementary Information 3.
