# Supplementary material for: Uncovering genomic regions controlling plant architectural traits in hexaploid wheat using different GWAS models
Source: Sci Rep. 2021 Mar 24;11:6767. doi: 10.1038/s41598-021-86127-z (PMC7990932; doi:10.1038/s41598-021-86127-z)
Supplement: Supplementary file 1 — Supplementary Information 1. [file 41598_2021_86127_MOESM1_ESM.pdf]

Uncovering genomic regions controlling plant architectural traits in hexaploid wheat using different GWAS models

Ali Muhammad[1,2, 4], Jianguo Li[2], Weichen Hu[2], Jinsheng Yu[3], Shahid Ullah Khan[5], Muhammad Hafeez Ullah Khan[5], Guosheng Xie[2], Jibin Wang[1] and Lingqiang Wang[1,2,*]

[1]State Key Laboratory for Conservation and Utilization of Subtropical Agro-Bioresources, College of Agriculture, Guangxi University, 100 Daxue Rd., Nanning, Guangxi, China

[2]College of Plant Science and Technology & Biomass and Bioenergy Research Center, Huazhong Agricultural University, Wuhan 430070, China

[3]College of Agriculture and Food Science, Zhejiang A&F University, Lin'an 311300, China

[4]Department of Agriculture, Abdul Wali Khan University Mardan, Pakistan

[5]National Key Laboratory of Crop Genetic Improvement, Huazhong Agricultural University, Wuhan 430070, China

*****Correspondence: lqwang@gxu.edu.cn or lqwang@mail.hzau.edu.cn

Figure S1. Structure analysis of 319 wheat accessions based on unlinked SNP markers.

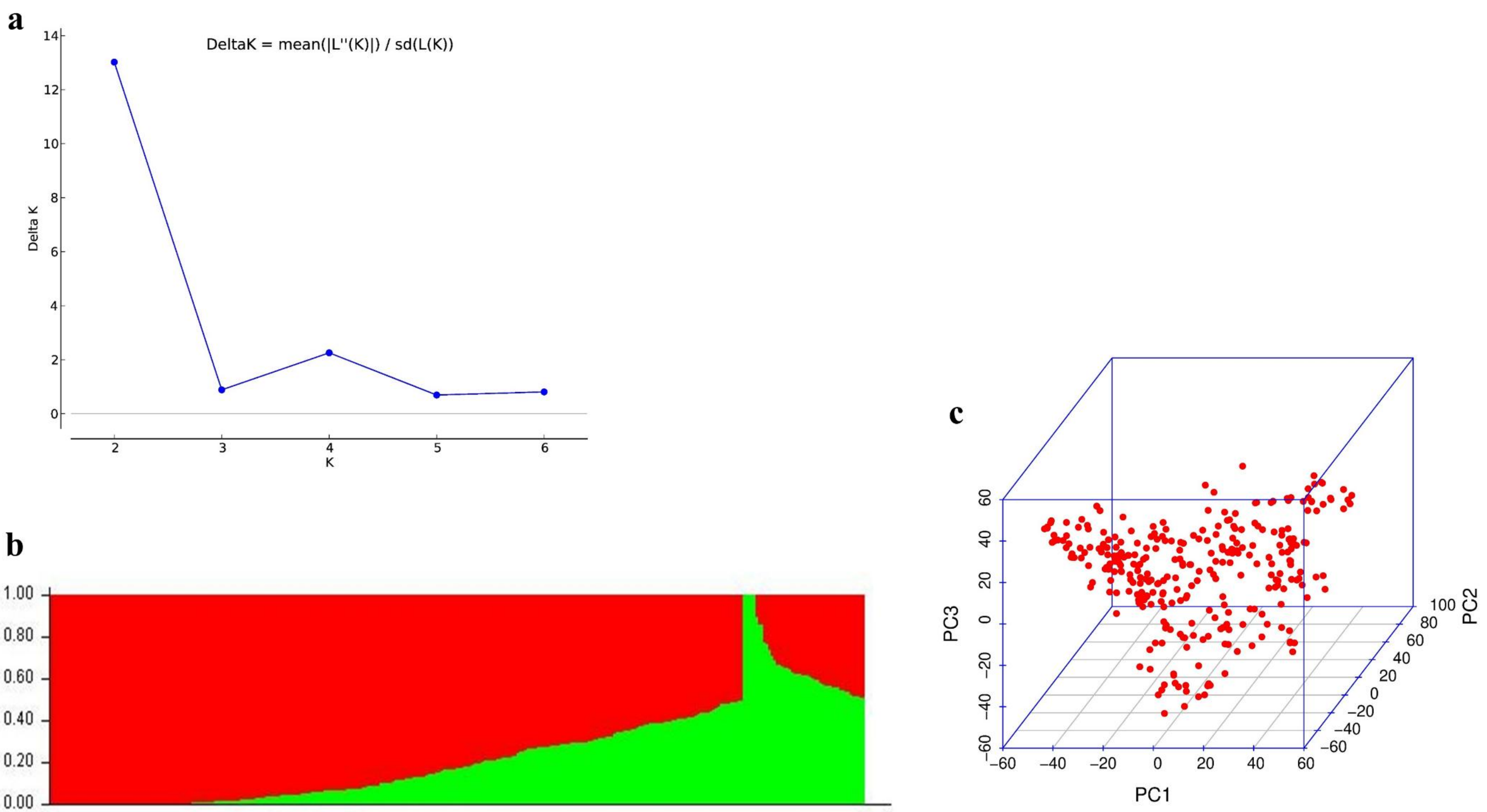

Figure S1. (a) ΔK Plot containing putative K ranging from 2 to 6. **(b)** Plot of subpopulations K=2, represented by two colors i.e. red and green which indicates the proportion of each subpopulation. **(c)** PCA based on standardized covariance of genotypic data.

Fig S2. Linkage disequilibrium (LD) analysis among markers.

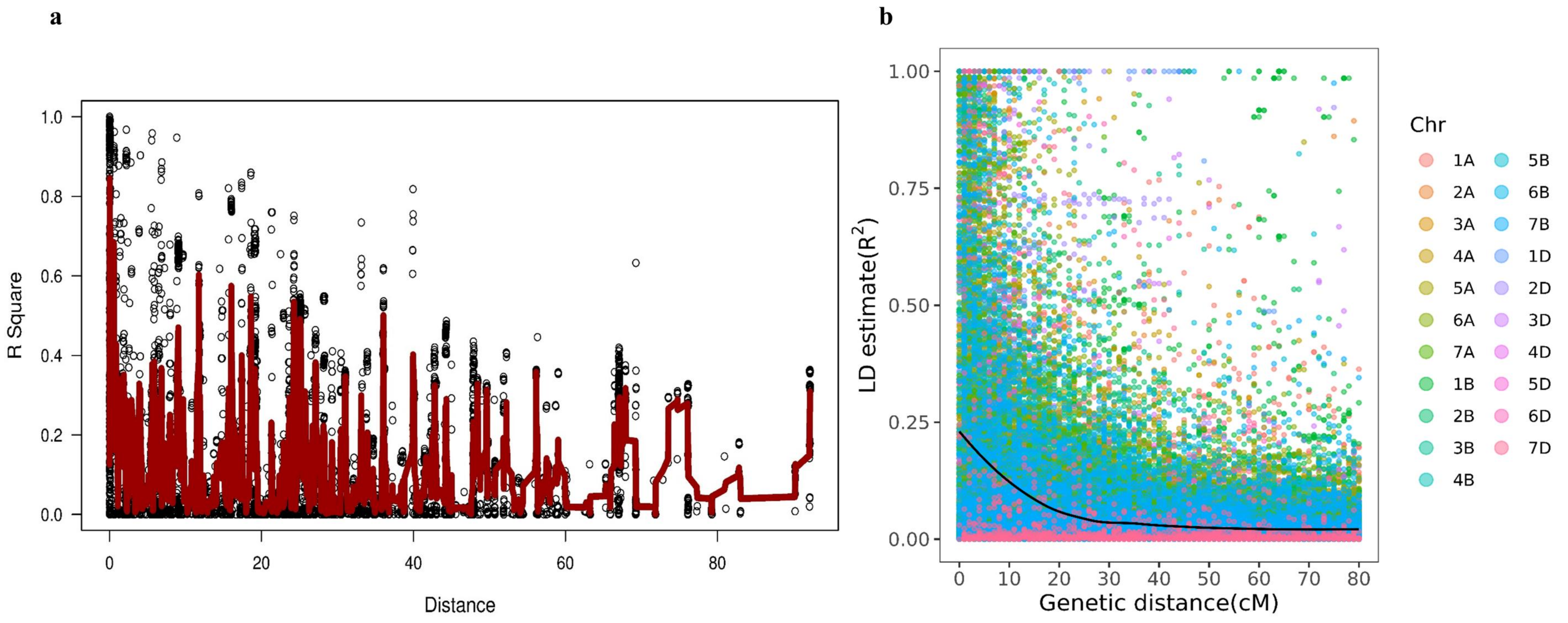

Figure S2. (a) LD decay for whole genome. **(b))** LD decay for A, B, and D sub-genomes.

Figure S3. Manhattan and quantile-quantile (Q-Q) plots of multi-locus GWAS models for plant height, flag leaf length, flag leaf width, and number of tillers across different environments.

PH2015

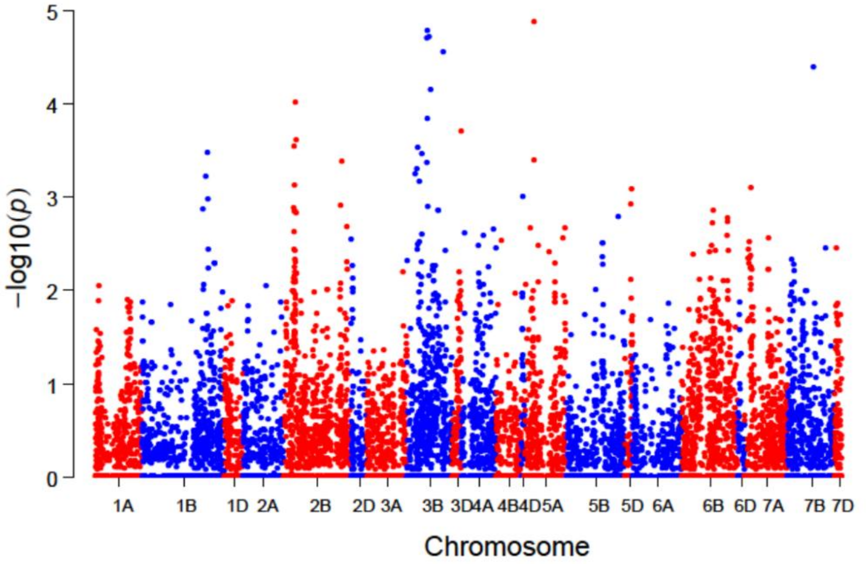

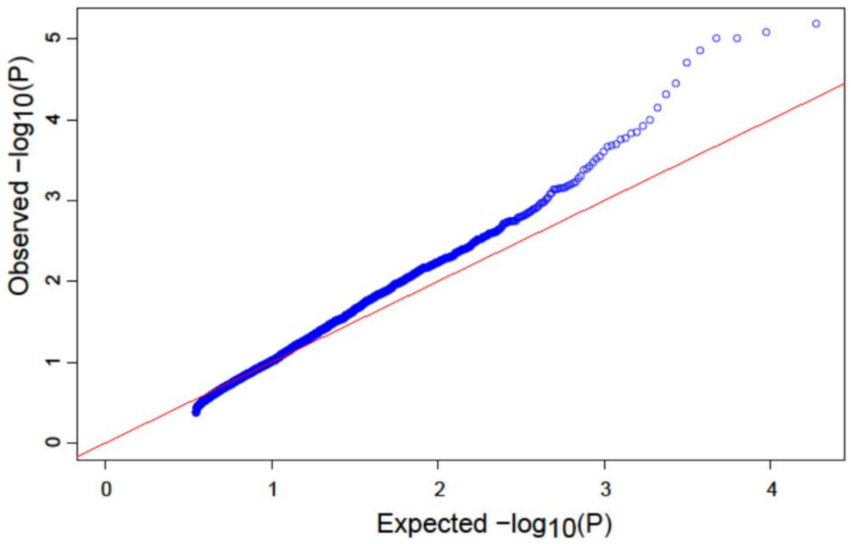

PH2016

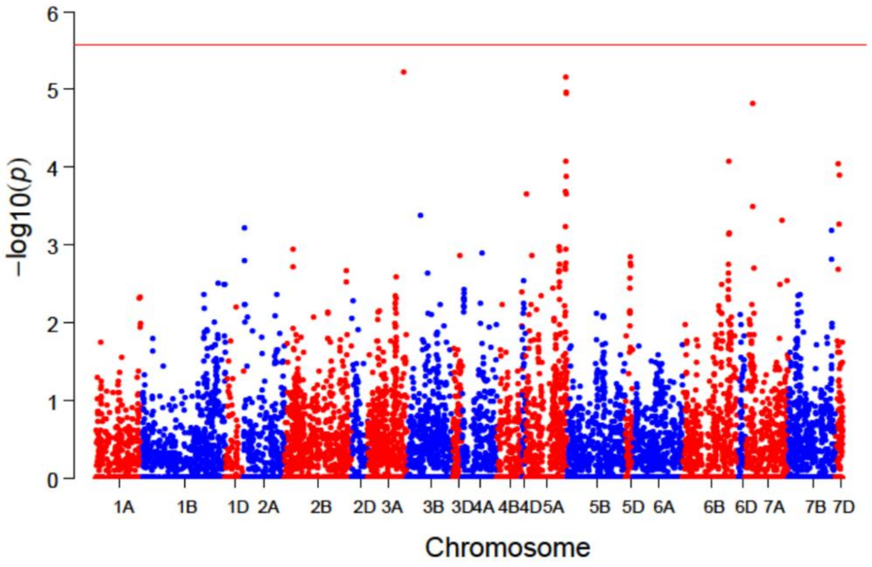

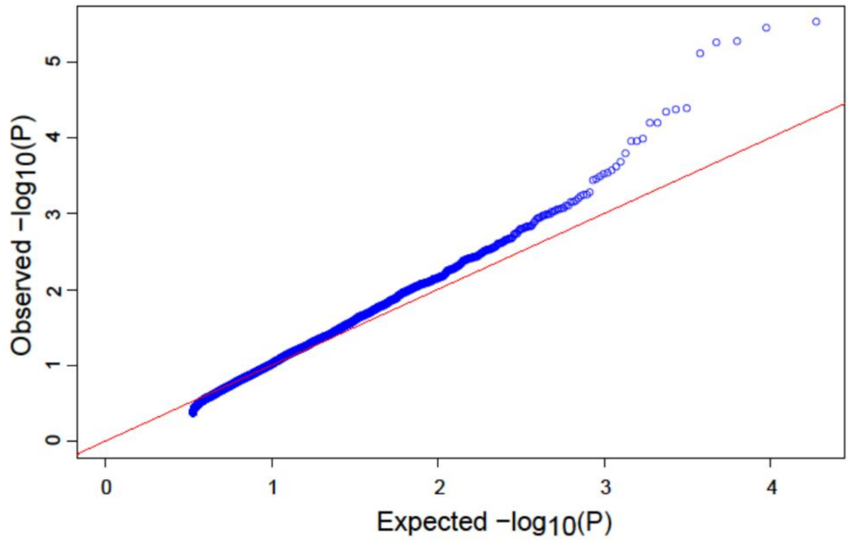

PH2017

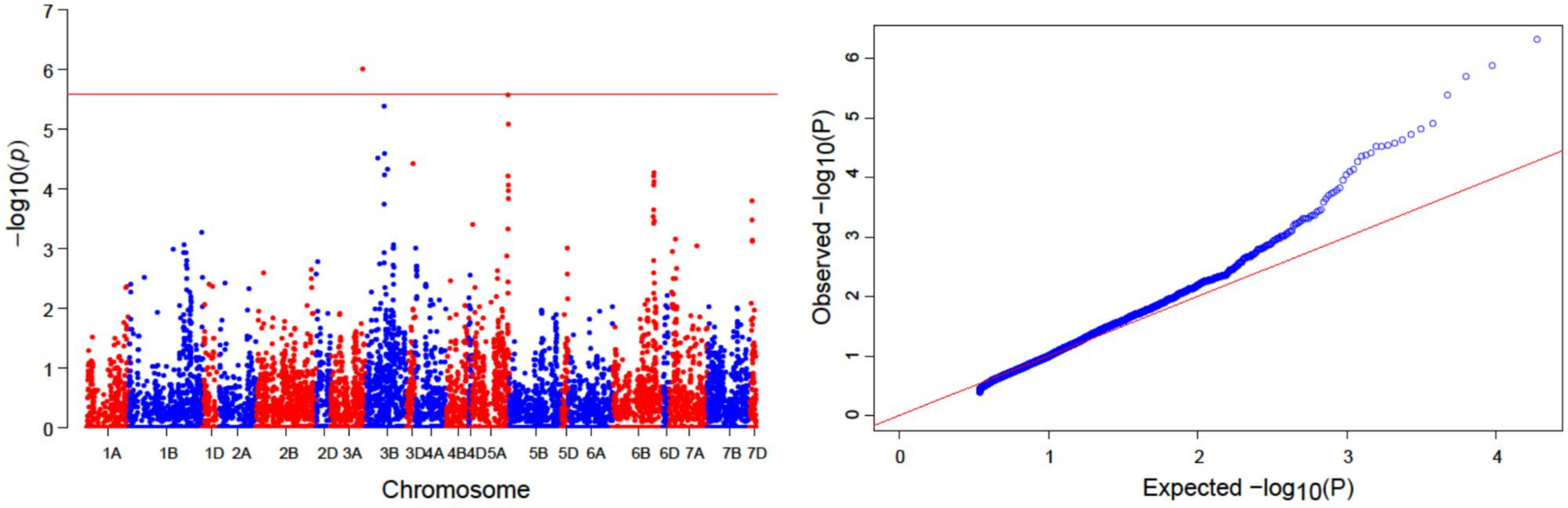

PH2018

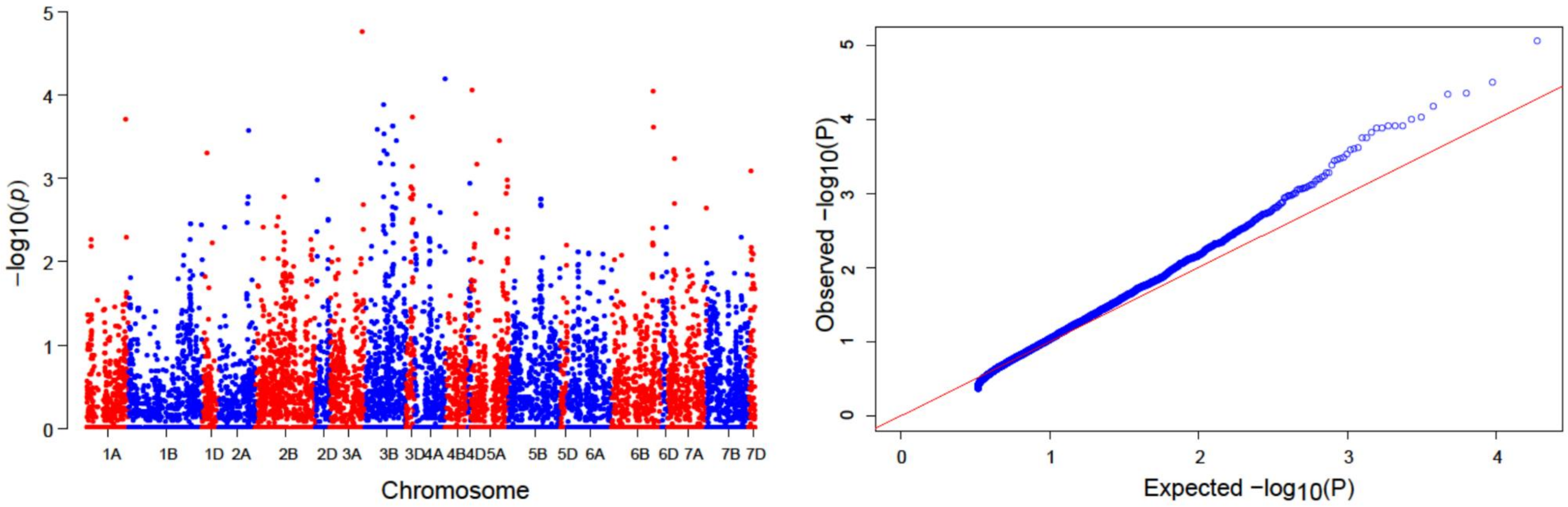

FLL2017

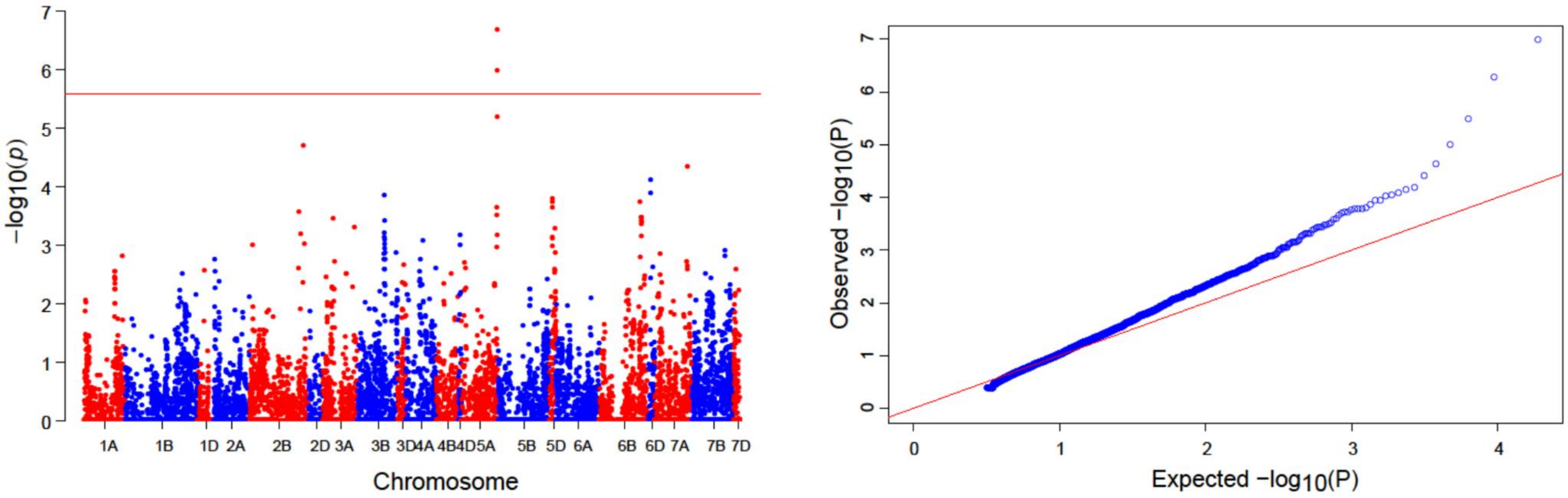

FLL2018

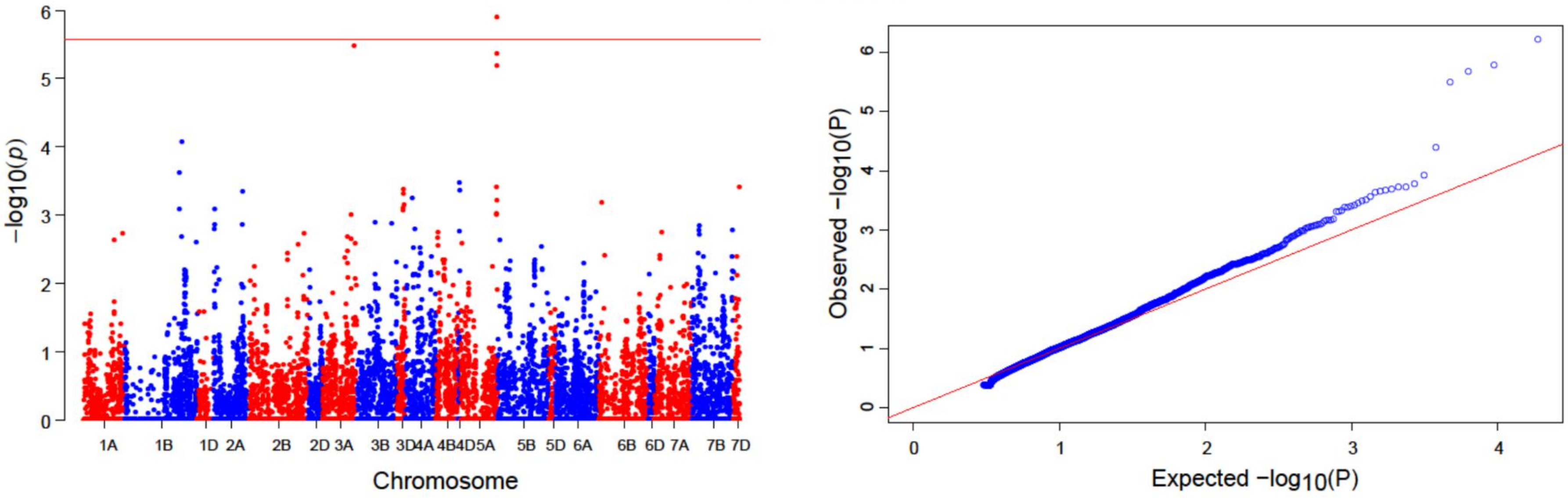

FLW2017

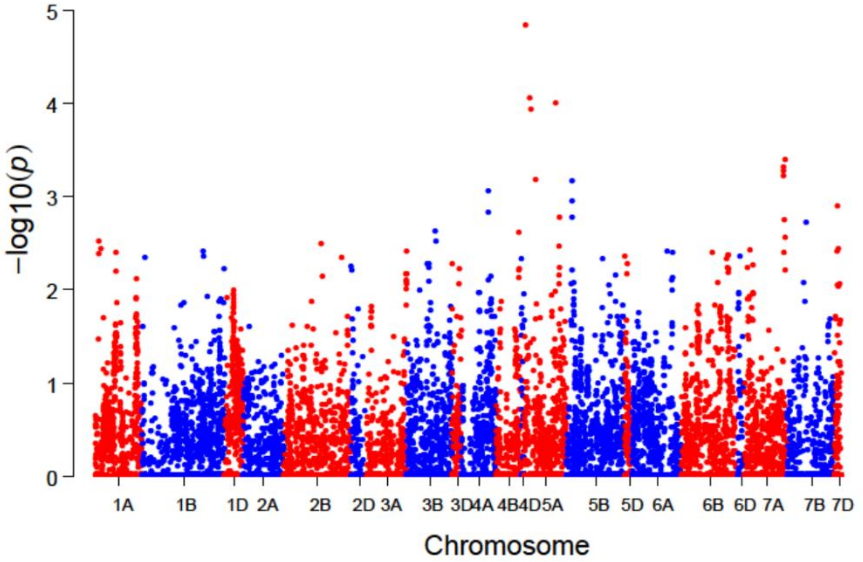

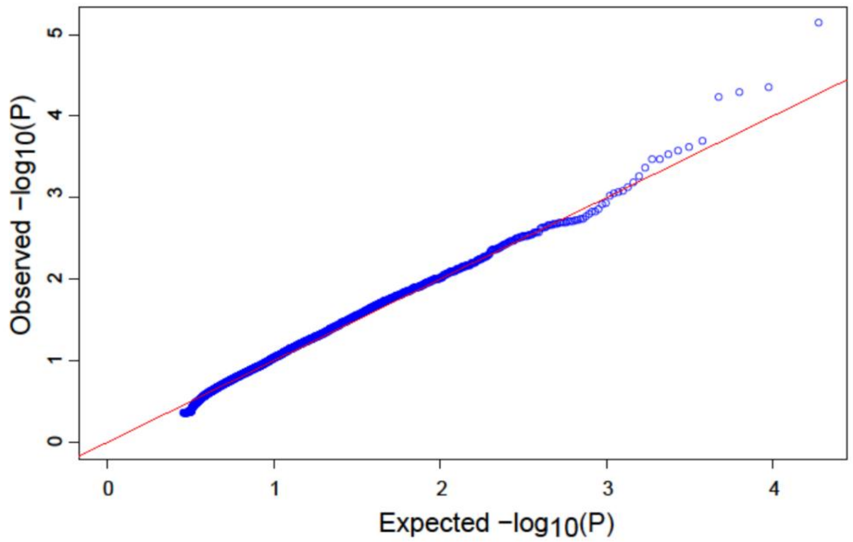

FLW2018

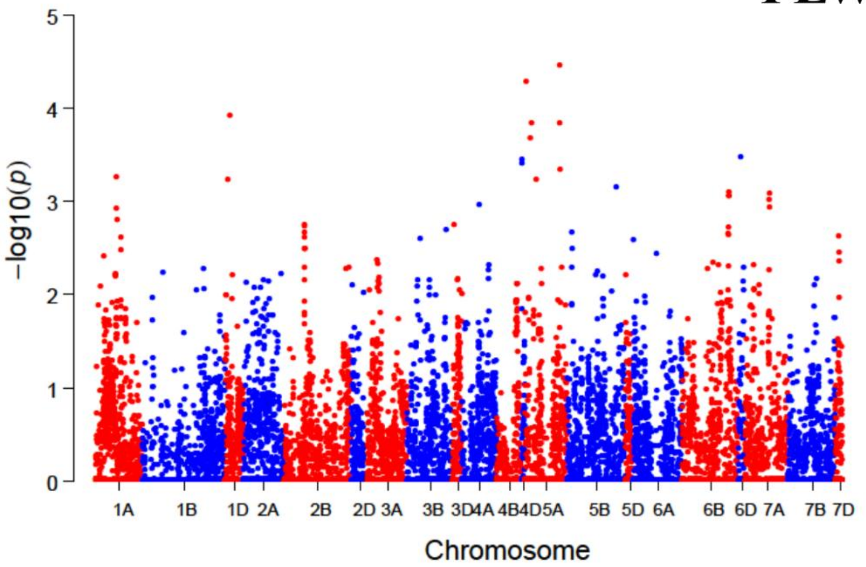

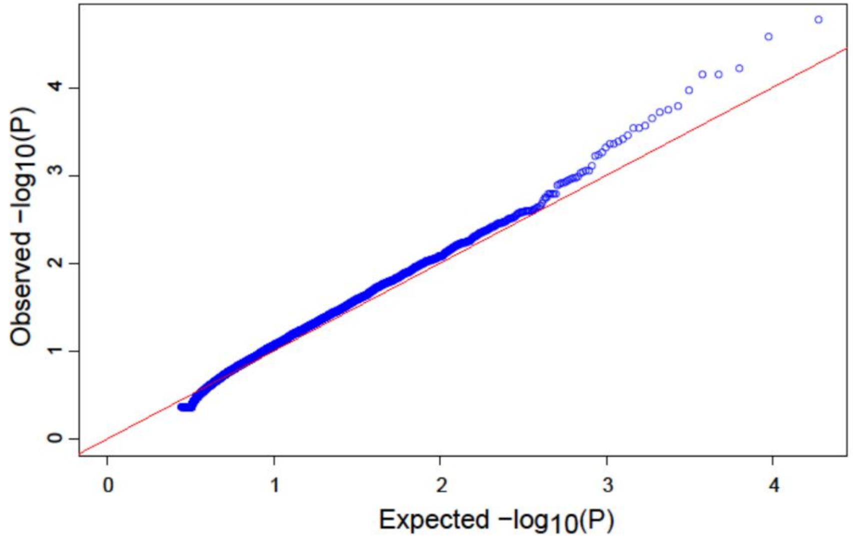

TILL2017

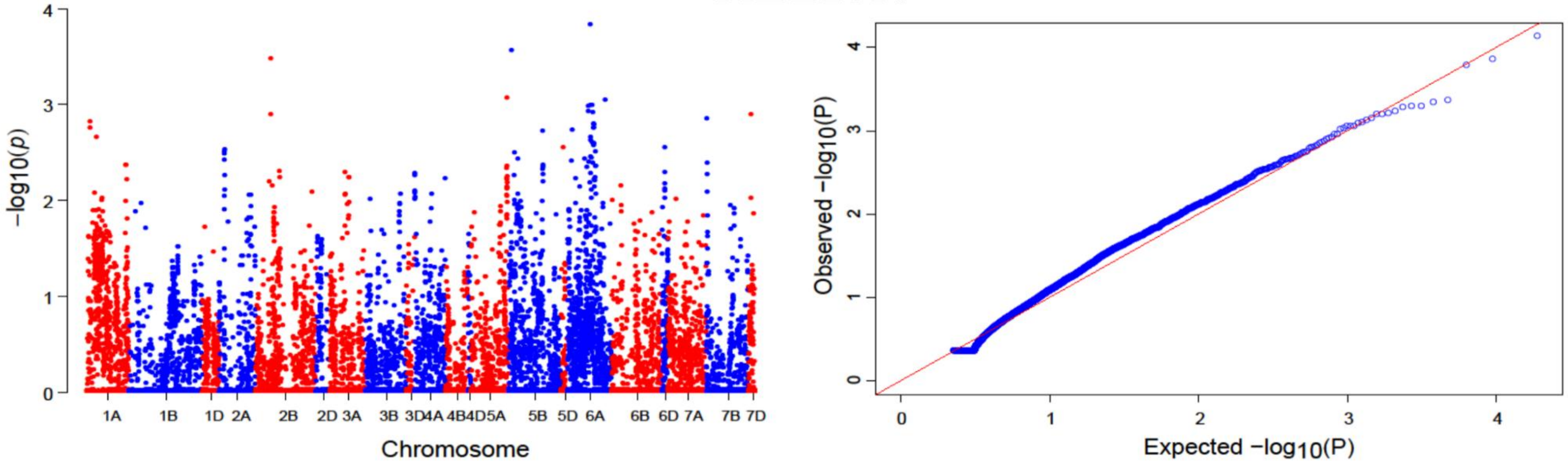

TILL2018

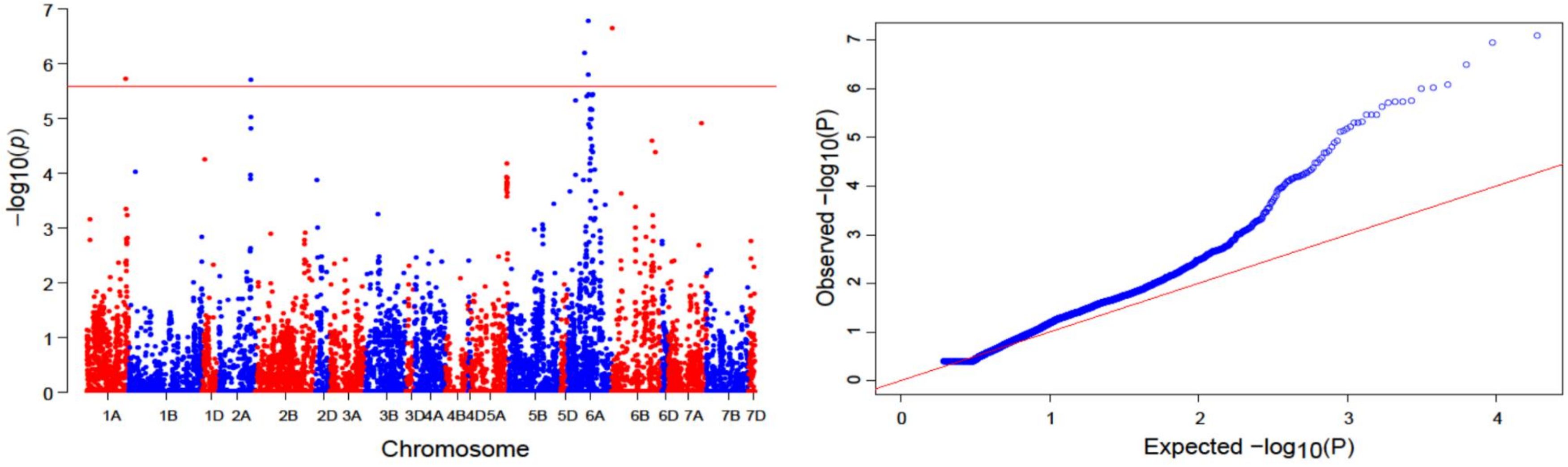

Figure S3a. Manhattan and Q-Q plots of FASTmrMLM model for plant height, flag leaf length, flag leaf width, and number of tillers across different environments.

PH2015

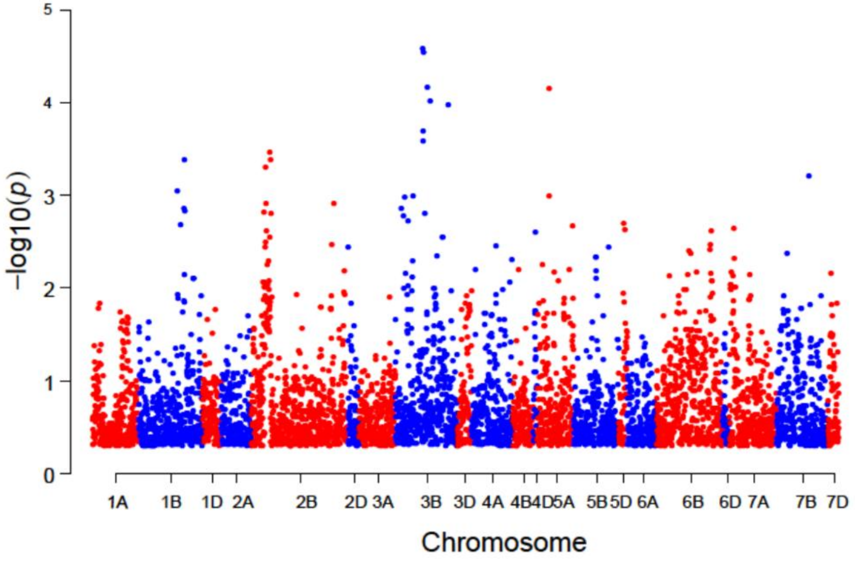

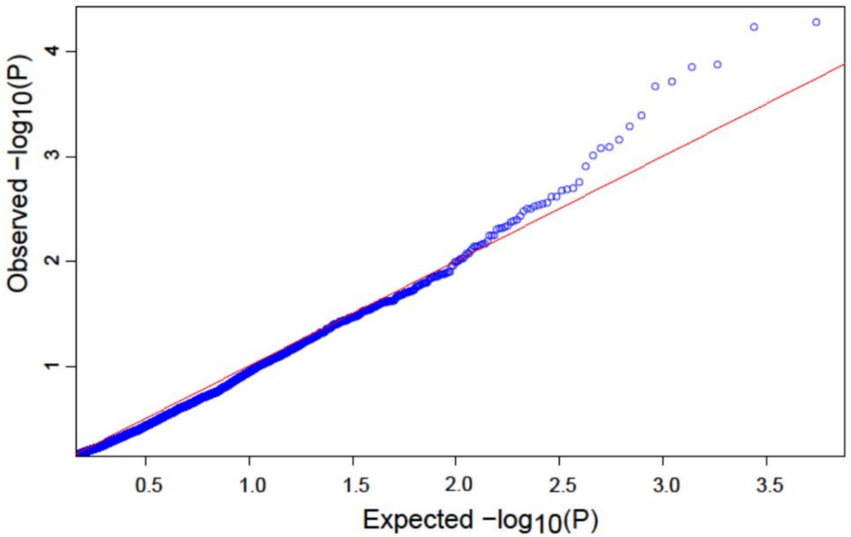

PH2016

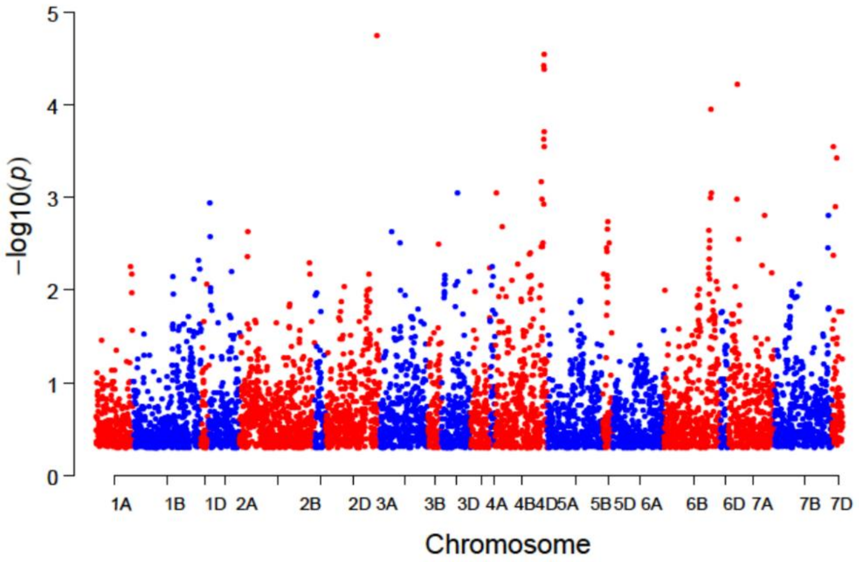

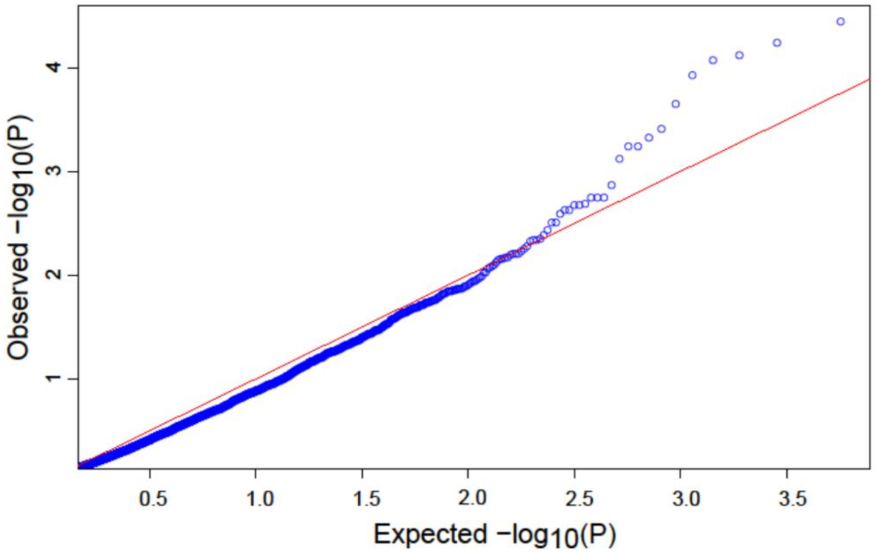

PH2017

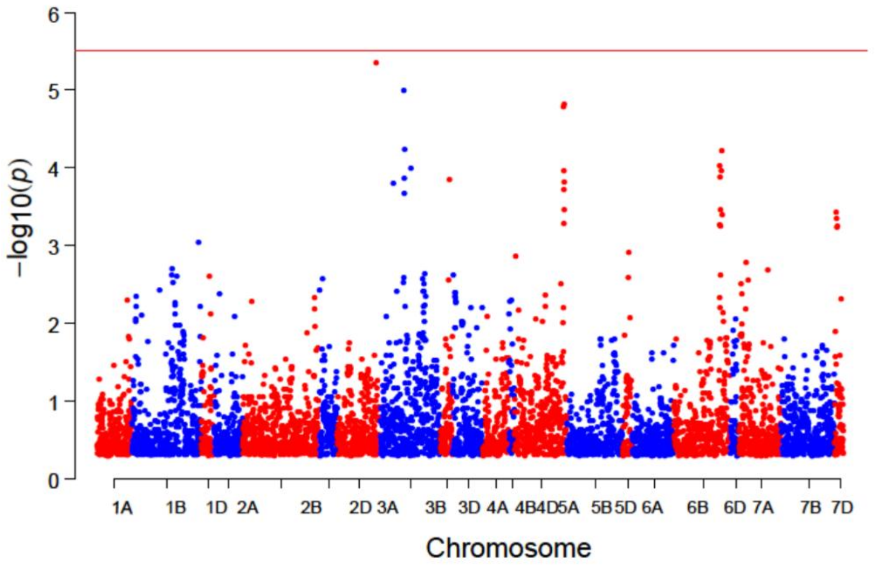

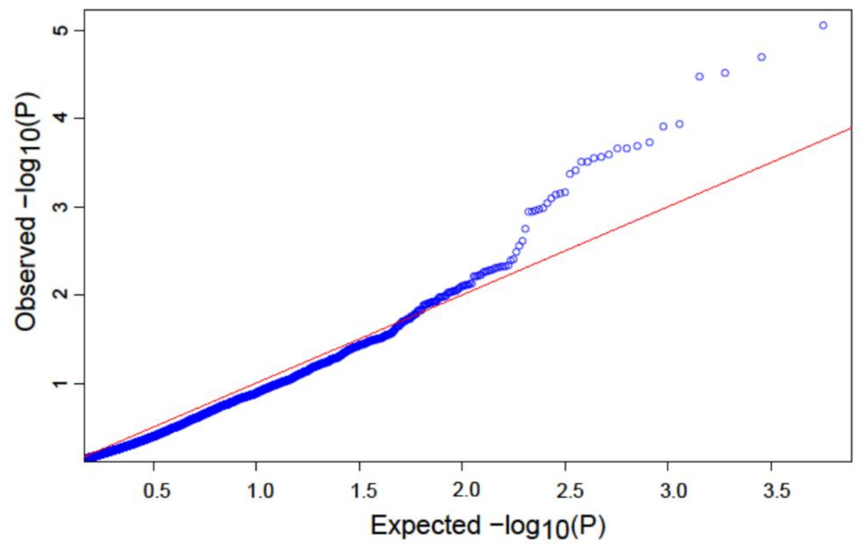

PH2018

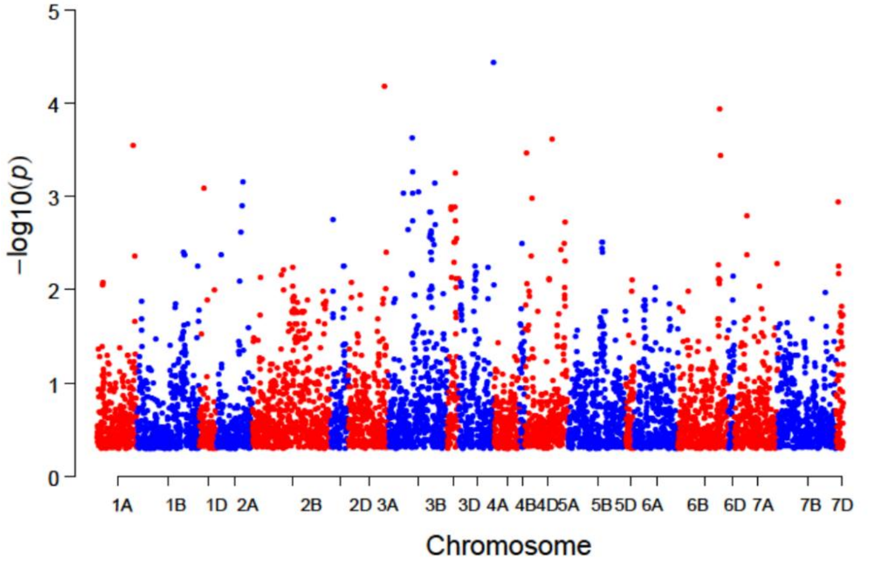

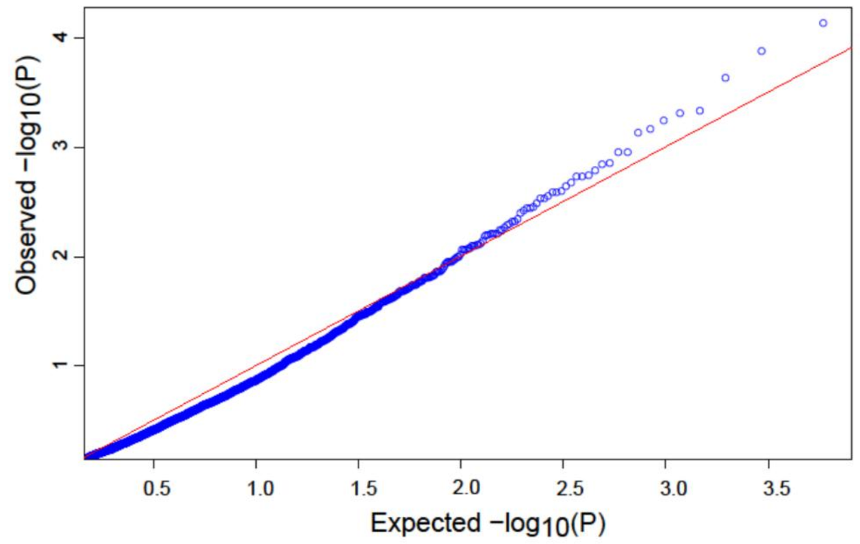

FLL2017

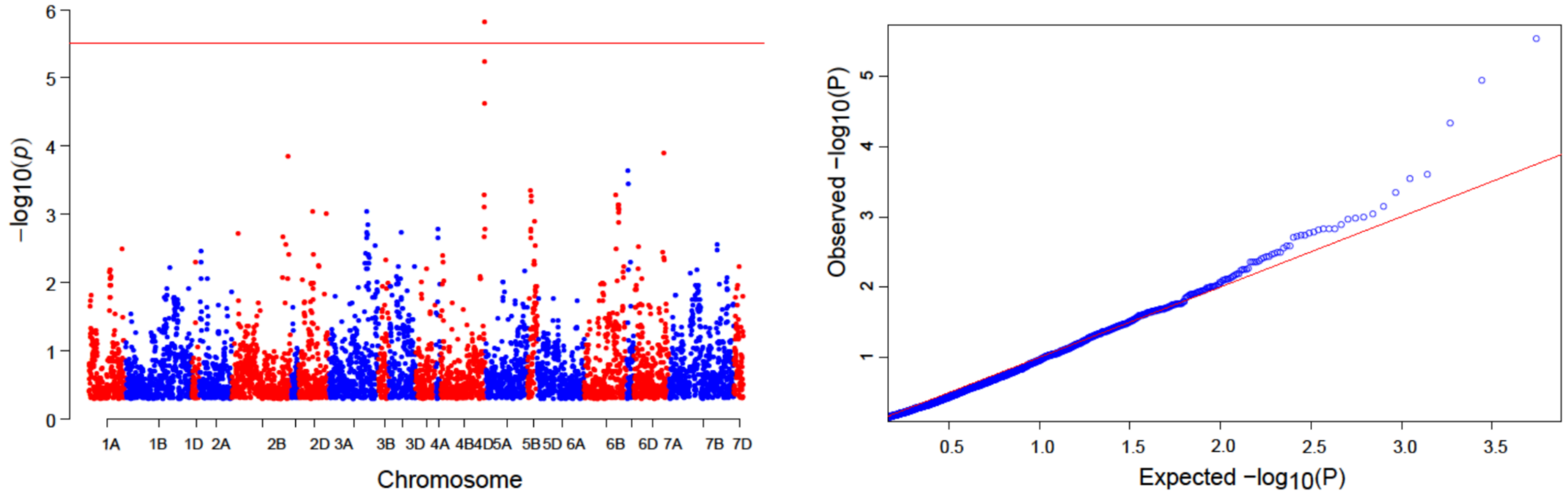

FLL2018

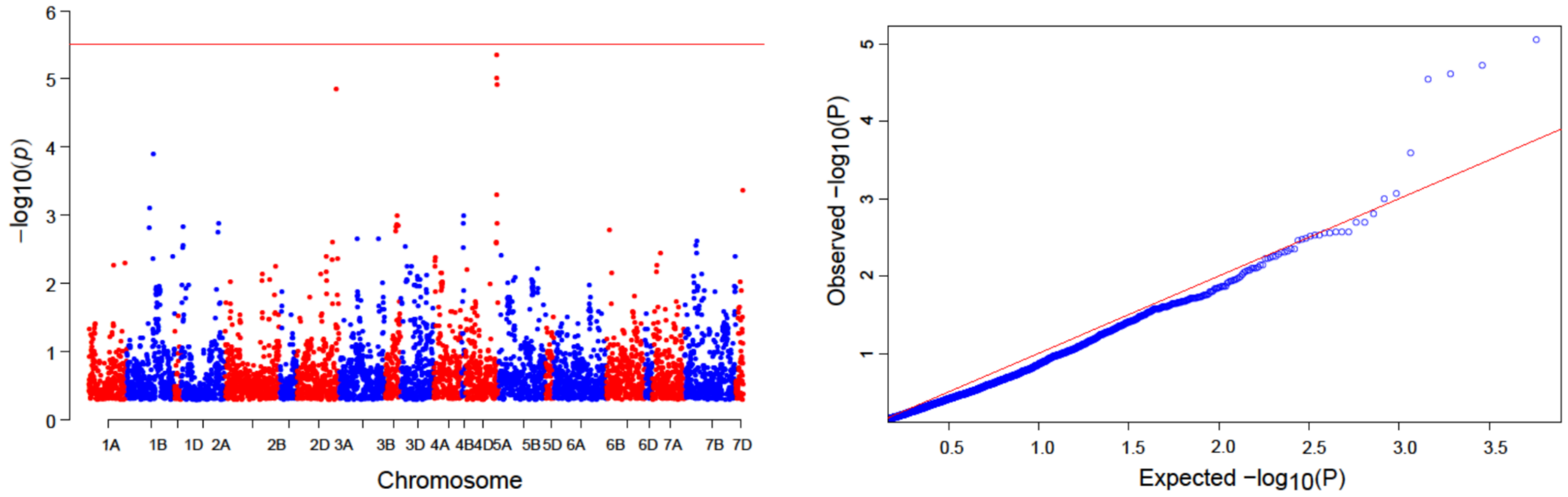

FLW2017

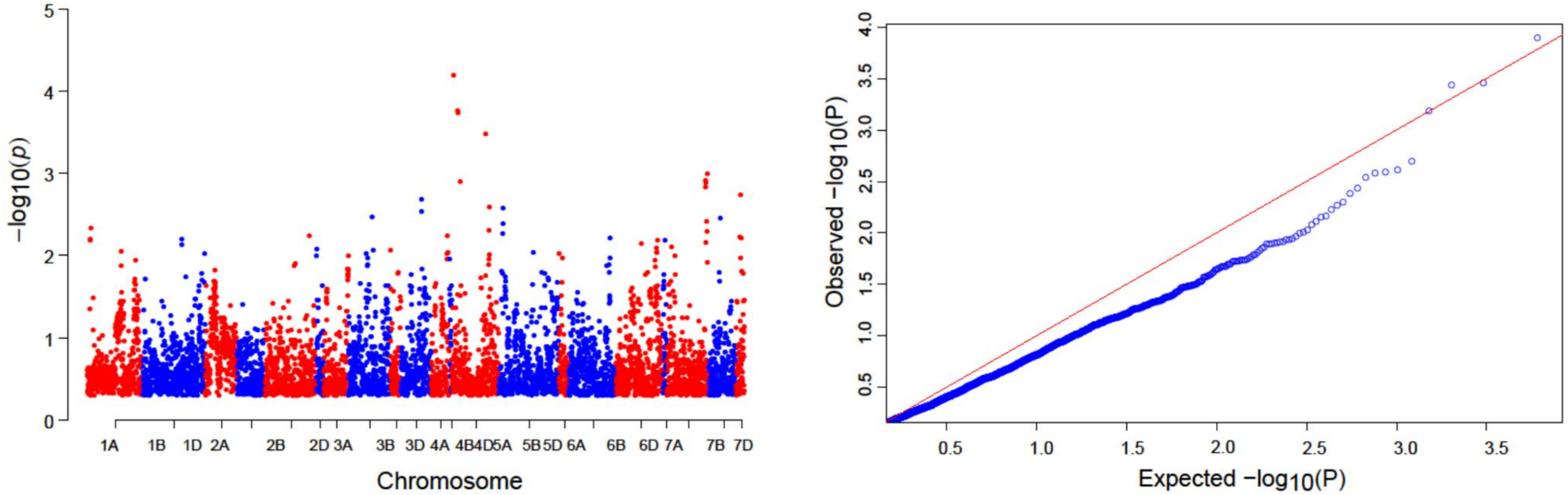

FLW2018

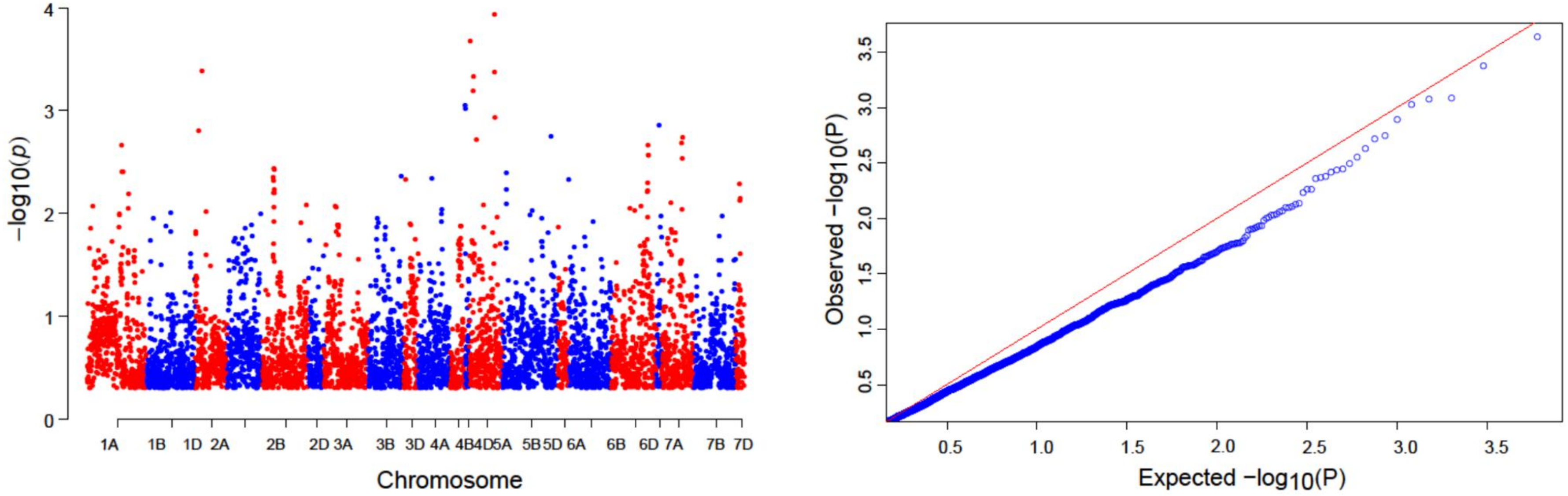

TILL2017

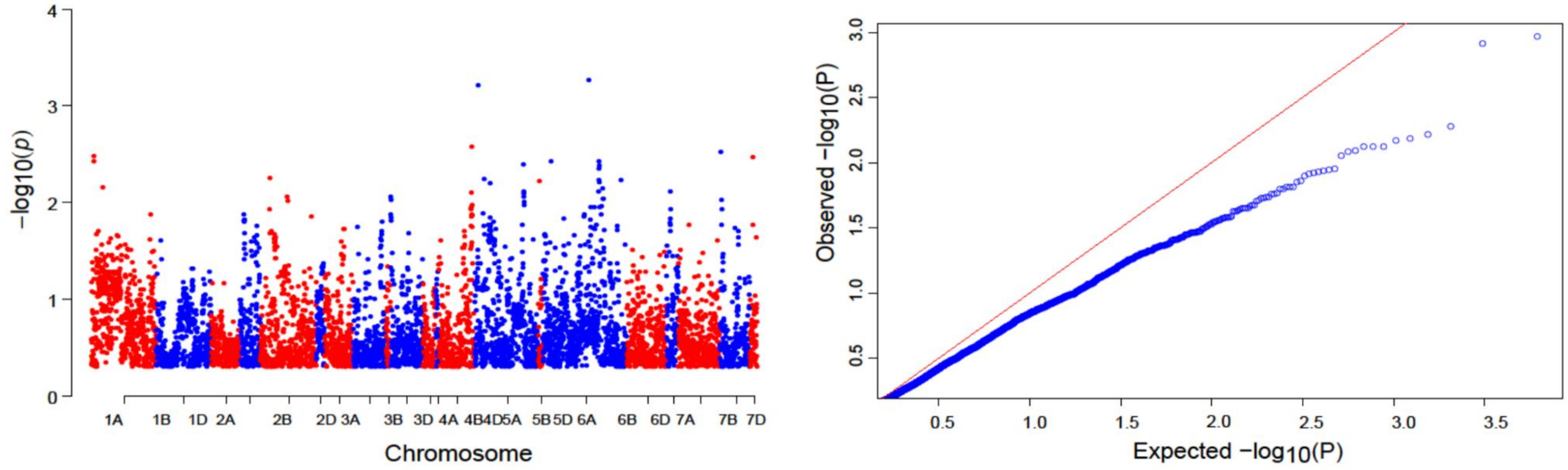

TILL2018

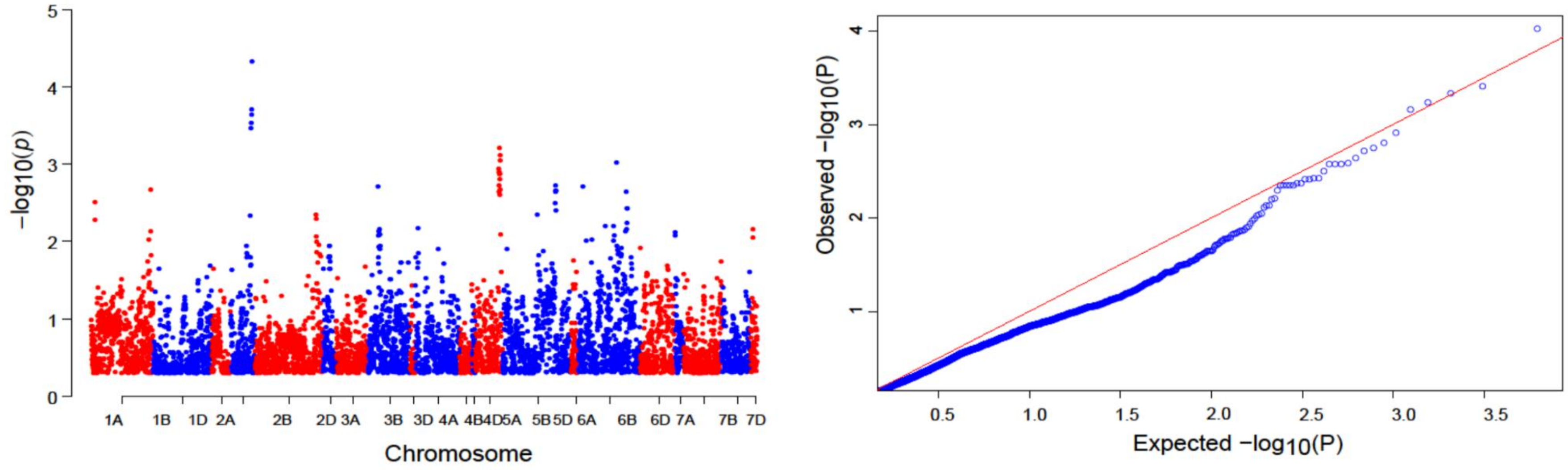

Figure S3b. Manhattan and Q-Q plots of FASTmrEMMA model for plant height, flag leaf length, flag leaf width, and number of tillers across different environments.

PH2015

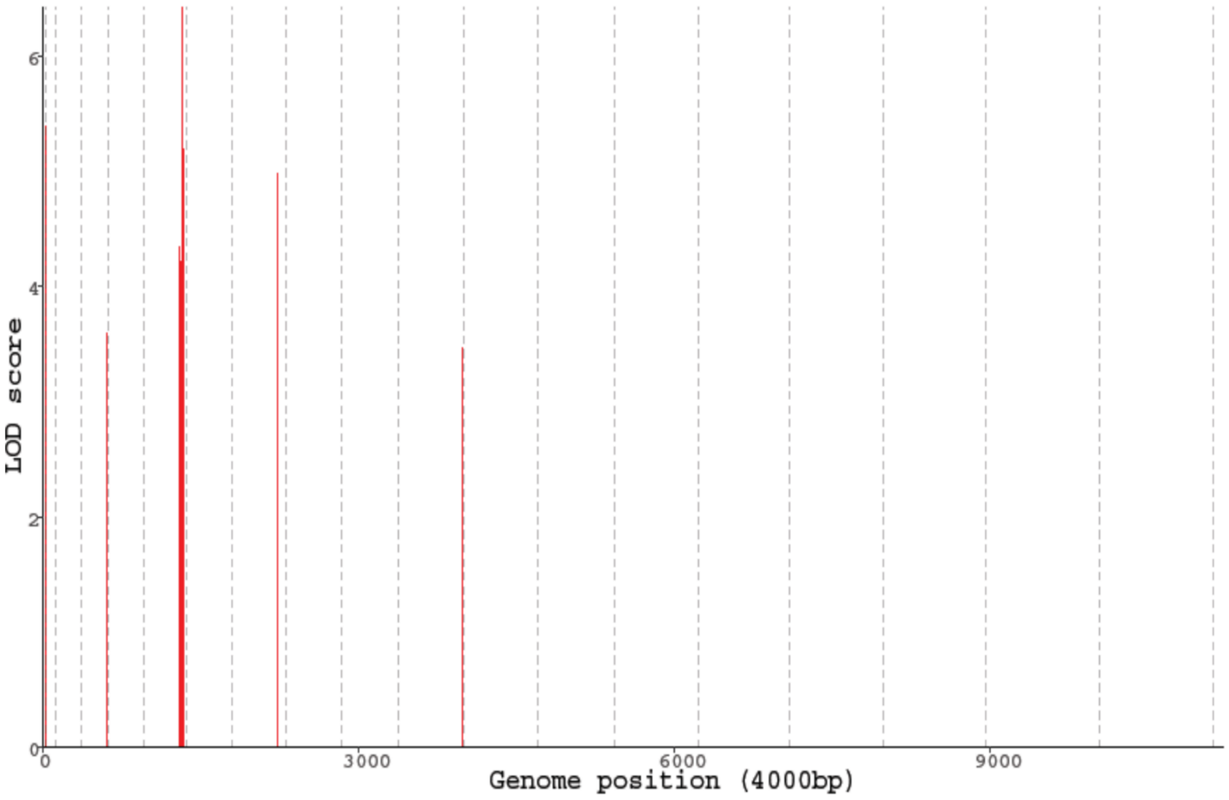

PH2016

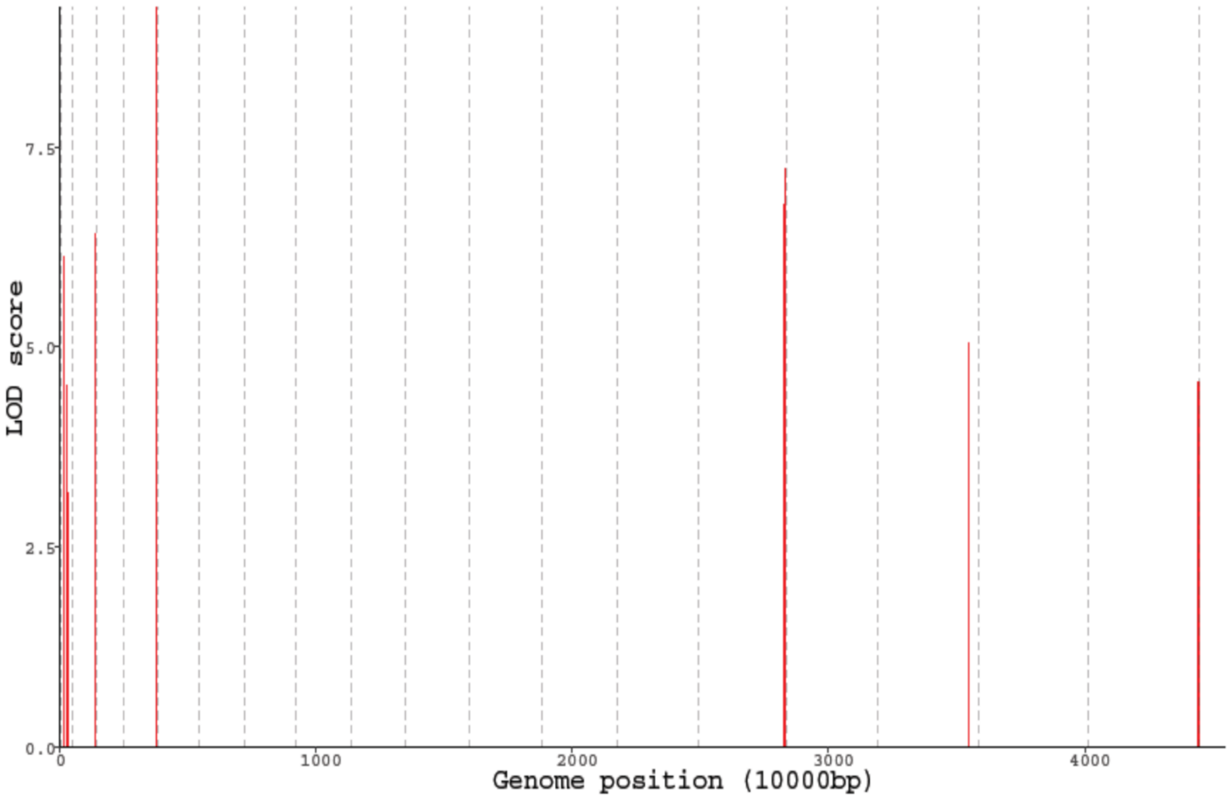

PH2017

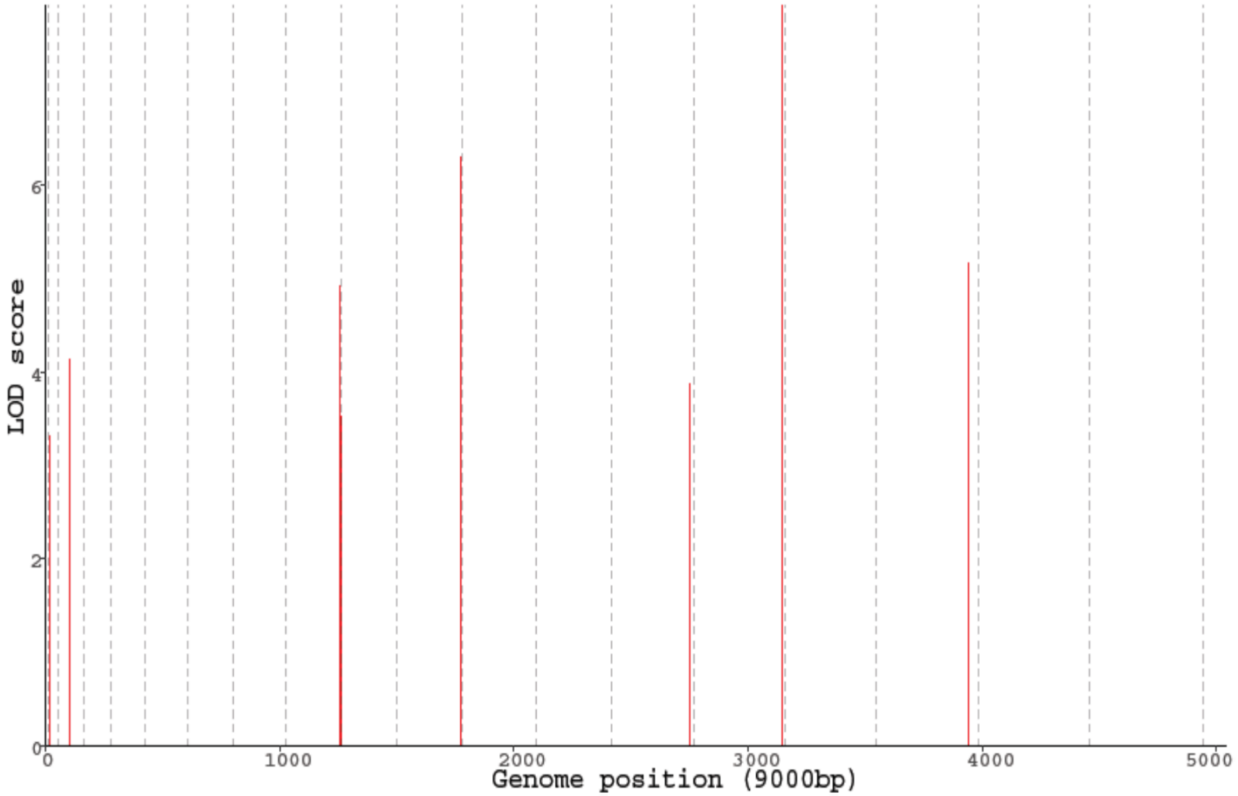

PH2018

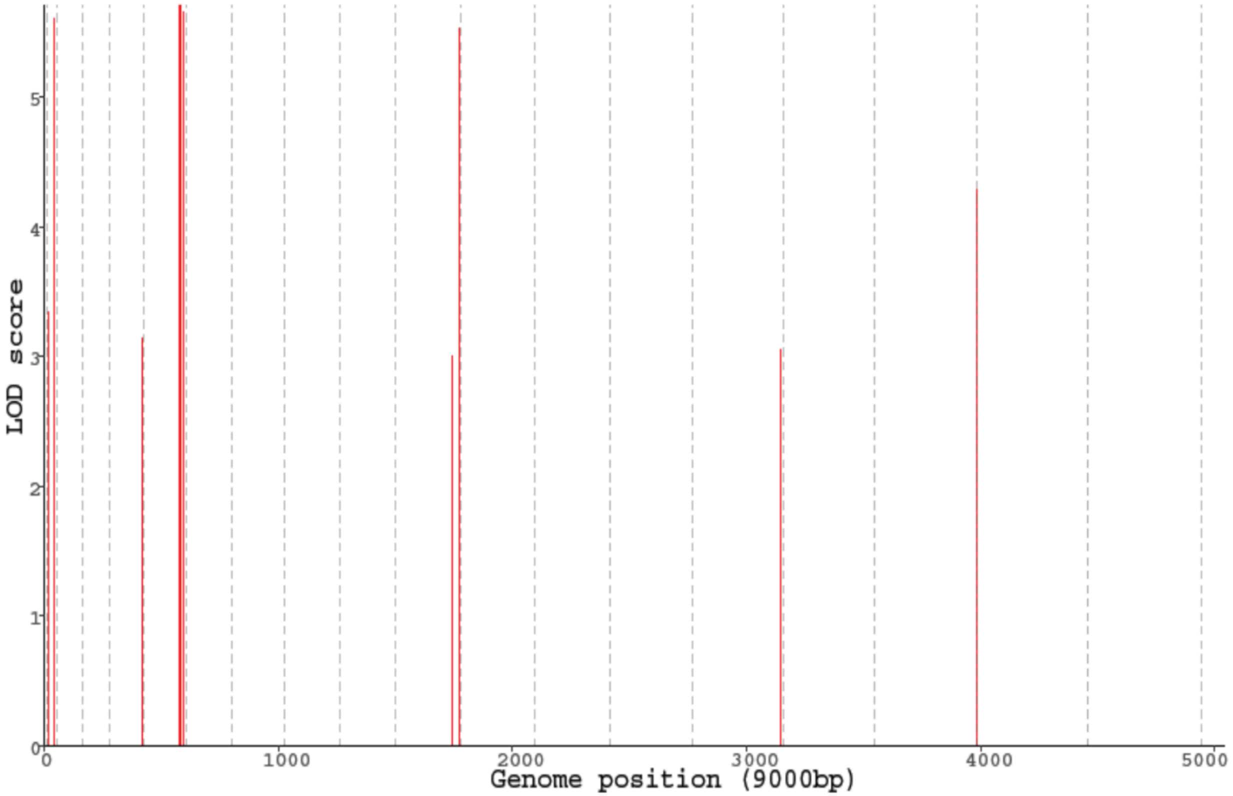

FLL2017

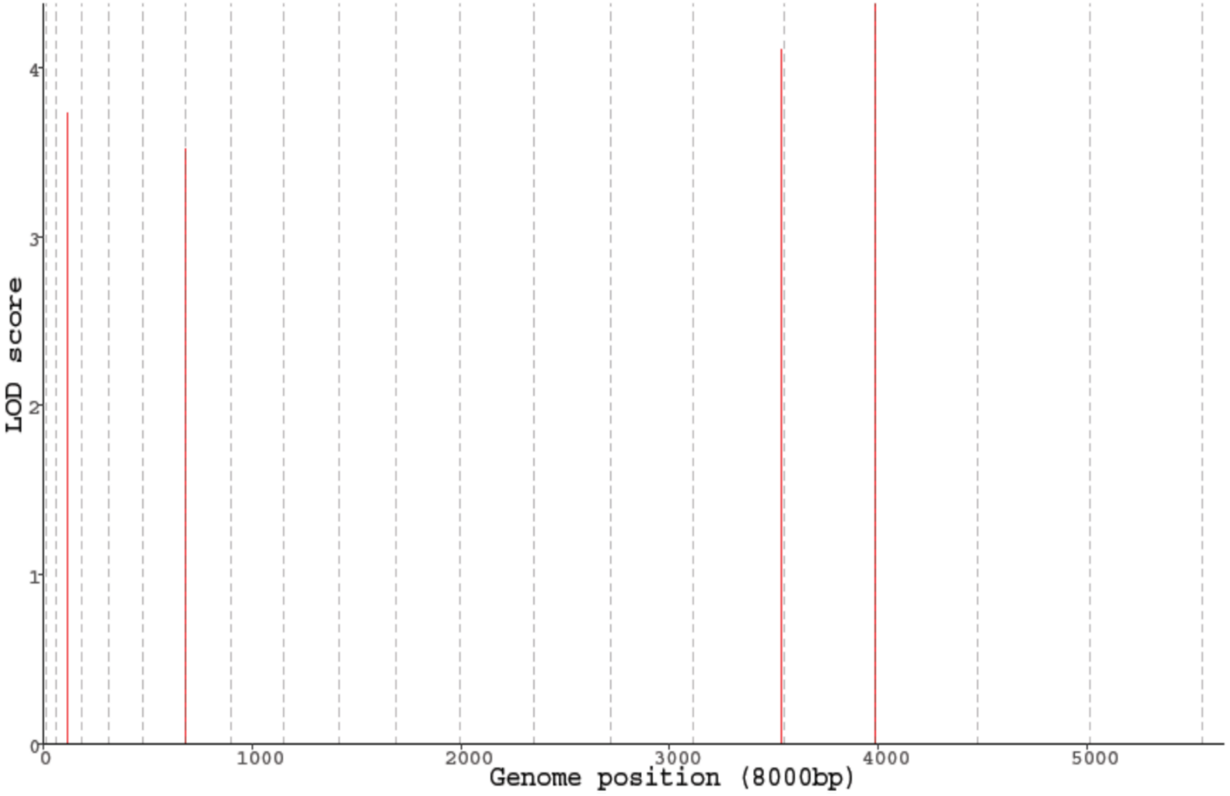

FLL2018

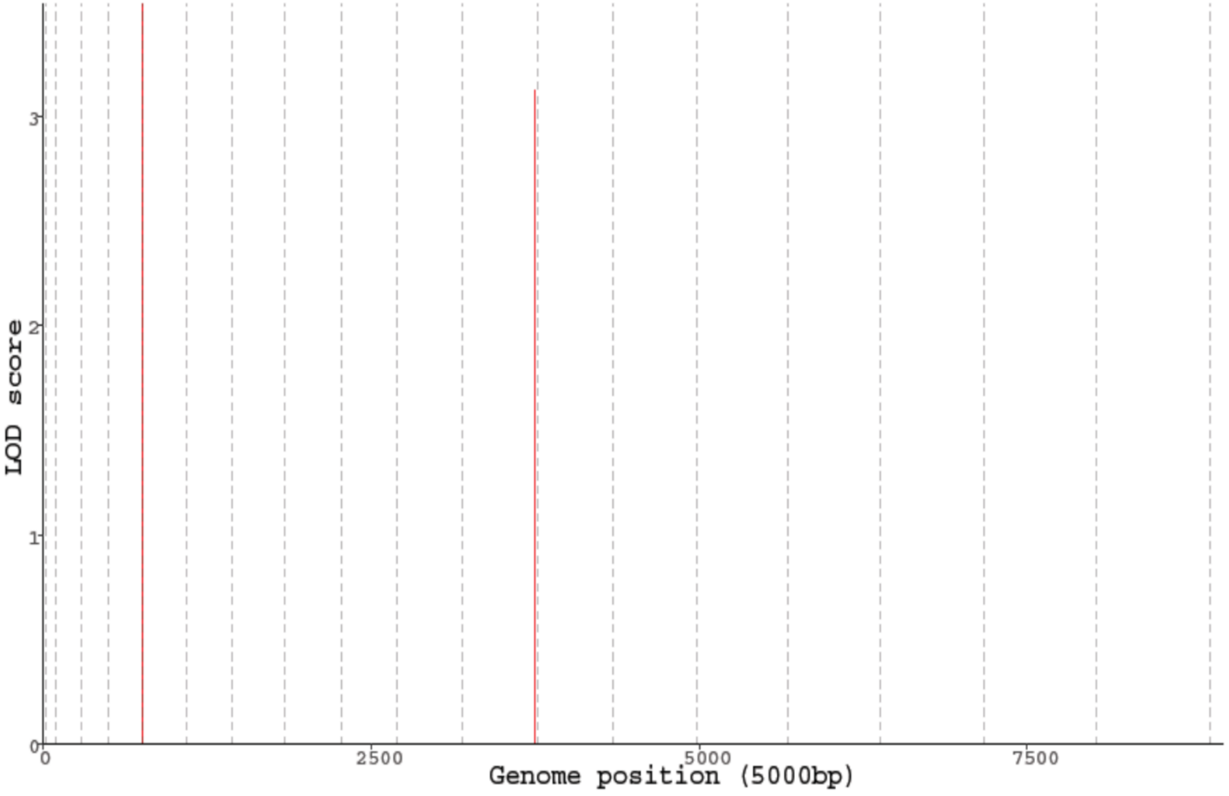

TILLERS 2017

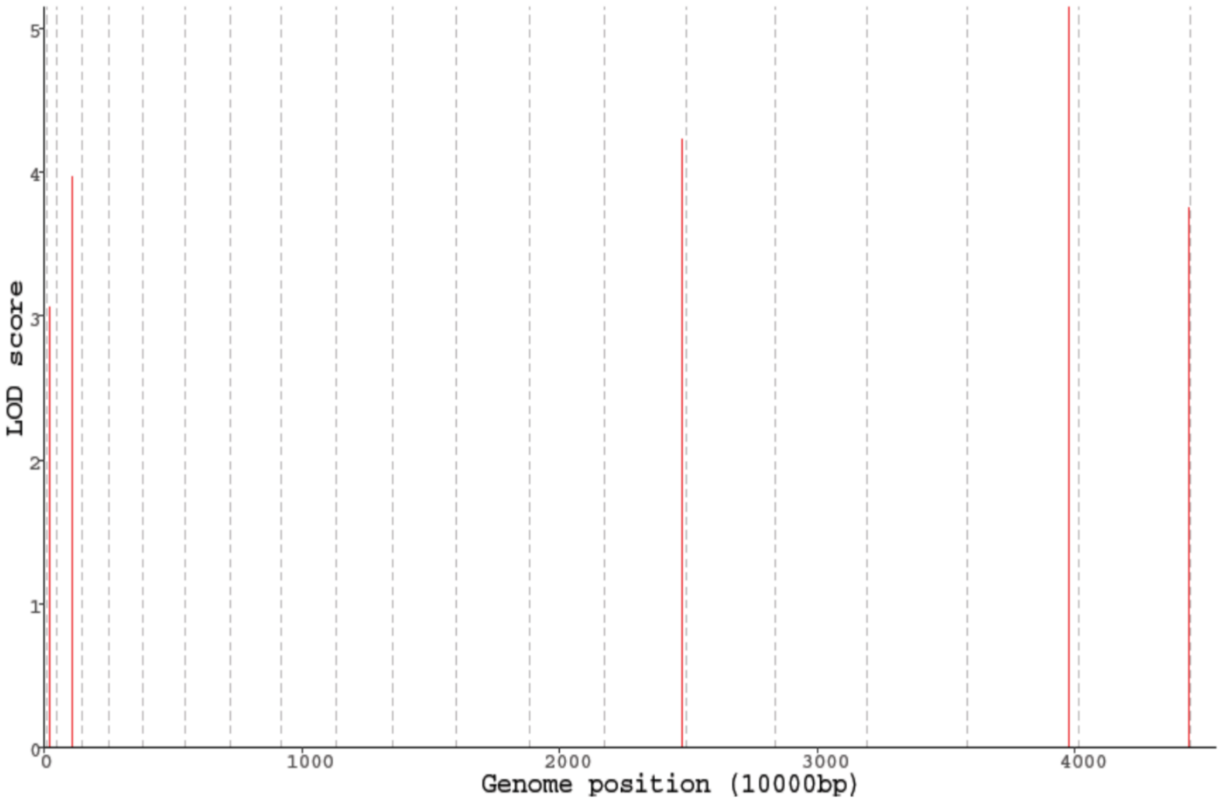

TILLERS 2018

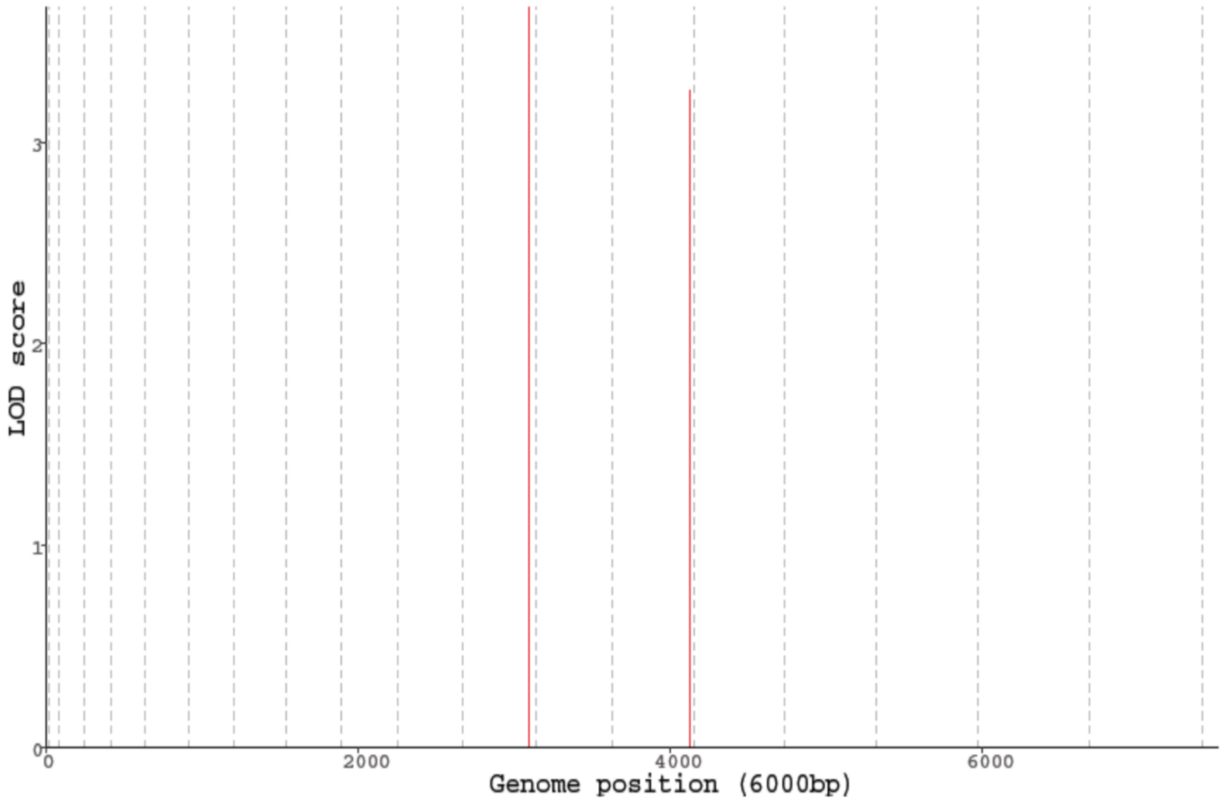

Figure S3c. Manhattan and Q-Q plots of pLARmEB model for plant height, flag leaf length, and number of tillers across different environments.

Figure S4. Manhattan and Q-Q plots of three single locus GWAS models for plant architectural traits (plant height, flag leaf length, flag leaf width, and number of tillers) across different environments.

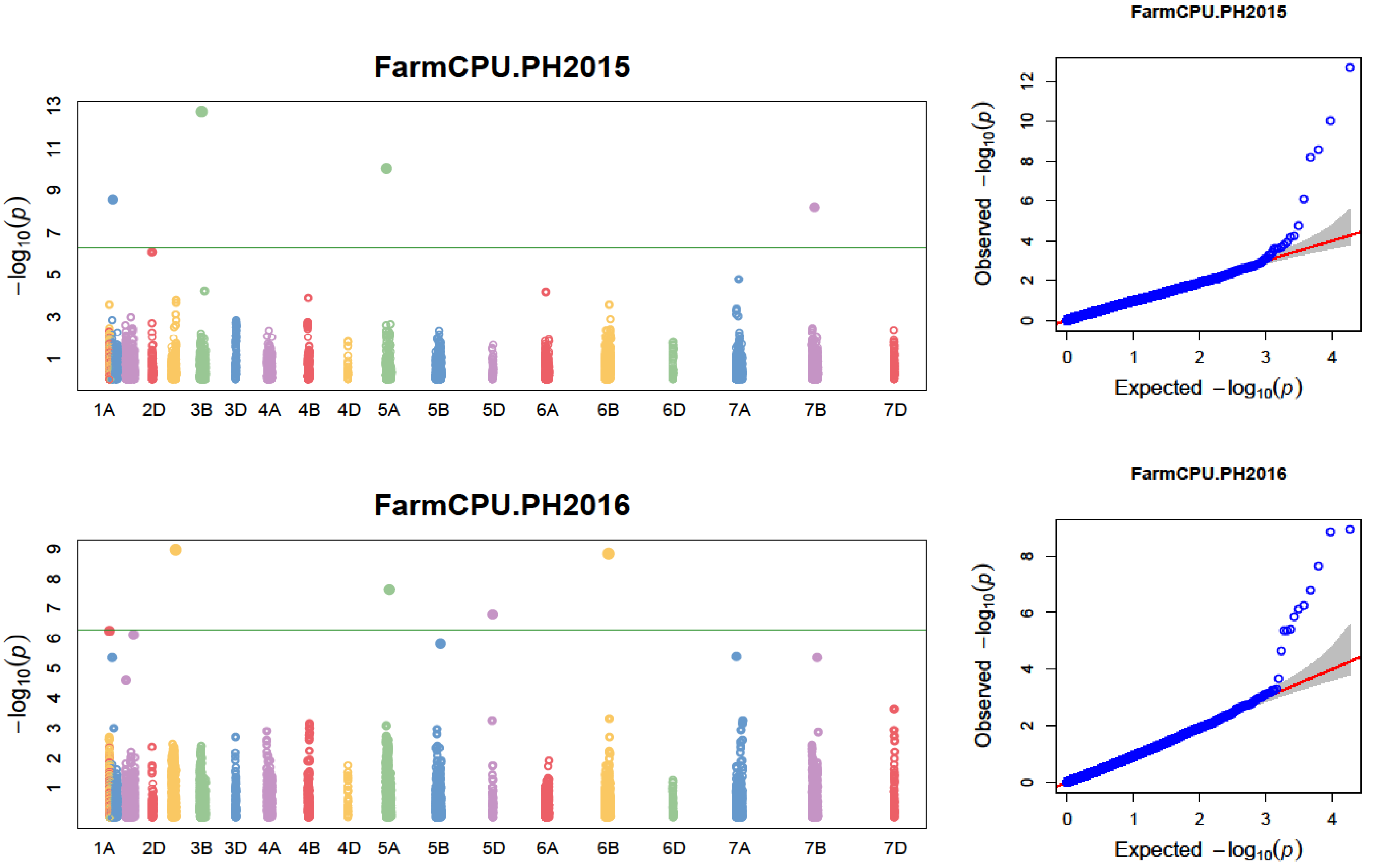
FarmCPU.PH2015
FarmCPU.PH2015
FarmCPU.PH2016
FarmCPU.PH2016
$-\log_{10}(p)$
1A 2D 3B 3D 4A 4B 4D 5A 5B 5D 6A 6B 6D 7A 7B 7D
Observed $-\log_{10}(p)$
Expected $-\log_{10}(p)$

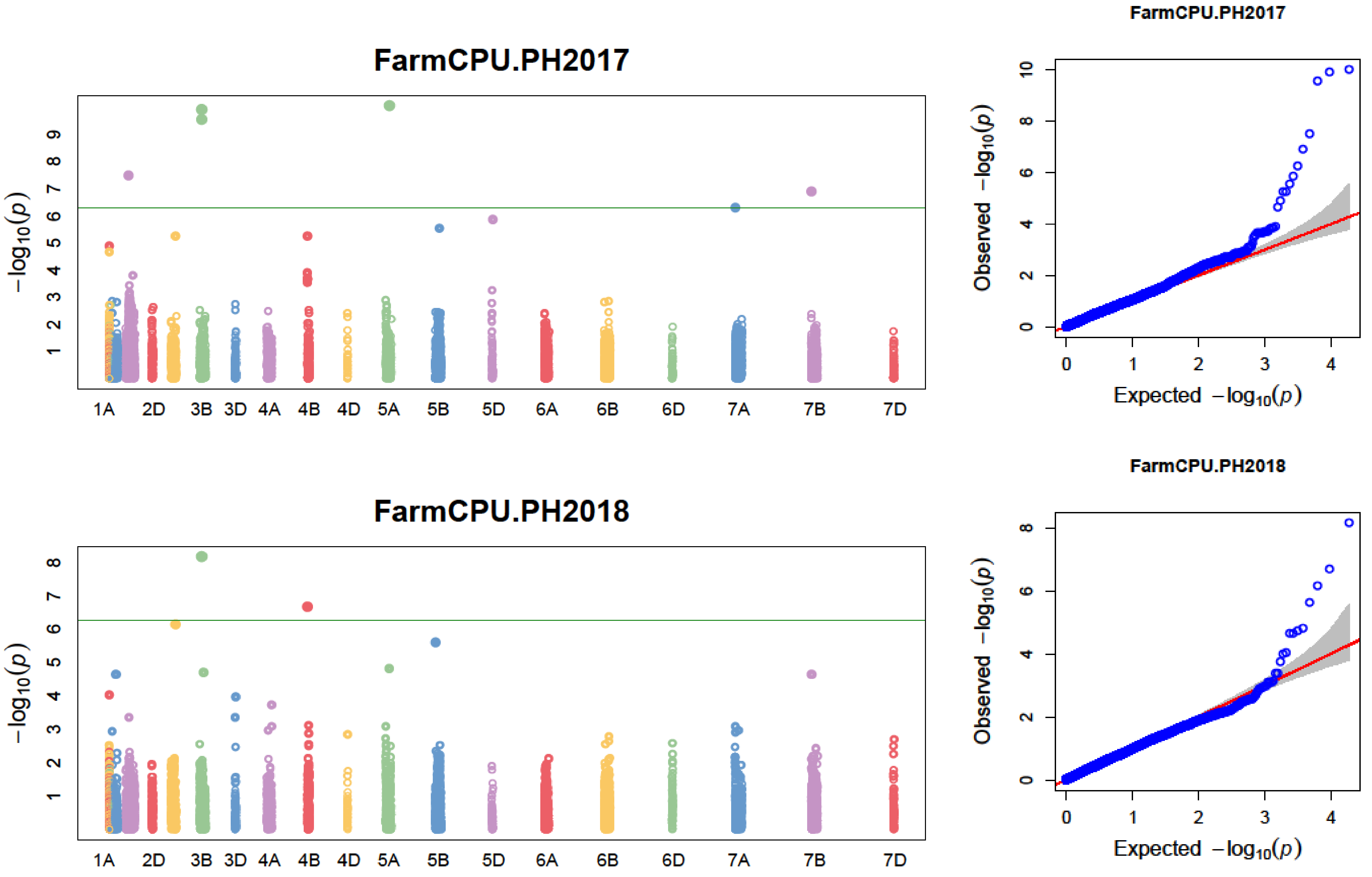
FarmCPU.PH2017
$-\log_{10}(p)$
1A 2D 3B 3D 4A 4B 4D 5A 5B 5D 6A 6B 6D 7A 7B 7D
FarmCPU.PH2017
Observed $-\log_{10}(p)$
Expected $-\log_{10}(p)$
FarmCPU.PH2018
$-\log_{10}(p)$
1A 2D 3B 3D 4A 4B 4D 5A 5B 5D 6A 6B 6D 7A 7B 7D
FarmCPU.PH2018
Observed $-\log_{10}(p)$
Expected $-\log_{10}(p)$

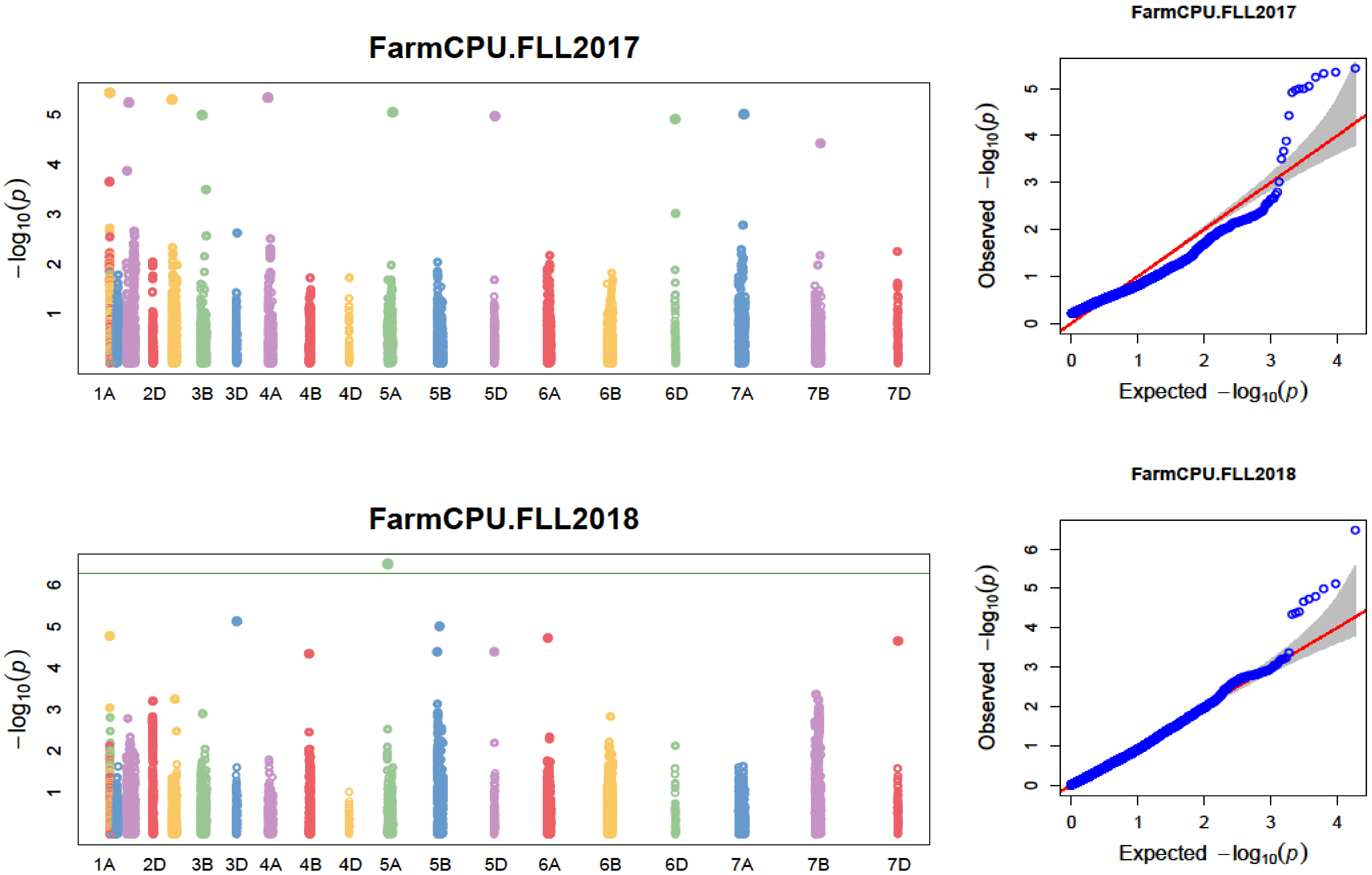
FarmCPU.FLL2017
FarmCPU.FLL2017
−log10(p)
1A 2D 3B 3D 4A 4B 4D 5A 5B 5D 6A 6B 6D 7A 7B 7D
Observed −log10(p)
Expected −log10(p)
FarmCPU.FLL2018
FarmCPU.FLL2018
−log10(p)
1A 2D 3B 3D 4A 4B 4D 5A 5B 5D 6A 6B 6D 7A 7B 7D
Observed −log10(p)
Expected −log10(p)

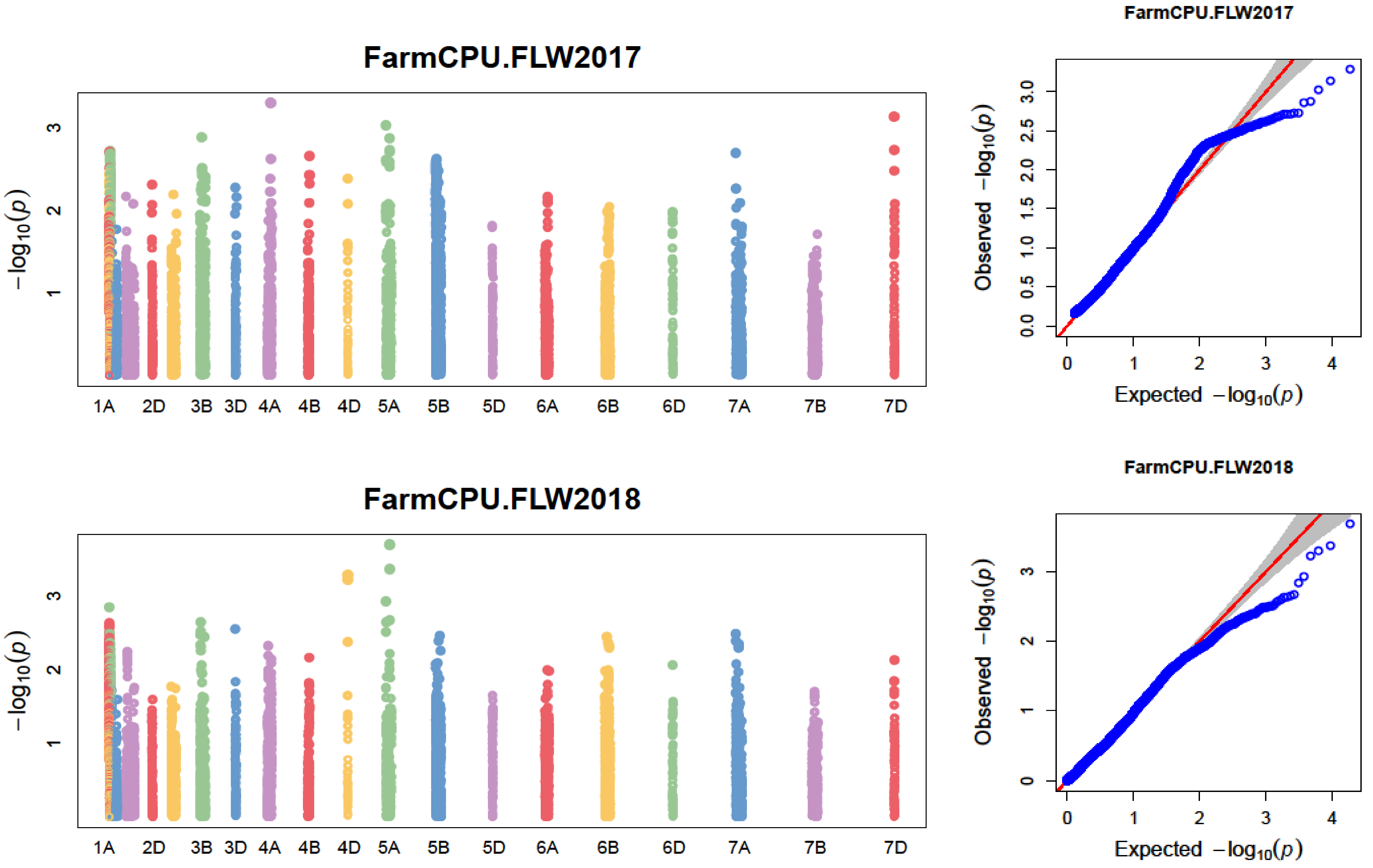
FarmCPU.FLW2017
$-\log_{10}(p)$
1A 2D 3B 3D 4A 4B 4D 5A 5B 5D 6A 6B 6D 7A 7B 7D
FarmCPU.FLW2017
Observed $-\log_{10}(p)$
Expected $-\log_{10}(p)$
FarmCPU.FLW2018
$-\log_{10}(p)$
1A 2D 3B 3D 4A 4B 4D 5A 5B 5D 6A 6B 6D 7A 7B 7D
FarmCPU.FLW2018
Observed $-\log_{10}(p)$
Expected $-\log_{10}(p)$

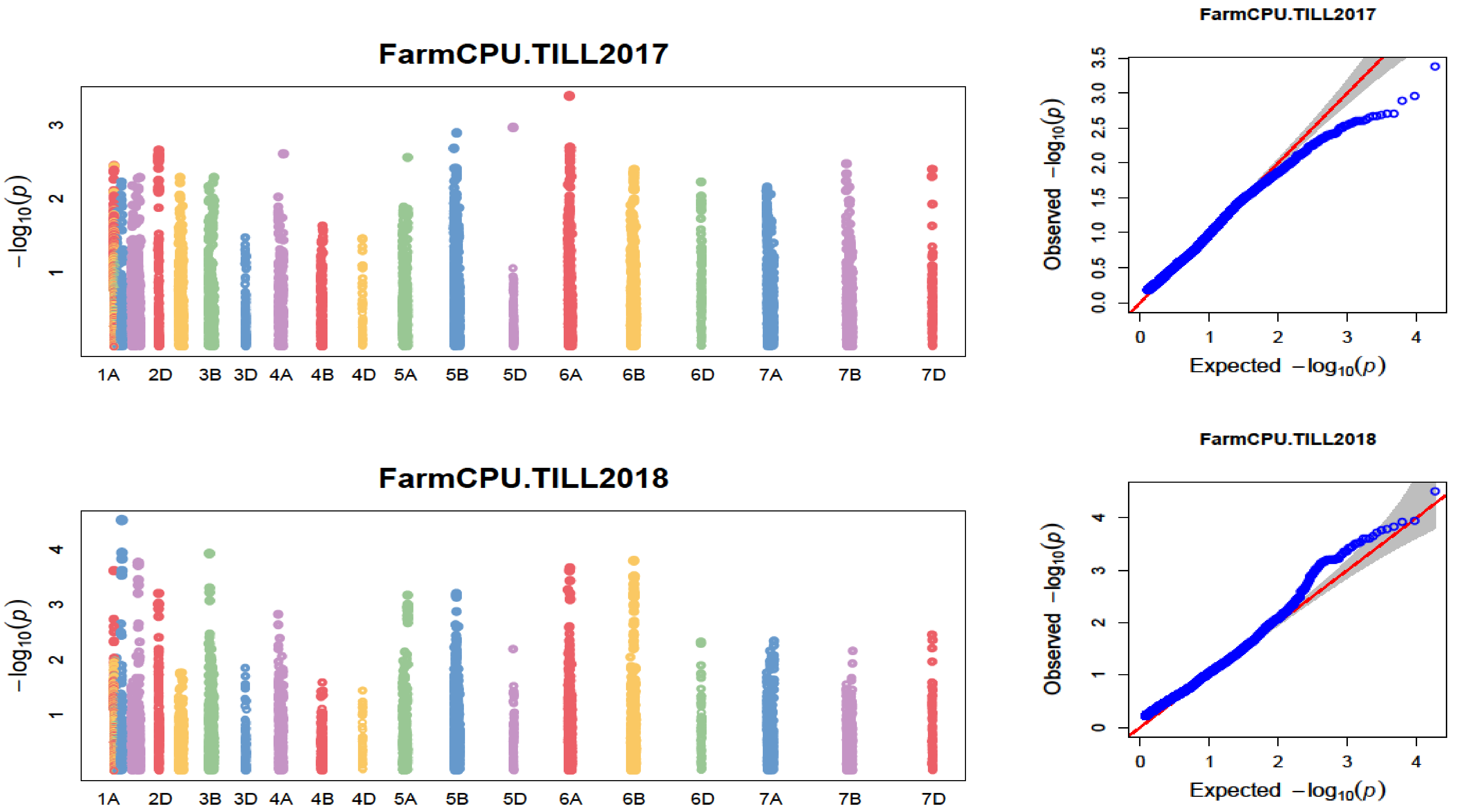

Figure S4a. Manhattan and Q-Q plots of FarmCPU model for plant height, flag leaf length, flag leaf width and number of tillers across different environments.

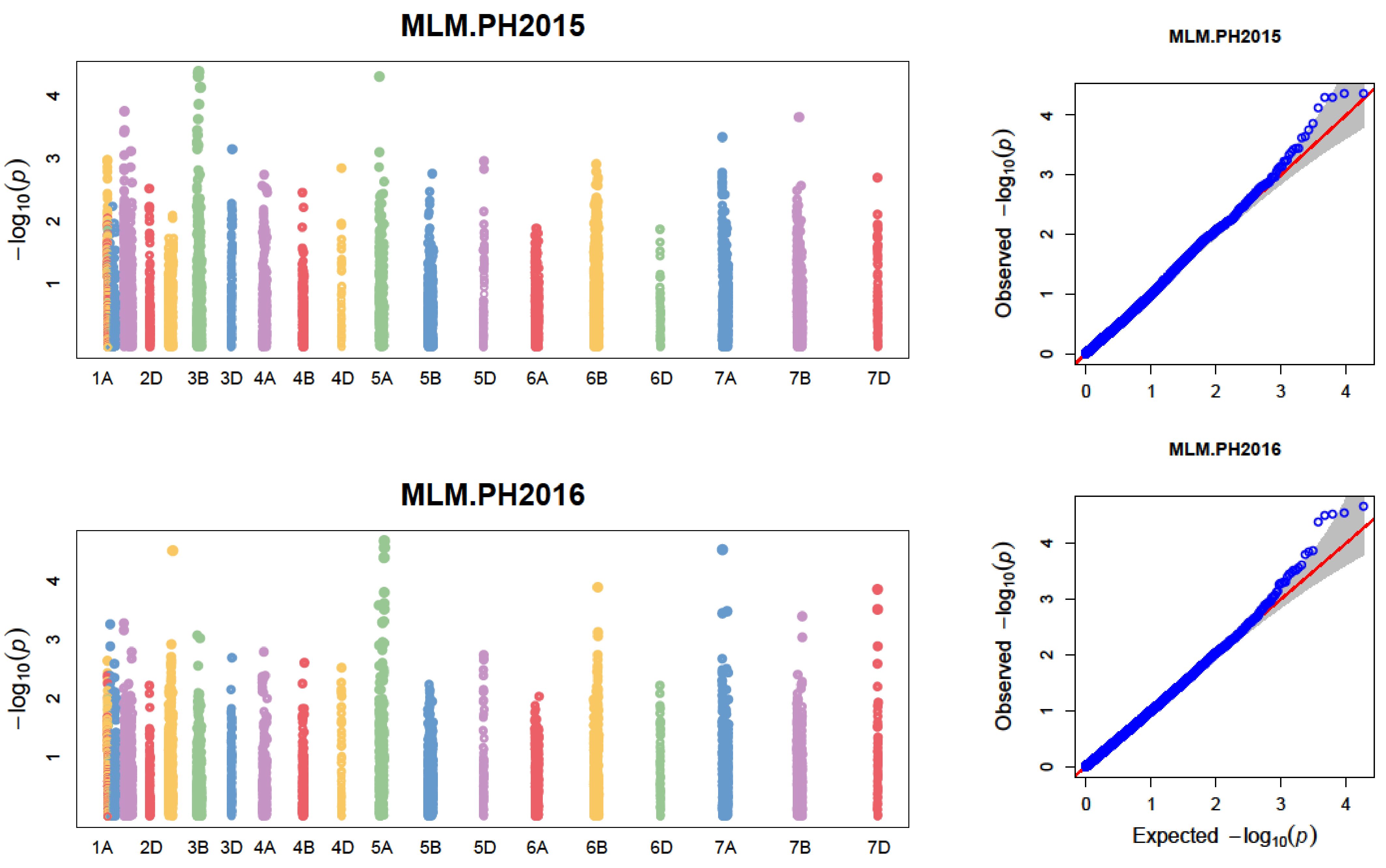
MLM.PH2015
MLM.PH2016
$-\log_{10}(p)$
1A
2D
3B
3D
4A
4B
4D
5A
5B
5D
6A
6B
6D
7A
7B
7D
Observed $-\log_{10}(p)$
Expected $-\log_{10}(p)$

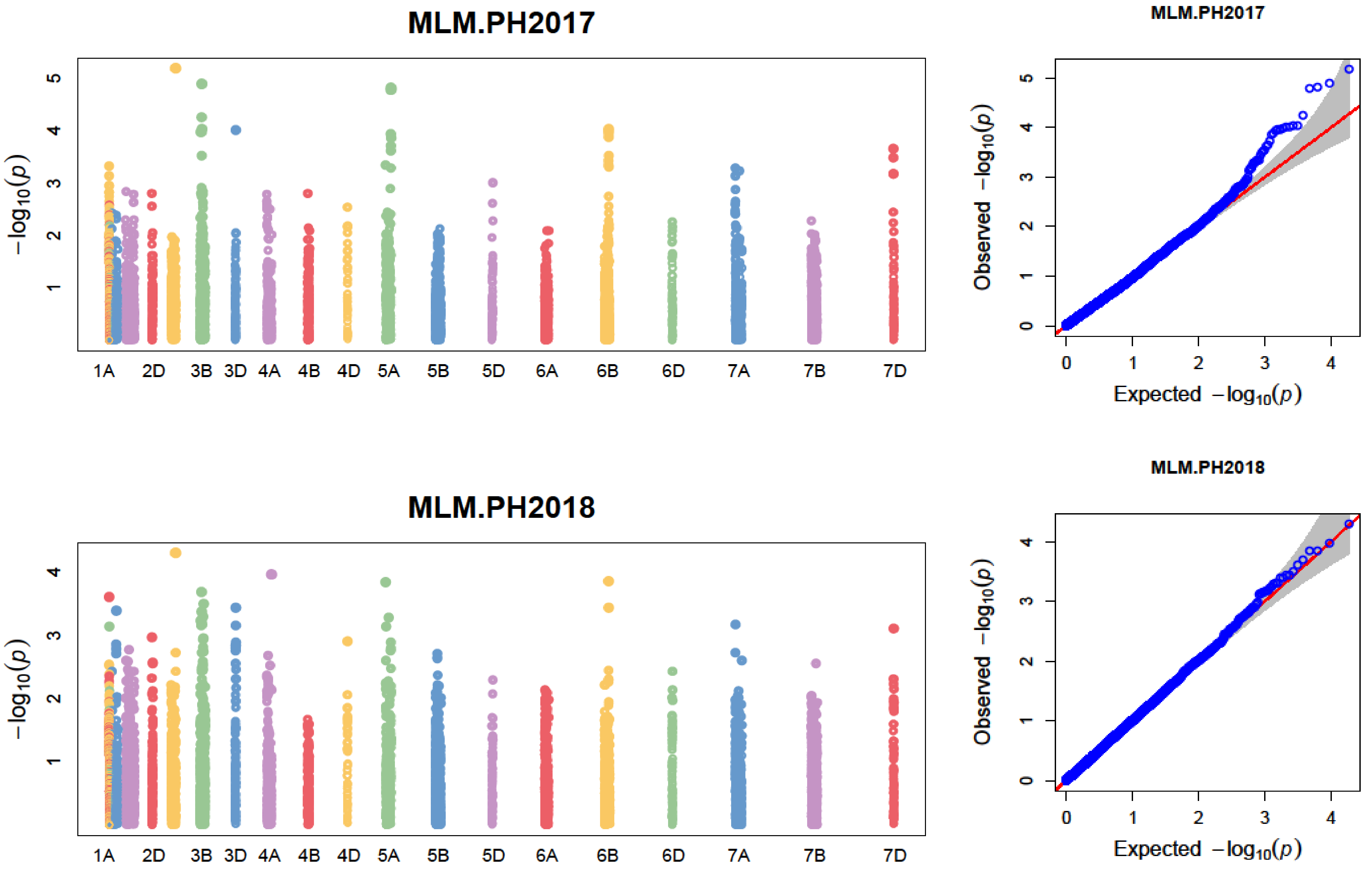
MLM.PH2017
MLM.PH2017
MLM.PH2018
MLM.PH2018
$-\log_{10}(p)$
Observed $-\log_{10}(p)$
Expected $-\log_{10}(p)$
1A
2D
3B
3D
4A
4B
4D
5A
5B
5D
6A
6B
6D
7A
7B
7D

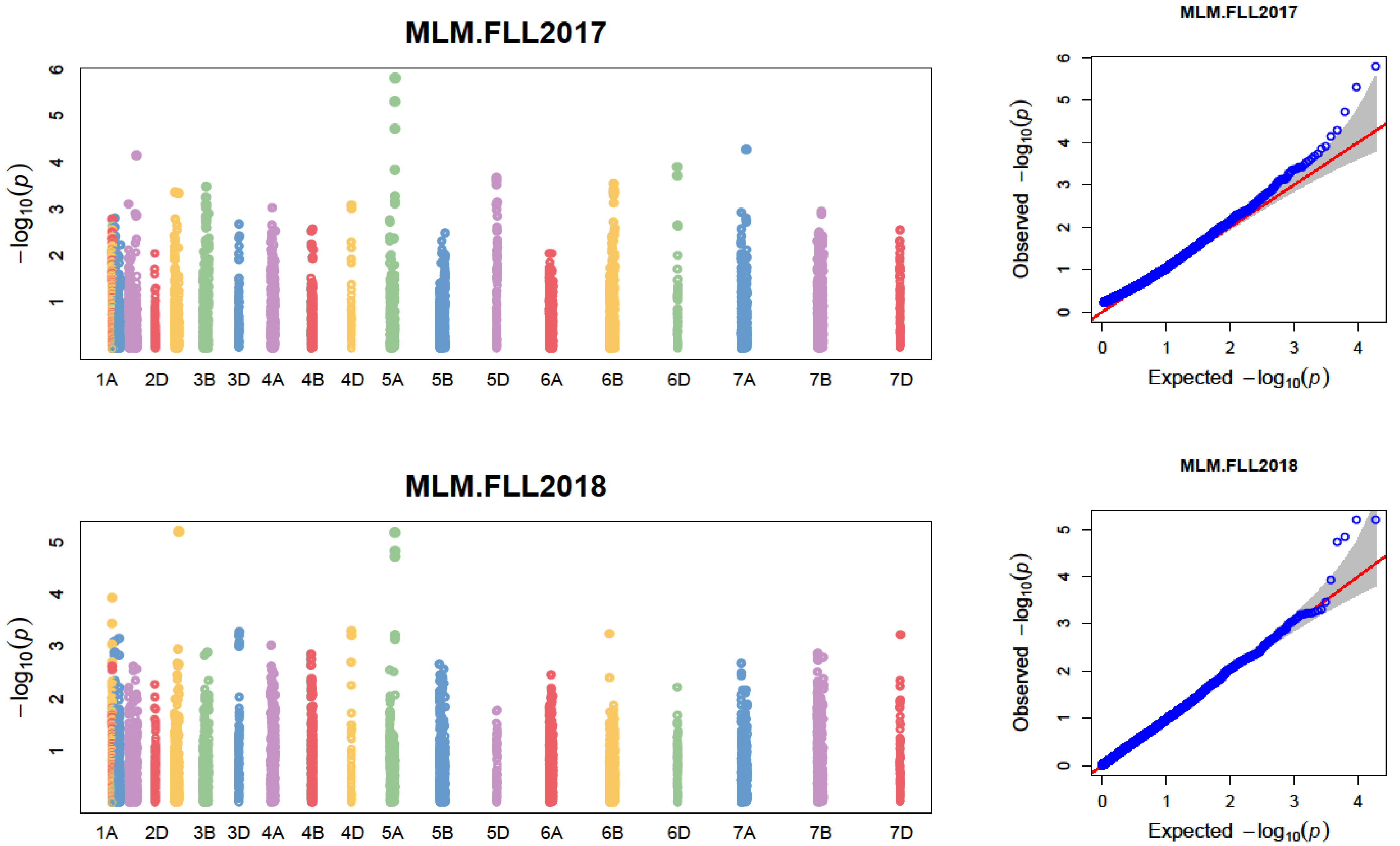
MLM.FLL2017
MLM.FLL2018
$-\log_{10}(p)$
1A 2D 3B 3D 4A 4B 4D 5A 5B 5D 6A 6B 6D 7A 7B 7D
Observed $-\log_{10}(p)$
Expected $-\log_{10}(p)$

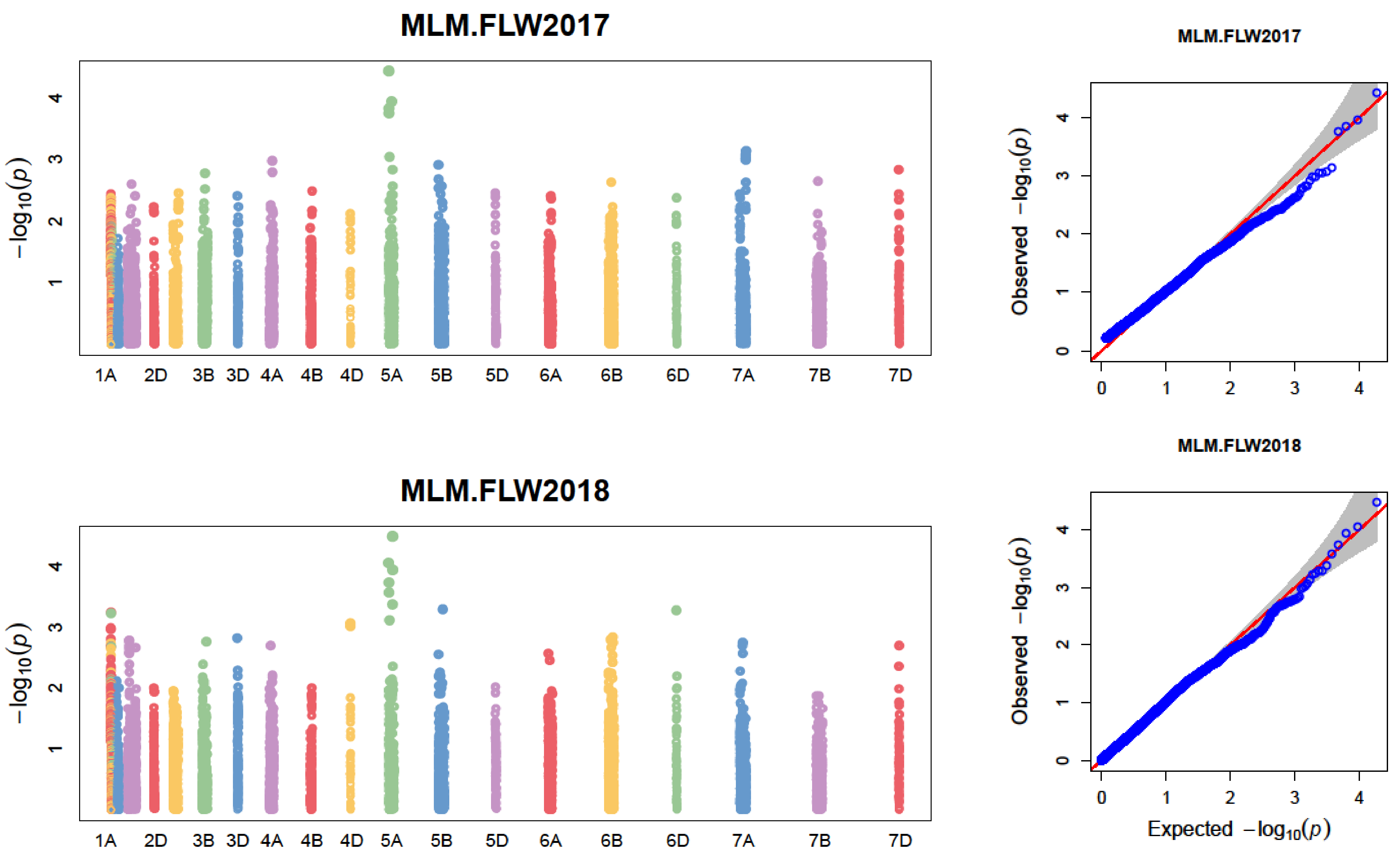

MLM.FLW2017
MLM.FLW2018
$-\log_{10}(p)$
1A
2D
3B
3D
4A
4B
4D
5A
5B
5D
6A
6B
6D
7A
7B
7D
Observed $-\log_{10}(p)$
Expected $-\log_{10}(p)$

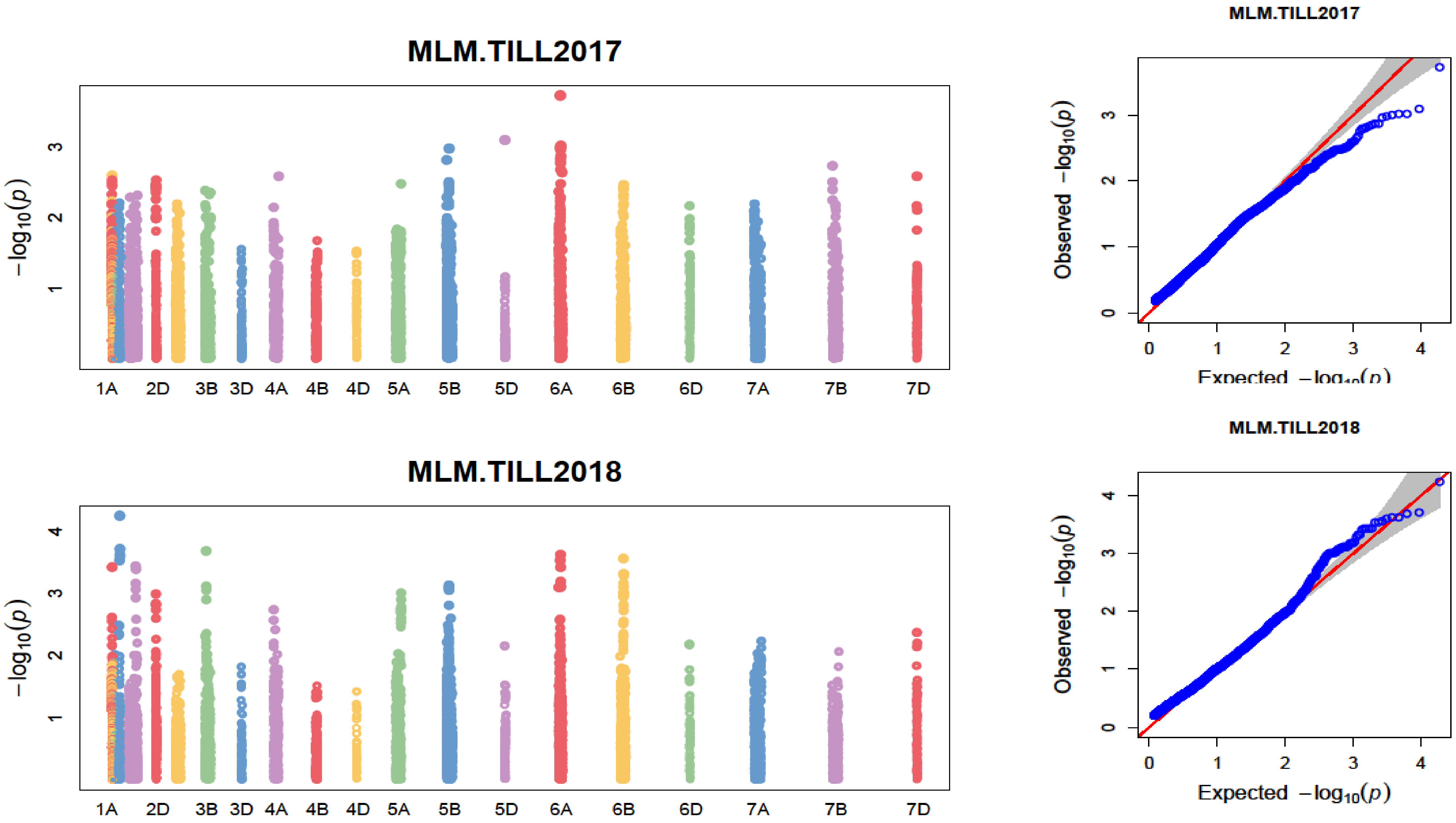

Figure S4b. Manhattan and Q-Q plots of MLM model for plant height, flag leaf length, flag leaf width and number of tillers across different environments.

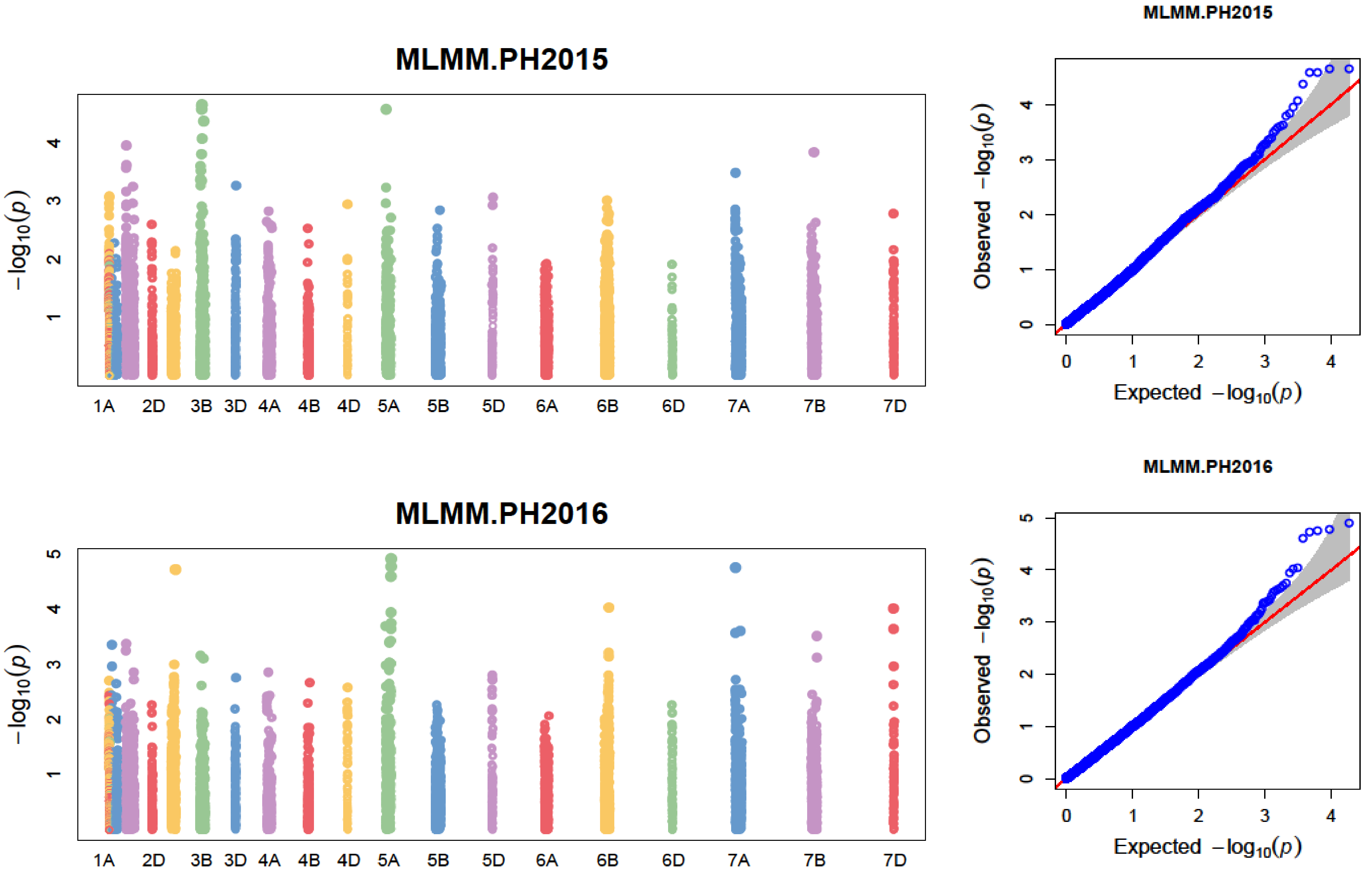
MLMM.PH2015
MLMM.PH2016
$-\log_{10}(p)$
1A
2D
3B
3D
4A
4B
4D
5A
5B
5D
6A
6B
6D
7A
7B
7D
Observed $-\log_{10}(p)$
Expected $-\log_{10}(p)$

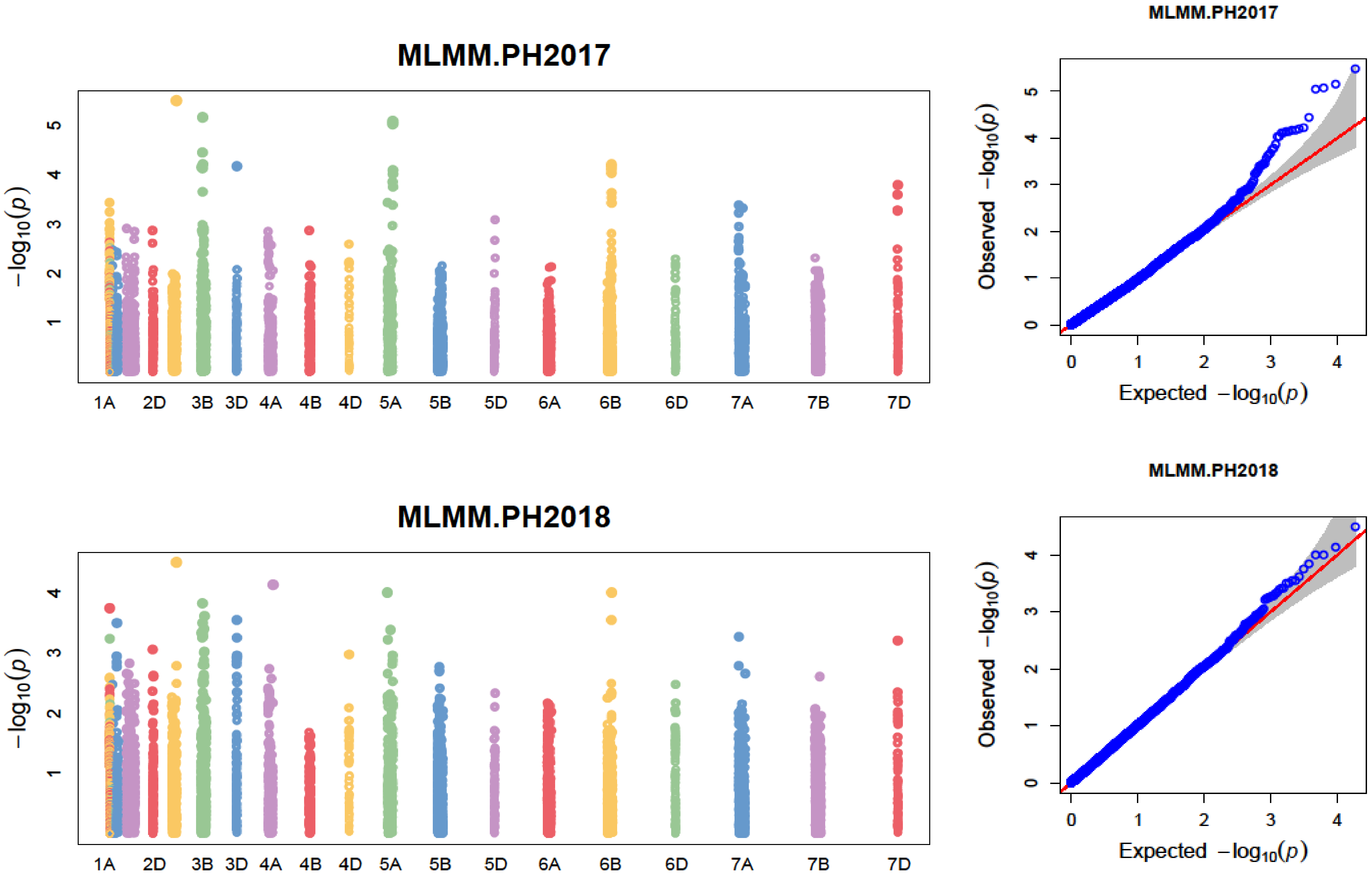
MLMM.PH2017
−log10(p)
1A
2D
3B
3D
4A
4B
4D
5A
5B
5D
6A
6B
6D
7A
7B
7D
MLMM.PH2017
Observed −log10(p)
Expected −log10(p)
MLMM.PH2018
−log10(p)
1A
2D
3B
3D
4A
4B
4D
5A
5B
5D
6A
6B
6D
7A
7B
7D
MLMM.PH2018
Observed −log10(p)
Expected −log10(p)

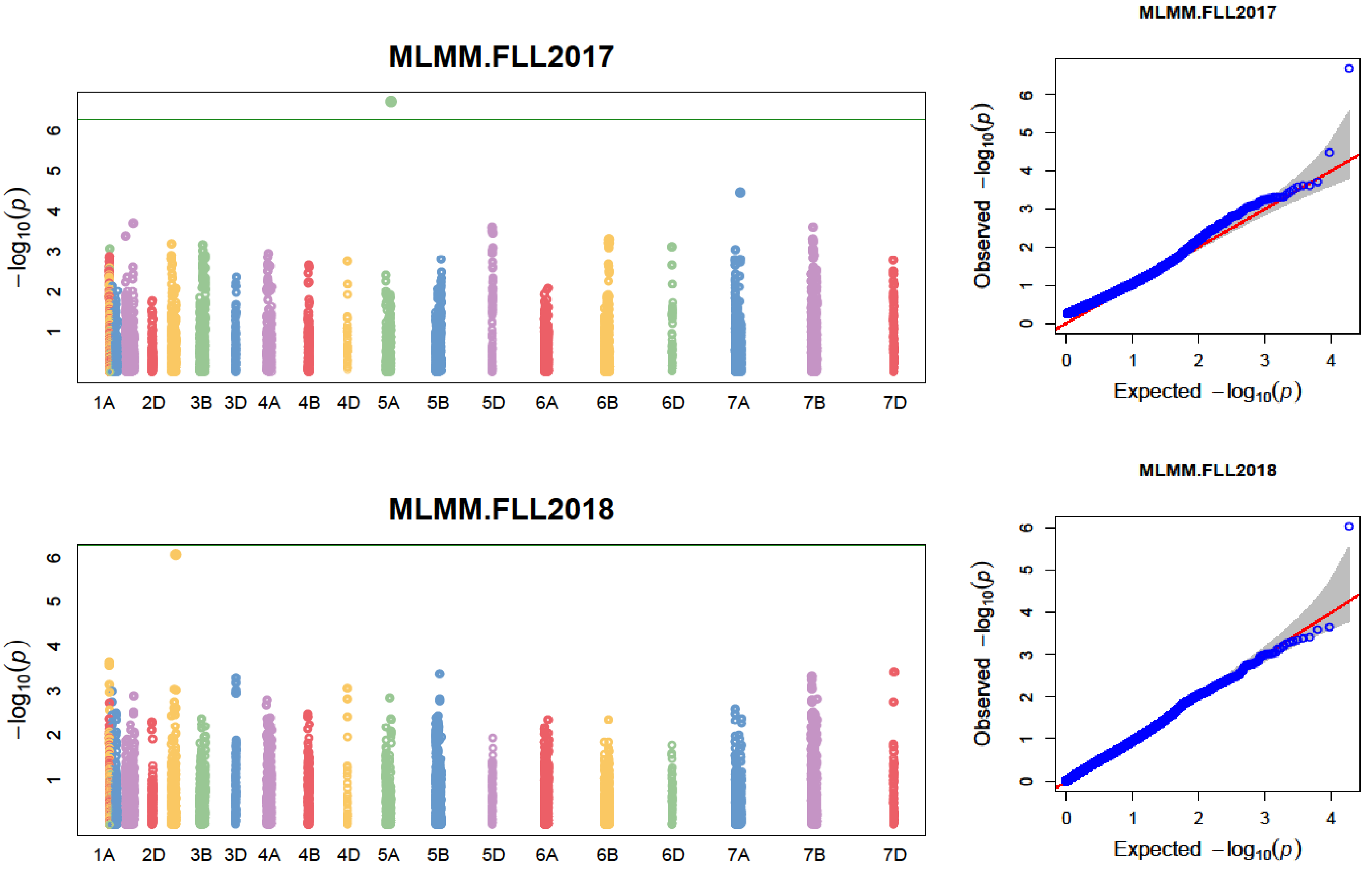
MLMM.FLL2017
MLMM.FLL2018
$-\log_{10}(p)$
1A
2D
3B
3D
4A
4B
4D
5A
5B
5D
6A
6B
6D
7A
7B
7D
Observed $-\log_{10}(p)$
Expected $-\log_{10}(p)$

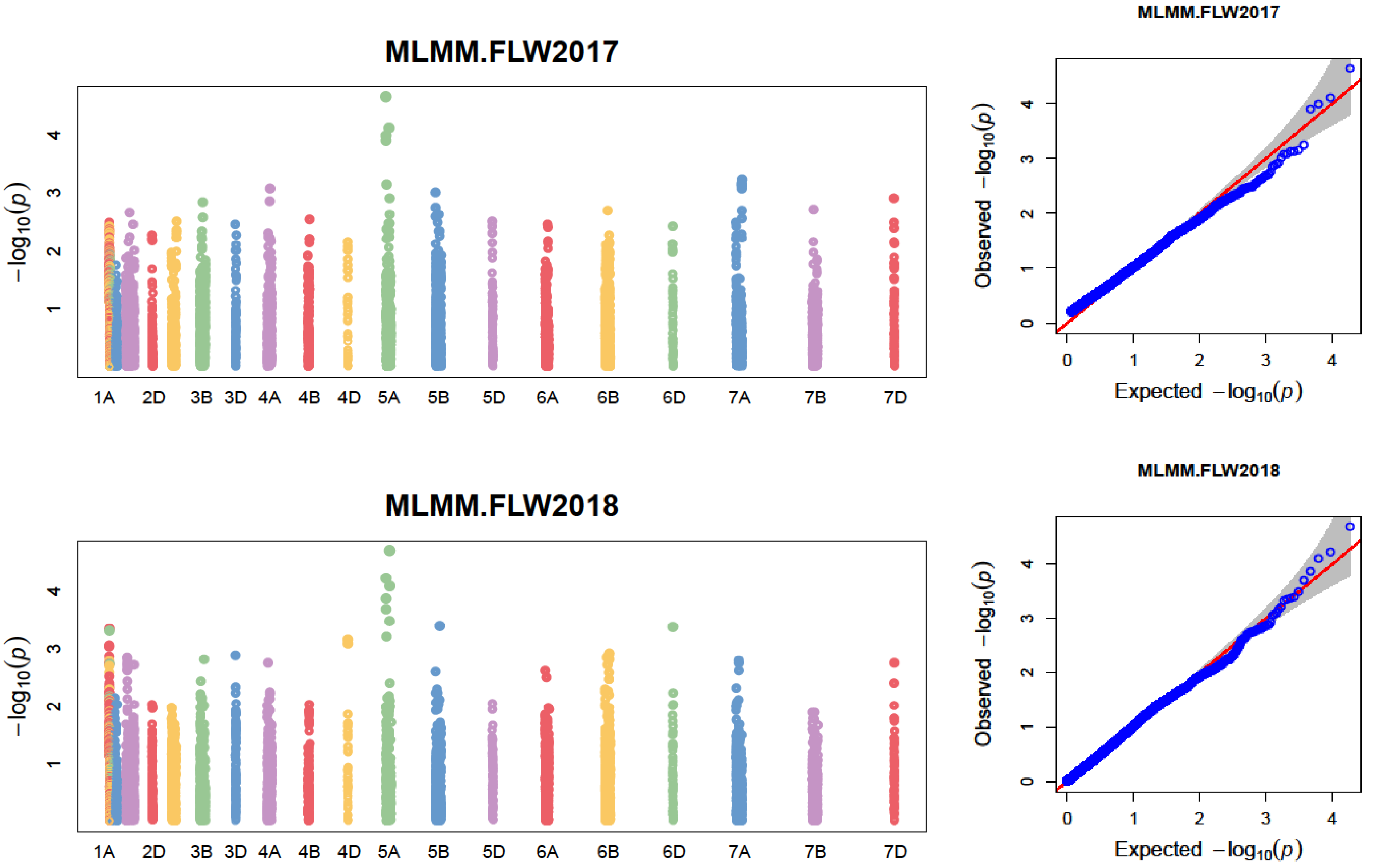

MLMM.FLW2017
−log10(p)
1A
2D
3B
3D
4A
4B
4D
5A
5B
5D
6A
6B
6D
7A
7B
7D
MLMM.FLW2017
Observed −log10(p)
Expected −log10(p)
MLMM.FLW2018
−log10(p)
1A
2D
3B
3D
4A
4B
4D
5A
5B
5D
6A
6B
6D
7A
7B
7D
MLMM.FLW2018
Observed −log10(p)
Expected −log10(p)

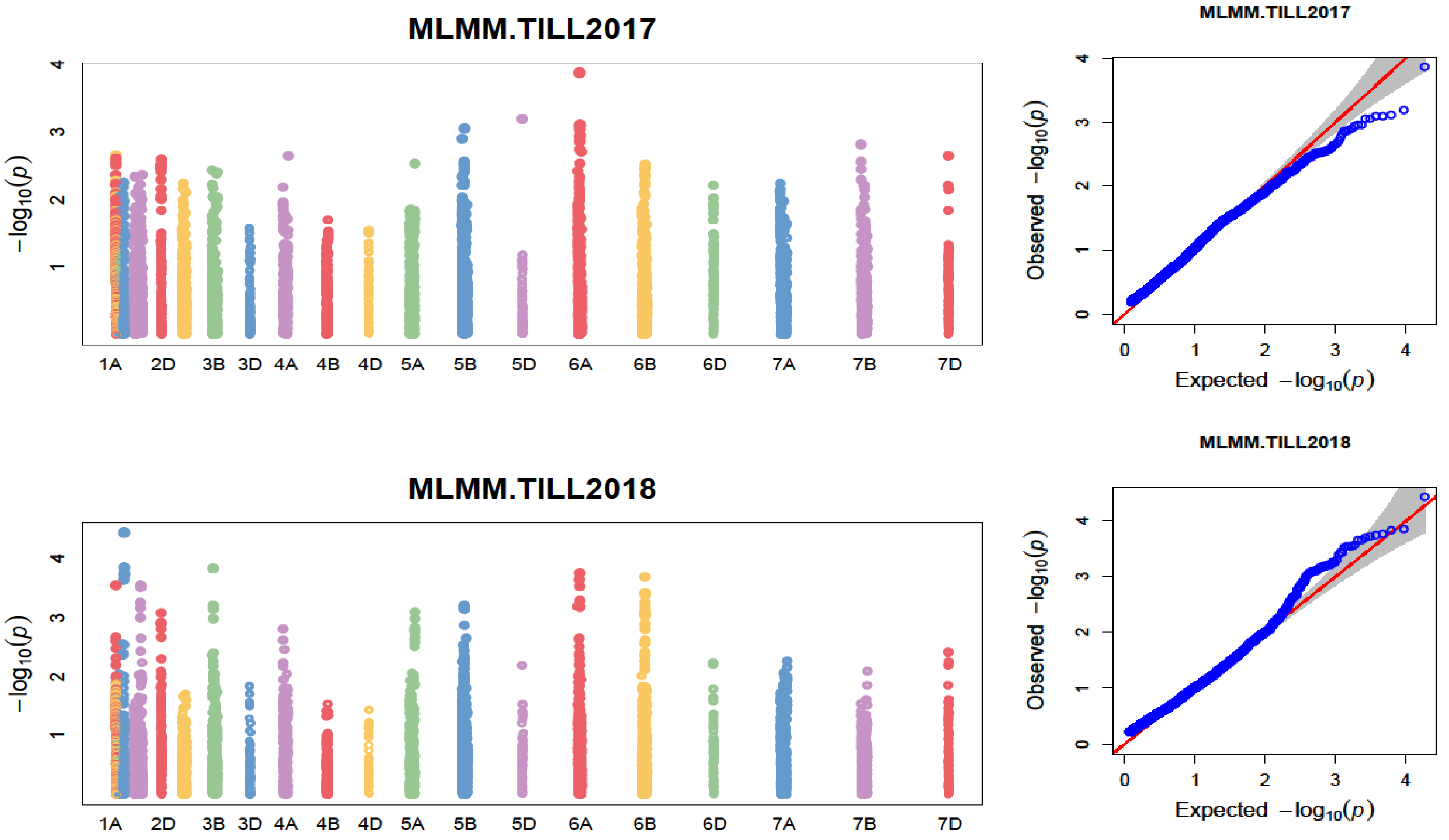

Figure S4c. Manhattan and Q-Q plots of MLMM model for plant height, flag leaf length, flag leaf width and number of tillers across different environments.
