# Supplementary material for: Uncovering genomic regions controlling plant architectural traits in hexaploid wheat using different GWAS models
Source: Sci Rep. 2021 Mar 24;11:6767. doi: 10.1038/s41598-021-86127-z (PMC7990932; doi:10.1038/s41598-021-86127-z)
Supplement: Supplementary file 2 — Supplementary Information 2. [file 41598_2021_86127_MOESM2_ESM.docx]

**Uncovering genomic regions controlling plant architectural traits in hexaploid wheat using different GWAS models**

Ali Muhammad^1,2, 4^, Jianguo Li^2^, Weichen Hu^2^, Jinsheng Yu^3^, Shahid Ullah Khan^5^, Muhammad Hafeez Ullah Khan^5^, Guosheng Xie^2^, Jibin Wang^1^ and Lingqiang Wang^1,2,^*

^1^State Key Laboratory for Conservation and Utilization of Subtropical Agro-Bioresources, College of Agriculture, Guangxi University, 100 Daxue Rd., Nanning, Guangxi, China

^2^College of Plant Science and Technology & Biomass and Bioenergy Research Center, Huazhong Agricultural University, Wuhan 430070, China

^3^College of Agriculture and Food Science, Zhejiang A&F University, Lin'an 311300, China

^4^Department of Agriculture, Abdul Wali Khan University Mardan, Pakistan

^5^National Key Laboratory of Crop Genetic Improvement, Huazhong Agricultural University, Wuhan 430070, China

*****Correspondence: [lqwang@gxu.edu.cn](mailto:lqwang@gxu.edu.cn) or [lqwang@mail.hzau.edu.cn](mailto:lqwang@mail.hzau.edu.cn)

**Table S1.** Descriptive statistics of plant architectural traits across multiple environments.

|  | **PH2015** | **PH2016** | **PH2017** | **PH2018** | **FLL2017** | **FLL2018** | **FLW2017** | **FLW2018** | **TILL2017** | **TILL2018** |
| --- | --- | --- | --- | --- | --- | --- | --- | --- | --- | --- |
| Mean | 74.00 | 91.27 | 88.05 | 70.38 | 21.97 | 21.46 | 2.05 | 1.83 | 11.39 | 11.92 |
| SD | 11.08 | 10.12 | 10.60 | 11.34 | 2.30 | 2.31 | 0.26 | 0.18 | 0.91 | 1.07 |
| C.V. | 14.97 | 11.09 | 12.04 | 16.11 | 10.47 | 10.79 | 12.54 | 10.01 | 8.03 | 8.95 |
| Min | 55.55 | 70.54 | 65.63 | 48.40 | 16.16 | 16.06 | 1.22 | 1.33 | 8.10 | 8.33 |
| Max | 112.77 | 139.45 | 136.67 | 124.82 | 30.94 | 31.57 | 2.75 | 2.38 | 14.40 | 14.67 |
| Skew | 1.13 | 1.08 | 1.19 | 1.19 | 0.43 | 0.38 | -0.10 | 0.20 | -0.10 | -0.27 |
| Kurtosis | 1.34 | 1.80 | 2.26 | 2.27 | 1.41 | 0.67 | -0.32 | -0.14 | 0.85 | 0.45 |

PH-Plant height (cm), FLL-Flag leaf length (cm), FLW-Flag leaf width (cm), TILL-Tillers per plant

**Table S2.** Analysis of Variance (ANOVA) and heritability for plant height, flag leaf length, flag leaf width and number of tillers per plant.

| **Trait** | **Source** | **SS** | **DF** | **MS** | **F-calculated** | **P-value** | **F-critical** | ***H*^2^** |
| --- | --- | --- | --- | --- | --- | --- | --- | --- |
| PH | Genotype | 181382 | 312 | 581.4 | 17.45 | 0.00 | 1.23 | 0.91 |
|  | Year | 4877 | 1 | 4877.2 | 146.35 | 0.00 | 6.66 |  |
|  | Geno × Year | 17362 | 312 | 55.6 | 1.67 | 0.00 | 1.23 |  |
|  | Error | 41589 | 1248 | 33.3 |  |  |  |  |
| FLL | Genotype | 5313.706 | 308 | 17.252 | 11.69 | 0.00 | 1.254 | 0.79 |
|  | Year | 79.618 | 1 | 79.618 | 53.98 | 0.00 | 6.676 |  |
|  | Geno × Year | 1264.55 | 308 | 4.106 | 2.78 | 0.00 | 1.254 |  |
|  | Error | 908.511 | 616 | 1.475 |  |  |  |  |
| FLW | Genotype | 46.869 | 307 | 0.153 | 12.06 | 0.00 | 1.254 | 0.81 |
|  | Year | 12.32 | 1 | 12.32 | 972.94 | 0.00 | 6.676 |  |
|  | Geno × Year | 10.152 | 307 | 0.033 | 2.61 | 0.00 | 1.254 |  |
|  | Error | 7.775 | 614 | 0.013 |  |  |  |  |
| Tillers | Genotype | 1535.5 | 311 | 4.94 | 5.24 | 0.00 | 1.23 | 0.81 |
|  | Year | 112.8 | 1 | 112.83 | 119.70 | 0.00 | 6.66 |  |
|  | Geno × Year | 291.9 | 311 | 0.94 | 0.99 | 0.51 | 1.23 |  |
|  | Error | 1172.6 | 1244 | 0.94 |  |  |  |  |

DF (Degree of freedom); SS (Sum of Square); MS (Mean square); *H^2^* (Heritability); PH (Plant height), FLL (Flag leaf length), FLW (Flag leaf width).

**Table S3.** List of major QTLs associated with plant architectural traits.

| **Trait** | **Method** | **Major QTL** | **Chr** | **Position (bp)** | **LOD > 3** | **r^2^ (%)** |
| --- | --- | --- | --- | --- | --- | --- |
| PH | mrMLM | RAC875_c8121_1490 | 3A | 1272572 | 7.068 | 13.879 |
|  | mrMLM | Ku_27771_508 | 3B | 1402891 | 5.878 | 8.383 |
|  | mrMLM | IACX3190 | 3B | 1429597 | 6.349 | 8.513 |
|  | mrMLM | Tdurum_contig42962_2138 | 5A | 2426777 | 4.963 | 7.822 |
|  | mrMLM | Ku_c5191_340 | 6B | 3397322 | 9.297 | 12.988 |
|  | mrMLM | RAC875_c34971_137 | 7A | 3550660 | 9.593 | 9.987 |
| FLL | mrMLM | wsnp_Ex_28733_37836638 | 1B | 789 | 3.368 | 11.406 |
|  | mrMLM | tplb0049a09_1302 | 5A | 2453837 | 7.169 | 13.143 |
|  | FASTmrEMMA | tplb0049a09_2097 | 5A | 2454537 | 6.546 | 7.394 |
|  | mrMLM | Jagger_0882_85 | 7B | 4035135 | 3.554 | 5.771 |
| FLW | FASTmrMLM | IAAV8465 | 7A | 1660873 | 3.703 | 5.769 |
|  | mrMLM | Excalibur_01167_1207 | 5A | 2153585 | 4.647 | 5.743 |
|  | mrMLM | wsnp_Ku_c6977_12078791 | 5A | 2367991 | 5.324 | 6.667 |
|  | mrMLM | RAC875_01969_384 | 7A | 3547319 | 6.525 | 7.534 |
| TILL | mrMLM | BobWhite_07783_174 | 2A | 359541 | 4.106 | 5.932 |
|  | mrMLM | TA001732_0977 | 2B | 580007 | 3.098 | 5.848 |
|  | mrMLM | Kukri_c4750_452 | 6A | 2998535 | 3.465 | 6.298 |
|  | mrMLM | BS00022127_51 | 7B | 3924027 | 4.966 | 5.558 |

PH (Plant height); FLL (Flag leaf length); FLW (Flag leaf width); TILL (Number of tillers per plant). r^2^ (%) represents proportion of total phenotypic variation explained by each QTL.

**Table S4.** Significant SNPs detected through SL-GWAS methods across different environments.

| **Trait** | **Method** | **Year** | **SNP** | **Chr** | **Position (bp)** | **P-Value** |
| --- | --- | --- | --- | --- | --- | --- |
| PH | FarmCPU | 2015 | IACX3190 | 3B | 1429597 | 1.89E-13 |
| PH | FarmCPU | 2015 | Excalibur_rep_008066_112 | 5A | 2179813 | 8.96E-11 |
| PH | FarmCPU | 2015 | JG_c883_445 | 2A | 113990 | 2.74E-09 |
| PH | FarmCPU | 2015 | Ku_c63262_289 | 7B | 4065269 | 6.49E-09 |
| PH | FarmCPU | 2015 | BS00093990_51 | 2D | 957983 | 8.76E-07 |
| PH | FarmCPU | 2015 | wsnp_Ku_c7593_13054436 | 7A | 3691264 | 1.70E-05 |
| PH | FarmCPU | 2016 | RAC875_c8121_1490 | 3A | 1272572 | 1.11E-09 |
| PH | FarmCPU | 2016 | wsnp_Ex_rep_c66315_64480362 | 6B | 3350804 | 1.43E-09 |
| PH | FarmCPU | 2016 | RAC875_03931_205 | 5A | 2358890 | 2.32E-08 |
| PH | FarmCPU | 2016 | Kukri_c7786_81 | 5D | 2874522 | 1.55E-07 |
| PH | FarmCPU | 2016 | Jagger_c6772_80 | 1A | 408 | 5.39E-07 |
| PH | FarmCPU | 2016 | Kukri_c34553_89 | 2B | 887912 | 7.49E-07 |
| PH | FarmCPU | 2016 | IAAV8023 | 5B | 2764311 | 1.46E-06 |
| PH | FarmCPU | 2016 | BS00065020_51 | 7A | 3559454 | 3.94E-06 |
| PH | FarmCPU | 2016 | BobWhite_2514_109 | 7B | 4216848 | 4.21E-06 |
| PH | FarmCPU | 2016 | Kukri_04598_614 | 2A | 87562 | 4.29E-06 |
| PH | FarmCPU | 2016 | wsnp_Ra_c407_862316 | 2B | 449283 | 2.42E-05 |
| PH | FarmCPU | 2017 | RAC875_03931_205 | 5A | 2358890 | 8.95E-11 |
| PH | FarmCPU | 2017 | Kukri_c9571_73 | 3B | 1433517 | 1.20E-10 |
| PH | FarmCPU | 2017 | BS00049008_51 | 3B | 1427924 | 2.75E-10 |
| PH | FarmCPU | 2017 | RAC875_c87421_408 | 2B | 603716 | 3.22E-08 |
| PH | FarmCPU | 2017 | Tdurum_contig10861_942 | 7B | 3924373 | 1.21E-07 |
| PH | FarmCPU | 2017 | GENE_4469_430 | 7A | 3542226 | 5.24E-07 |
| PH | FarmCPU | 2017 | BS00094333_51 | 5D | 2898105 | 1.38E-06 |
| PH | FarmCPU | 2017 | wsnp_Ra_c6374_11143280 | 5B | 2720382 | 2.94E-06 |
| PH | FarmCPU | 2017 | RAC875_c8121_1490 | 3A | 1272572 | 5.60E-06 |
| PH | FarmCPU | 2017 | Kukri_23338_624 | 4B | 2024238 | 5.69E-06 |
| PH | FarmCPU | 2017 | Jagger_c6772_80 | 1A | 408 | 1.30E-05 |
| PH | FarmCPU | 2017 | wsnp_Ex_28733_37836638 | 1B | 789 | 2.22E-05 |
| PH | FarmCPU | 2018 | BS00049008_51 | 3B | 1427924 | 6.81E-09 |
| PH | FarmCPU | 2018 | Excalibur_c7377_445 | 4B | 2021367 | 2.06E-07 |
| PH | FarmCPU | 2018 | RAC875_c8121_1490 | 3A | 1272572 | 7.36E-07 |
| PH | FarmCPU | 2018 | RAC875_c33248_246 | 5B | 2500545 | 2.43E-06 |
| PH | FarmCPU | 2018 | IACX9410 | 5A | 2355287 | 1.53E-05 |
| PH | FarmCPU | 2018 | wsnp_Ex_25573_34834321 | 3B | 1536039 | 1.99E-05 |
| PH | FarmCPU | 2018 | Tdurum_contig13837_573 | 2A | 309166 | 2.20E-05 |
| PH | FarmCPU | 2018 | Tdurum_contig11028_398 | 7B | 3939359 | 2.25E-05 |
| PH | MLM | 2015 | BS00110445_51 | 3B | 1443736 | 4.26E-05 |
| PH | MLM | 2016 | tplb0049a09_1302 | 5A | 2453837 | 2.14E-05 |
| PH | MLM | 2016 | BobWhite_c8266_227 | 5A | 2455941 | 2.82E-05 |
| PH | MLM | 2016 | wsnp_CAP7_00100_4479468 | 7A | 3553845 | 2.98E-05 |
| PH | MLM | 2016 | RAC875_c8121_1490 | 3A | 1272572 | 3.18E-05 |
| PH | MLM | 2016 | BS00023152_51 | 5A | 2450348 | 4.08E-05 |
| PH | MLM | 2017 | RAC875_c8121_1490 | 3A | 1272572 | 6.55E-06 |
| PH | MLM | 2017 | BS00049008_51 | 3B | 1427924 | 1.28E-05 |
| PH | MLM | 2017 | BS00023152_51 | 5A | 2450348 | 1.51E-05 |
| PH | MLM | 2017 | tplb0049a09_1302 | 5A | 2453837 | 1.68E-05 |
| PH | MLMM | 2015 | BS00110445_51 | 3B | 1443736 | 2.23E-05 |
| PH | MLMM | 2015 | IACX3190 | 3B | 1429597 | 2.32E-05 |
| PH | MLMM | 2015 | BS00049008_51 | 3B | 1427924 | 2.68E-05 |
| PH | MLMM | 2015 | Excalibur_rep_008066_112 | 5A | 2179813 | 2.73E-05 |
| PH | MLMM | 2015 | BobWhite_24364_73 | 3B | 1551850 | 4.27E-05 |
| PH | MLMM | 2016 | tplb0049a09_1302 | 5A | 2453837 | 1.24E-05 |
| PH | MLMM | 2016 | BobWhite_c8266_227 | 5A | 2455941 | 1.68E-05 |
| PH | MLMM | 2016 | wsnp_CAP7_00100_4479468 | 7A | 3553845 | 1.79E-05 |
| PH | MLMM | 2016 | RAC875_c8121_1490 | 3A | 1272572 | 1.92E-05 |
| PH | MLMM | 2016 | BS00023152_51 | 5A | 2450348 | 2.54E-05 |
| PH | MLMM | 2017 | RAC875_c8121_1490 | 3A | 1272572 | 3.21E-06 |
| PH | MLMM | 2017 | BS00049008_51 | 3B | 1427924 | 6.86E-06 |
| PH | MLMM | 2017 | BS00023152_51 | 5A | 2450348 | 8.29E-06 |
| PH | MLMM | 2017 | tplb0049a09_1302 | 5A | 2453837 | 9.35E-06 |
| PH | MLMM | 2017 | IACX3190 | 3B | 1429597 | 3.61E-05 |
| PH | MLMM | 2018 | RAC875_c8121_1490 | 3A | 1272572 | 3.18E-05 |
| FLL | FarmCPU | 2017 | Tdurum_contig42755_1086 | 1B | 770 | 3.63E-06 |
| FLL | FarmCPU | 2017 | Ra_c4614_1114 | 4A | 1728376 | 4.46E-06 |
| FLL | FarmCPU | 2017 | wsnp_Ex_c8409_14170476 | 3A | 1035380 | 4.83E-06 |
| FLL | FarmCPU | 2017 | wsnp_JD_rep_c64505_41132927 | 2B | 577202 | 5.60E-06 |
| FLL | FarmCPU | 2017 | tplb0049a09_1302 | 5A | 2453837 | 8.98E-06 |
| FLL | FarmCPU | 2017 | wsnp_Ex_c61603_61581245 | 7A | 3834679 | 9.90E-06 |
| FLL | FarmCPU | 2017 | wsnp_Ku_08538_27857915 | 3B | 1393911 | 1.02E-05 |
| FLL | FarmCPU | 2017 | BS00078603_51 | 5D | 2897054 | 1.05E-05 |
| FLL | FarmCPU | 2017 | BobWhite_04066_403 | 6D | 3496149 | 1.19E-05 |
| FLL | FarmCPU | 2017 | wsnp_Ex_c3930_7127883 | 7B | 4227010 | 3.81E-05 |
| FLL | FarmCPU | 2018 | Kukri_rep_c80571_265 | 5A | 2166418 | 3.25E-07 |
| FLL | FarmCPU | 2018 | IAAV7013 | 3D | 1703806 | 7.79E-06 |
| FLL | FarmCPU | 2018 | IACX6009 | 5B | 2609592 | 1.01E-05 |
| FLL | FarmCPU | 2018 | wsnp_Ex_28733_37836638 | 1B | 789 | 1.68E-05 |
| FLL | FarmCPU | 2018 | Ku_c8125_1049 | 6A | 2969583 | 1.95E-05 |
| FLL | FarmCPU | 2018 | JD_2708_1512 | 7D | 4326677 | 2.28E-05 |
| FLL | FarmCPU | 2018 | Excalibur_rep_003892_1107 | 5D | 2850819 | 4.09E-05 |
| FLL | FarmCPU | 2018 | BS00079185_51 | 5B | 2480071 | 4.17E-05 |
| FLL | MLM | 2017 | tplb0049a09_1302 | 5A | 2453837 | 1.58E-06 |
| FLL | MLM | 2017 | Excalibur_c46261_342 | 5A | 2452438 | 5.07E-06 |
| FLL | MLM | 2017 | tplb0049a09_2097 | 5A | 2454537 | 1.89E-05 |
| FLL | MLM | 2018 | RAC875_c8121_1490 | 3A | 1272572 | 6.17E-06 |
| FLL | MLM | 2018 | tplb0049a09_1302 | 5A | 2453837 | 6.42E-06 |
| FLL | MLM | 2018 | Excalibur_c46261_342 | 5A | 2452438 | 1.46E-05 |
| FLL | MLM | 2018 | tplb0049a09_2097 | 5A | 2454537 | 1.89E-05 |
| FLL | MLMM | 2017 | tplb0049a09_1302 | 5A | 2453837 | 1.99E-07 |
| FLL | MLMM | 2017 | wsnp_Ex_c61603_61581245 | 7A | 3834679 | 3.44E-05 |
| FLL | MLMM | 2018 | RAC875_c8121_1490 | 3A | 1272572 | 8.82E-07 |
| FLW | MLM | 2017 | Excalibur_01167_1207 | 5A | 2153585 | 3.71E-05 |
| FLW | MLM | 2018 | wsnp_Ku_c6977_12078791 | 5A | 2367991 | 3.30E-05 |
| FLW | MLMM | 2017 | Excalibur_01167_1207 | 5A | 2153585 | 2.25E-05 |
| FLW | MLMM | 2018 | wsnp_Ku_c6977_12078791 | 5A | 2367991 | 2.00E-05 |
| TILL | FarmCPU | 2018 | BobWhite_07783_174 | 2A | 359541 | 3.06E-05 |
| TILL | MLMM | 2018 | BobWhite_07783_174 | 2A | 359541 | 3.64E-05 |

Abbreviations: PH (Plant height), FLL (Flag leaf length), FLW (Flag leaf width), TILL (Tillers per plant)

**Table S5.** Common SNPS detected through ML-GAWS and SL-GWAS methods.

| **Trait** | **Methods** | **SNP** | **Chr** | **Position (bp)** |
| --- | --- | --- | --- | --- |
| PH | pLARmEB, FarmCPU | Jagger_c6772_80 | 1A | 408 |
| PH | pLARmEB, FarmCPU | Kukri_04598_614 | 2A | 87562 |
| PH | mrMLM, FarmCPU | Kukri_c34553_89 | 2B | 887912 |
| PH | pLARmEB, FarmCPU | RAC875_c8121_1490 | 3A | 1272572 |
| PH | FASTmrEMMA, MLMM | IACX3190 | 3B | 1429597 |
| PH | pLARmEB, MLM | BS00110445_51 | 3B | 1443736 |
| PH | pLARmEB, FarmCPU | wsnp_Ex_25573_34834321 | 3B | 1536039 |
| PH | MLMM, mrMLM | BobWhite_24364_73 | 3B | 1551850 |
| PH | FASTmrEMMA, FarmCPU | Excalibur_rep_008066_112 | 5A | 2179813 |
| PH | pLARmEB, FarmCPU | wsnp_Ex_rep_c66315_64480362 | 6B | 3350804 |
| PH | pLARmEB, FarmCPU | GENE_4469_430 | 7A | 3542226 |
| FLL | FarmCPU, mrMLM | wsnp_Ex_28733_37836638 | 1B | 789 |
| FLL | MLMM, pLARmEB | RAC875_c8121_1490 | 3A | 1272572 |
| FLL | MLMM, mrMLM | tplb0049a09_1302 | 5A | 2453837 |
| FLL | MLM, FASTmrEMMA | tplb0049a09_2097 | 5A | 2454537 |
| FLL | MLMM, mrMLM | wsnp_Ex_c61603_61581245 | 7A | 3834679 |
| FLW | MLMM, mrMLM | Excalibur_01167_1207 | 5A | 2153585 |
| FLW | MLMM, mrMLM | wsnp_Ku_c6977_12078791 | 5A | 2367991 |
| TILL | FASTmrEMMA, MLMM | BobWhite_07783_174 | 2A | 359541 |

Abbreviations: PH (Plant height), FLL (Flag leaf length), FLW (Flag leaf width), TILL (Tillers per plant)

**Table S6.** List of pleiotropic SNPs associated with more than one trait.

| **SNP** | **Chromosome** | **Position (bp)** | **Traits** |
| --- | --- | --- | --- |
| Jagger_c6772_80 | 1A | 408 | PH, FLL |
| RAC875_c8121_1490 | 3A | 1272572 | PH, FLL |
| BS00089954_51 | 3B | 1428203 | PH, FLL |
| Excalibur_01167_1207 | 5A | 2153585 | PH, FLW |
| Ku_c5191_340 | 6B | 3397322 | PH, FLL |

Abbreviations: PH (Plant height), FLL (Flag leaf length), FLW (Flag leaf width).
